# Supplementary material for: Molecular Pincers Using a Combination of N-H and C-H Donors for Anion Binding
Source: Int J Mol Sci. 2022 Dec 22;24(1):163. doi: 10.3390/ijms24010163 (PMC9820443; doi:10.3390/ijms24010163)
Supplement: Supplementary file 1 [file ijms-24-00163-s001.zip › ijms-2105972-supplementary.pdf]

Supporting information for

Molecular Pincers Using a Combination of N-H and C-H Donors for Anion Binding

Jaehyeon Kim [1,†], Seung Hyeon Kim [1,†], Nam Jung Heo [1], Benjamin P. Hay [2] and Sung Kuk Kim [1,*]

[1] Department of Chemistry and Research Institute of Natural Science, Gyeongsang National University, Jinju 52828, Republic of Korea
[2] Supramolecular Design Institute, Oak Ridge, TN 37830, USA
* Correspondence: sungkukkim@gnu.ac.kr
† These authors contributed equally to this work.

Table of contents

1. NMR spectral data S2 – S17

2. Fluorescence spectral data S18 – S29

3. NMR spectral and HRMS data S30- S41

4. Supporting references S42

5. Molecular mechanics computation data S43 – S66

1. ^{1}H NMR spectra data

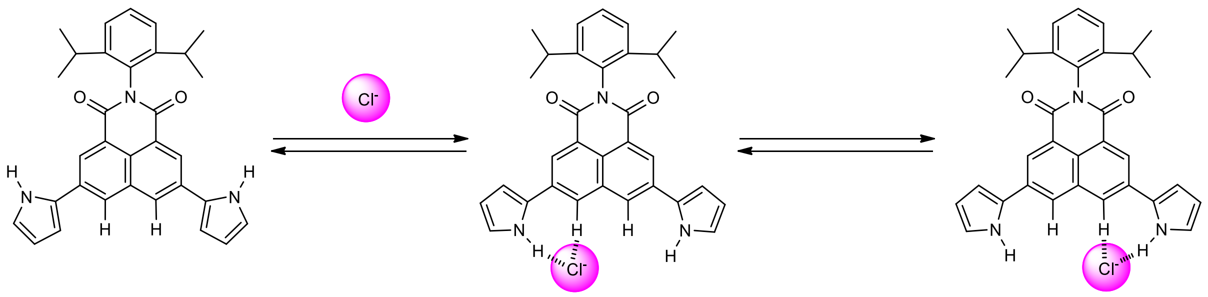

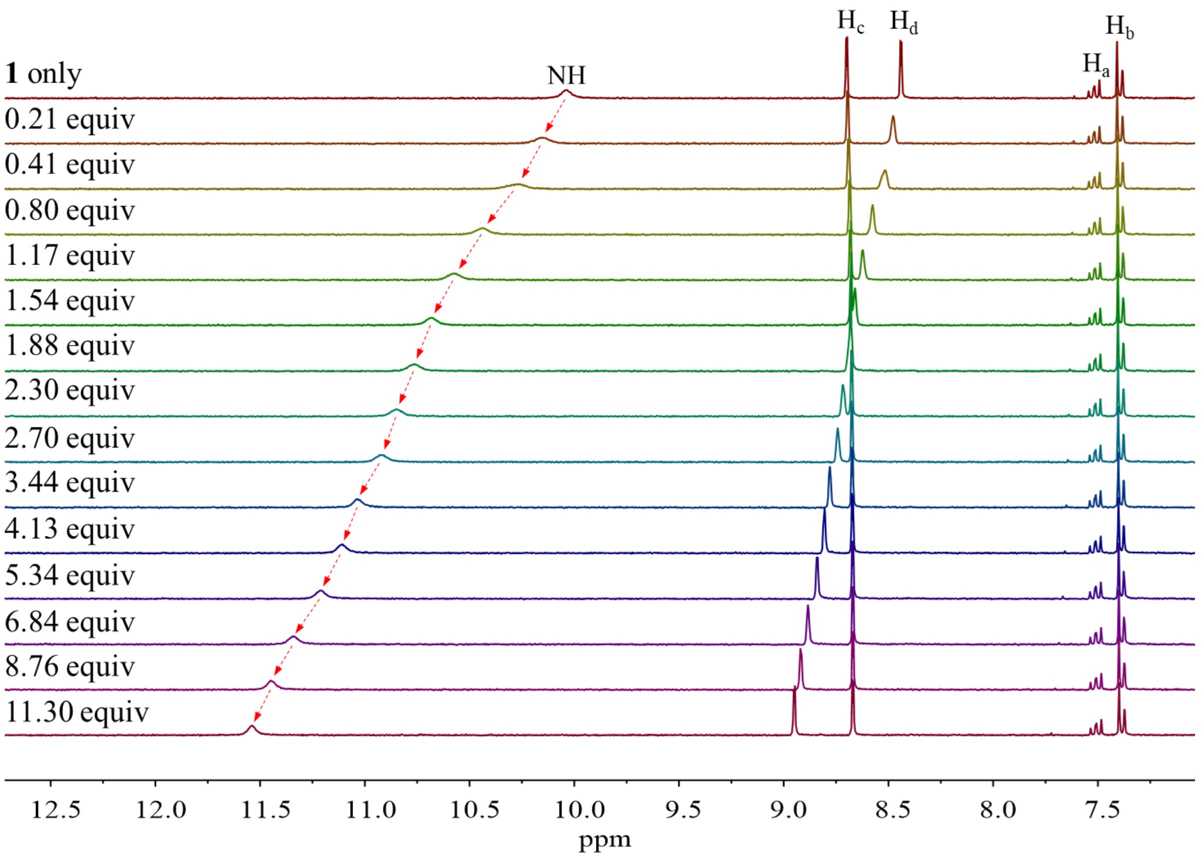

Figure S1. Top: Proposed interaction modes between receptor **1** and the chloride anion. Bottom: Partial ^{1}H NMR spectra recorded during the titration of receptor **1** (3 mM) with tetrabutylammonium chloride (TBACl) in CD_3CN.

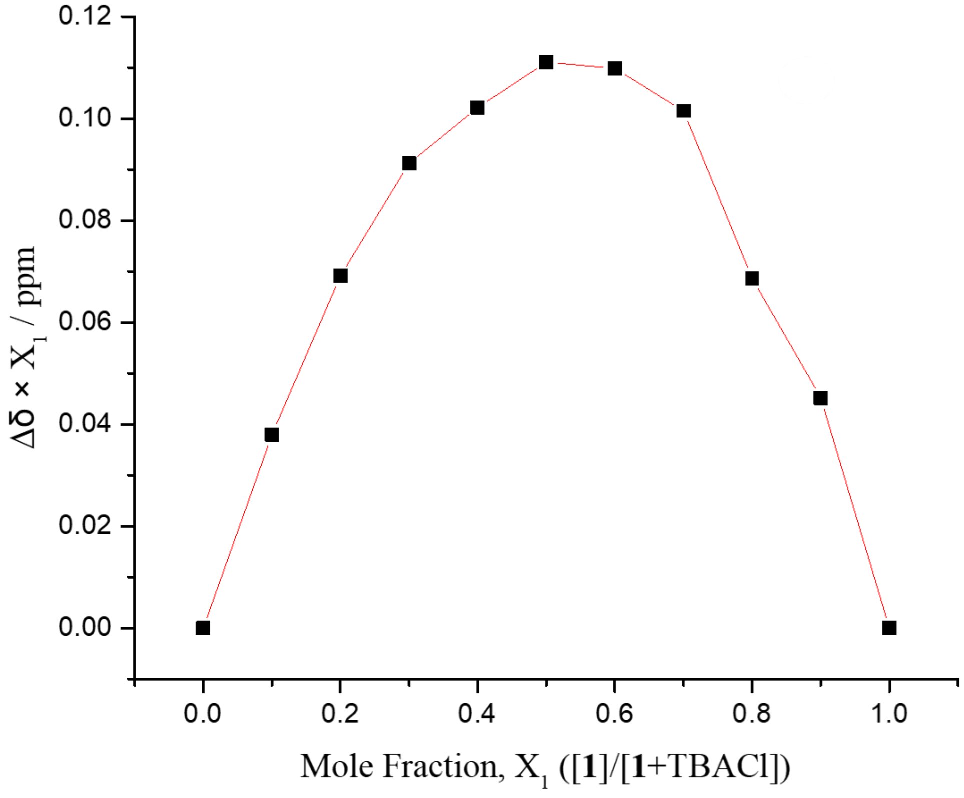

Figure S2. Job's plot for the interaction of receptor **1** with TBACl in CD_3CN.

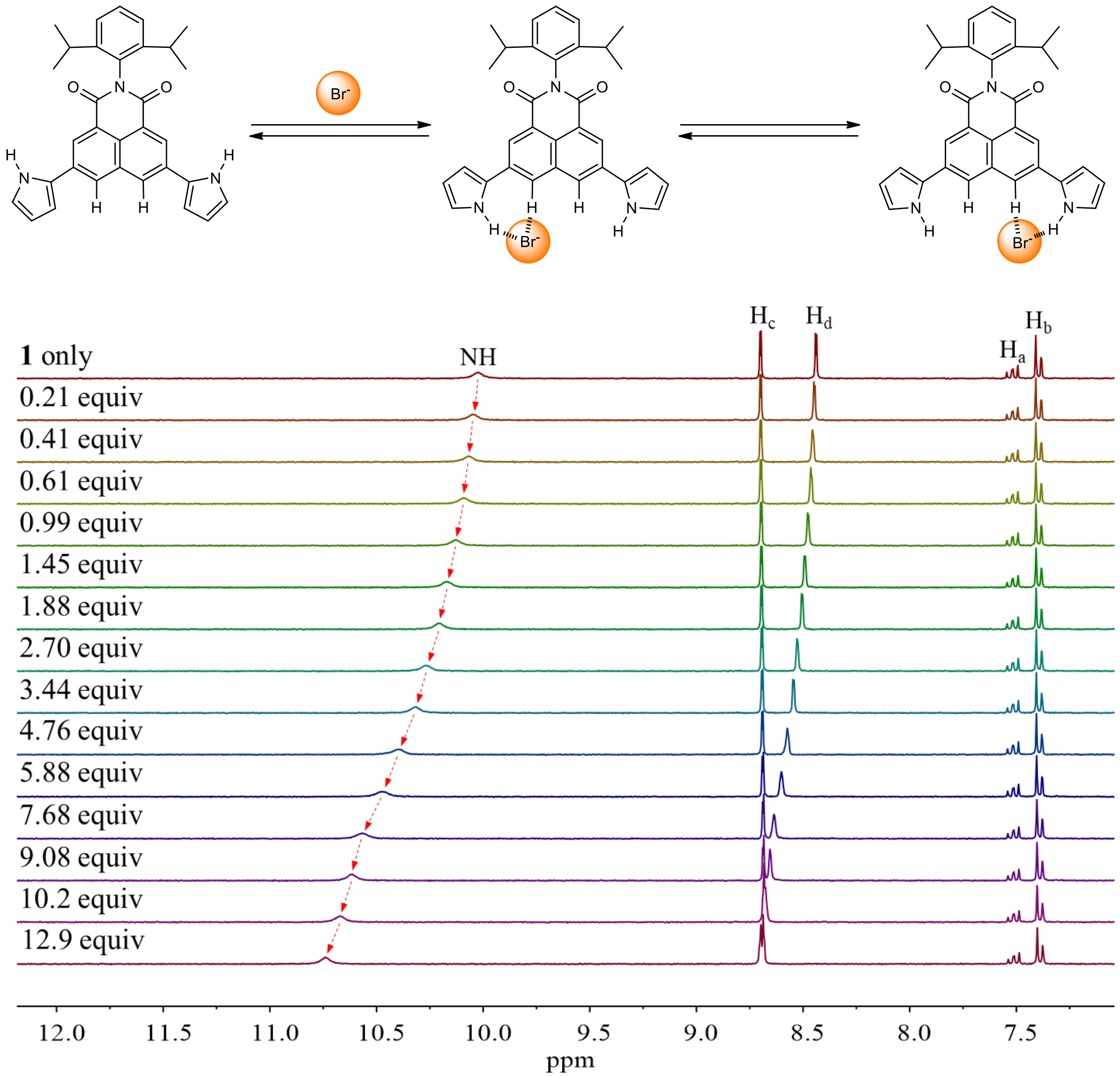

Figure S3. Top: Proposed interaction modes between receptor **1** and the bromide anion. Bottom: Partial ^{1}H NMR spectra recorded during the titration of receptor **1** (3 mM) with tetrabutylammonium bromide (TBABr) in CD_3CN.

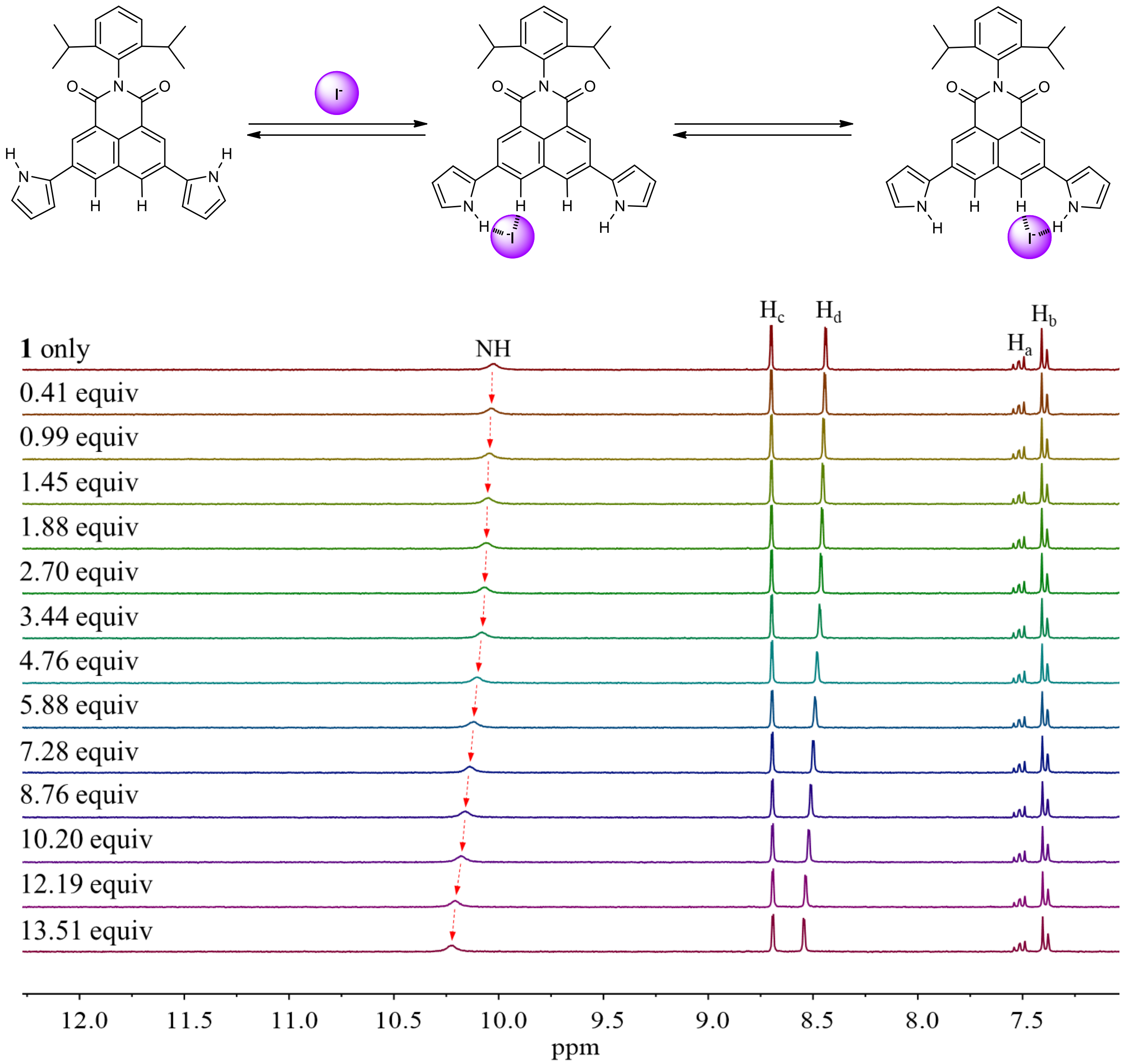

Figure S4. Top: Proposed interaction modes between receptor **1** and the iodide anion. Bottom: Partial ^1H NMR spectra recorded during the titration of receptor **1** (3 mM) with tetrabutylammonium iodide (TBAI) in CD_3CN.

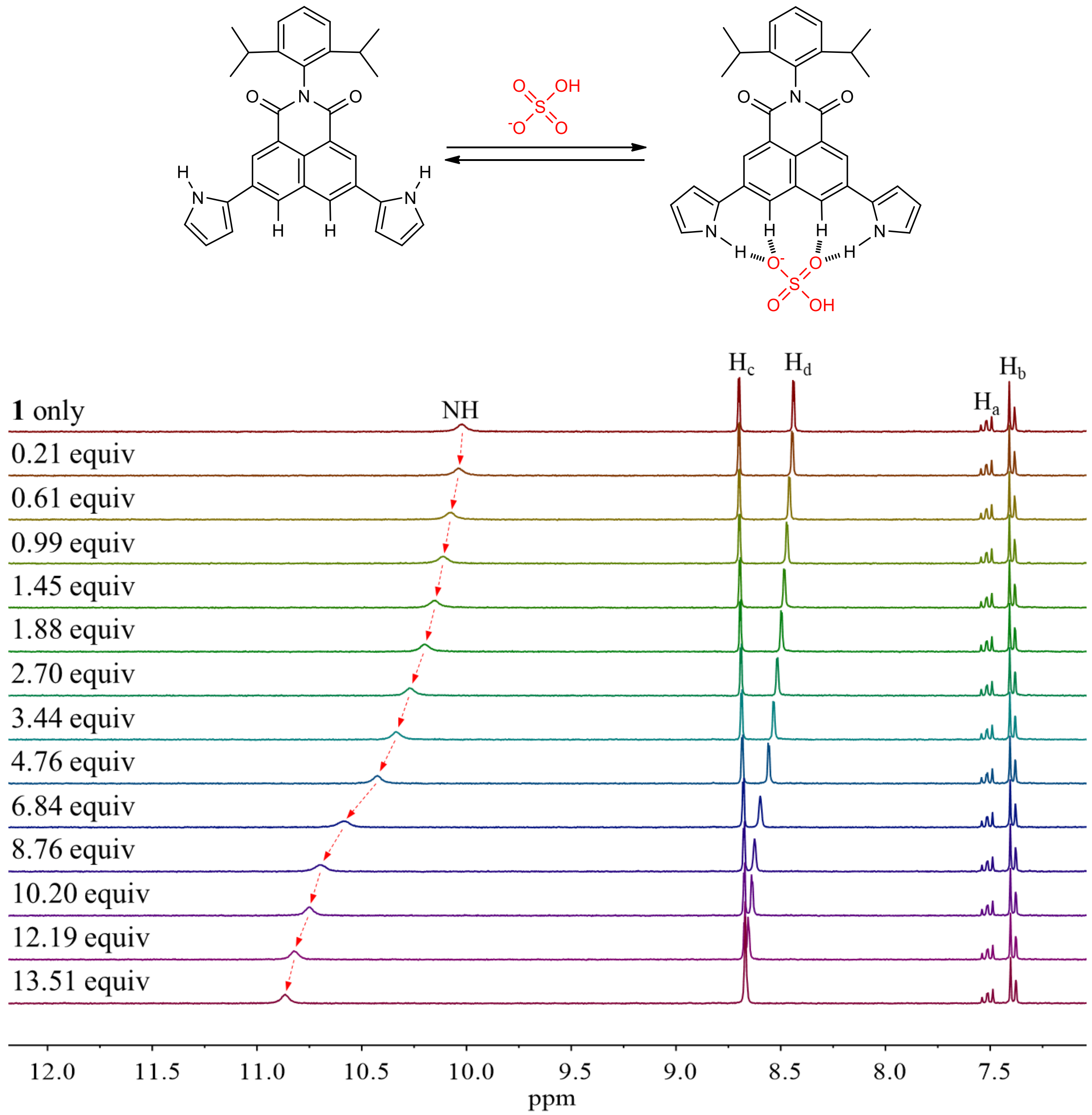

Figure S5. Top: Proposed interaction modes between receptor **1** and the hydrogen sulfate anion. Bottom: Partial ^{1}H NMR spectra recorded during the titration of receptor **1** (3 mM) with tetrabutylammonium hydrogen sulfate ($TBAHSO_4$) in CD_3CN.

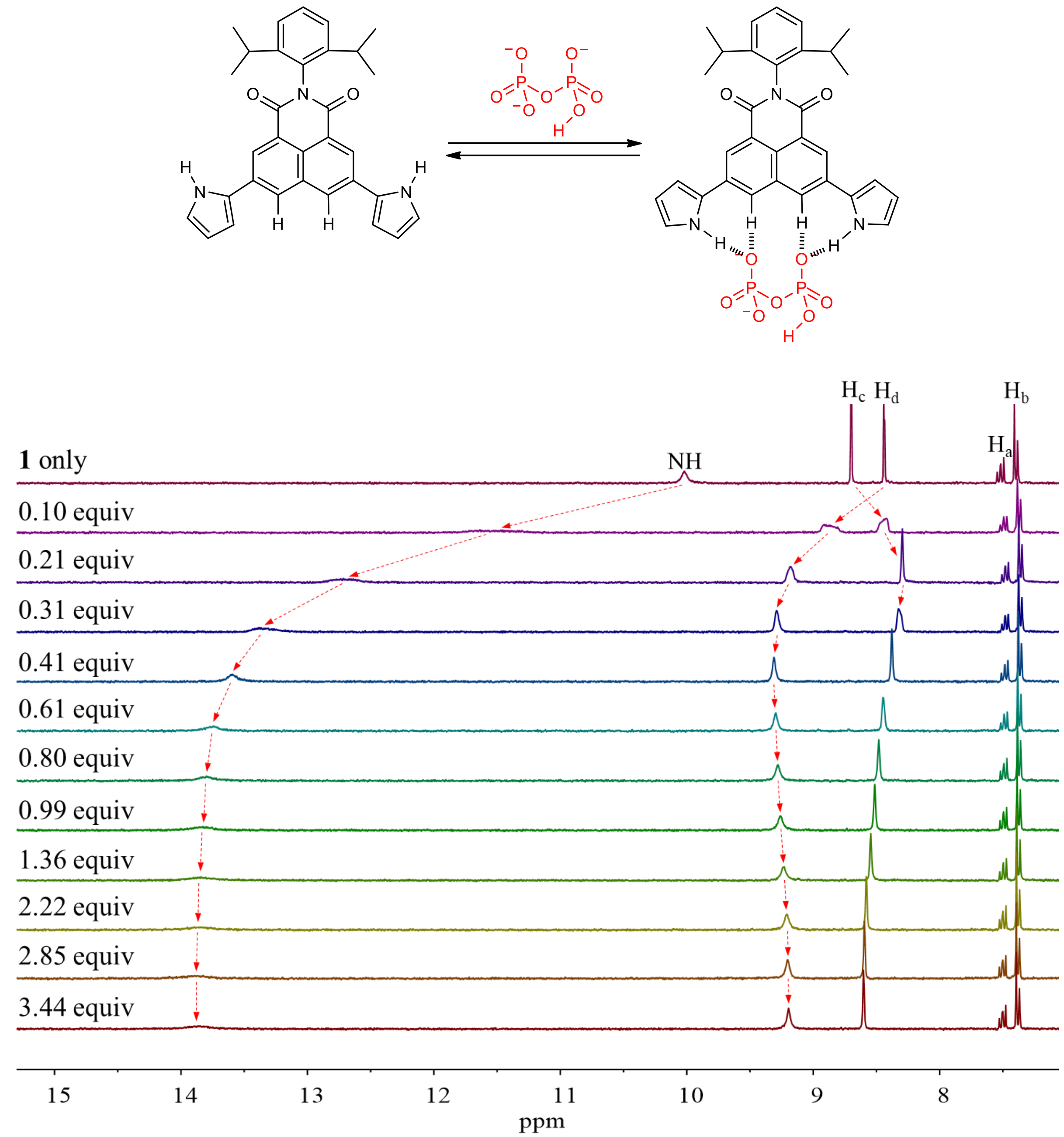

Figure S6. Top: Proposed interaction modes between receptor **1** and the hydrogen pyrophosphate anion. Bottom: Partial ^{1}H NMR spectra recorded during the titration of receptor **1** (3 mM) with *tris*-tetrabutylammonium hydrogen pyrophosphate ($(TBA)_3HP_2O_7$) in CD_3CN.

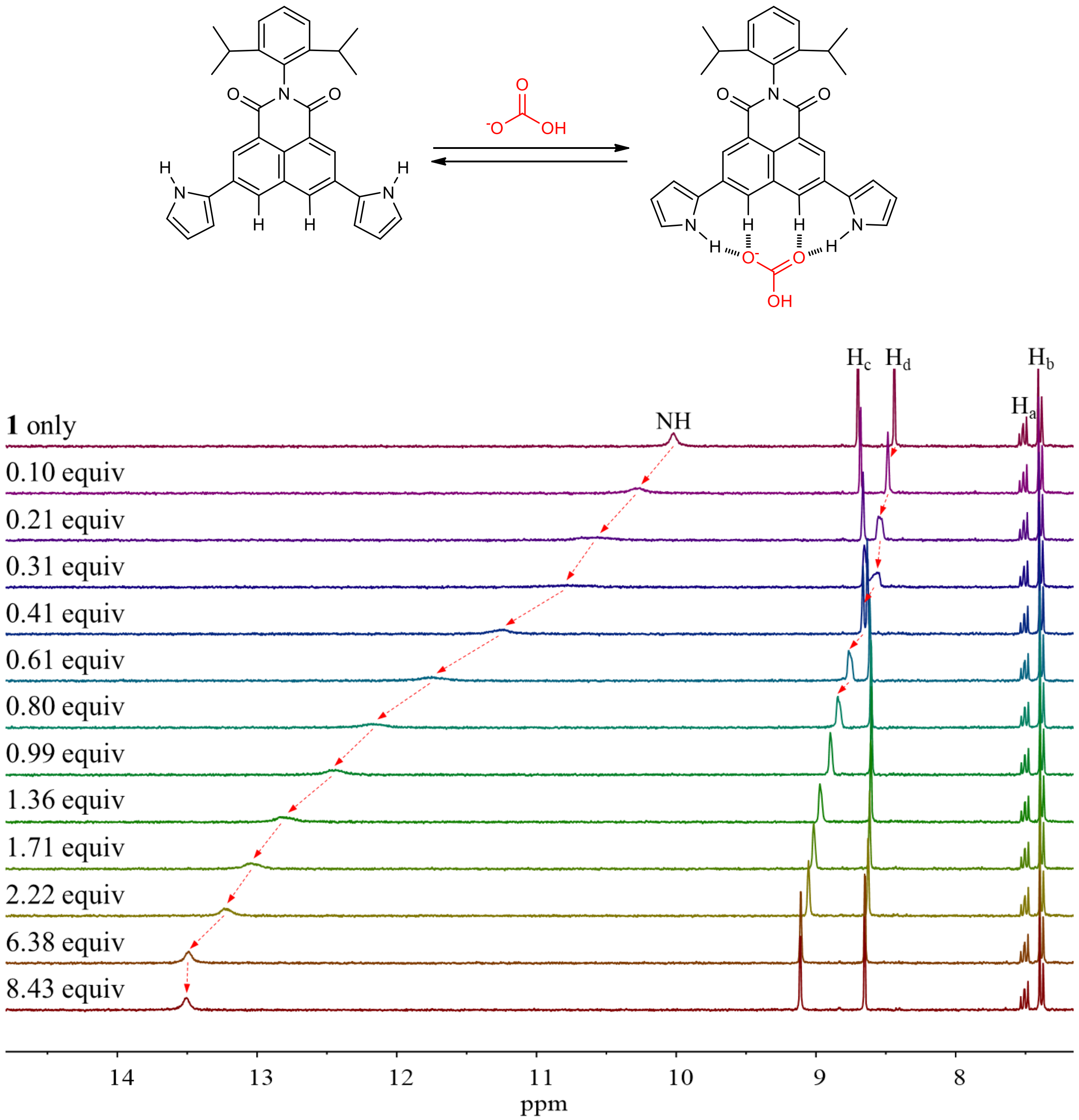

Figure S7. Top: Proposed interaction modes between receptor **1** and the bicarbonate anion. Bottom: Partial ^{1}H NMR spectra recorded during the titration of receptor **1** (3 mM) with tetraetylammonium bicarbonate (TEAHCO3) in CD3CN.

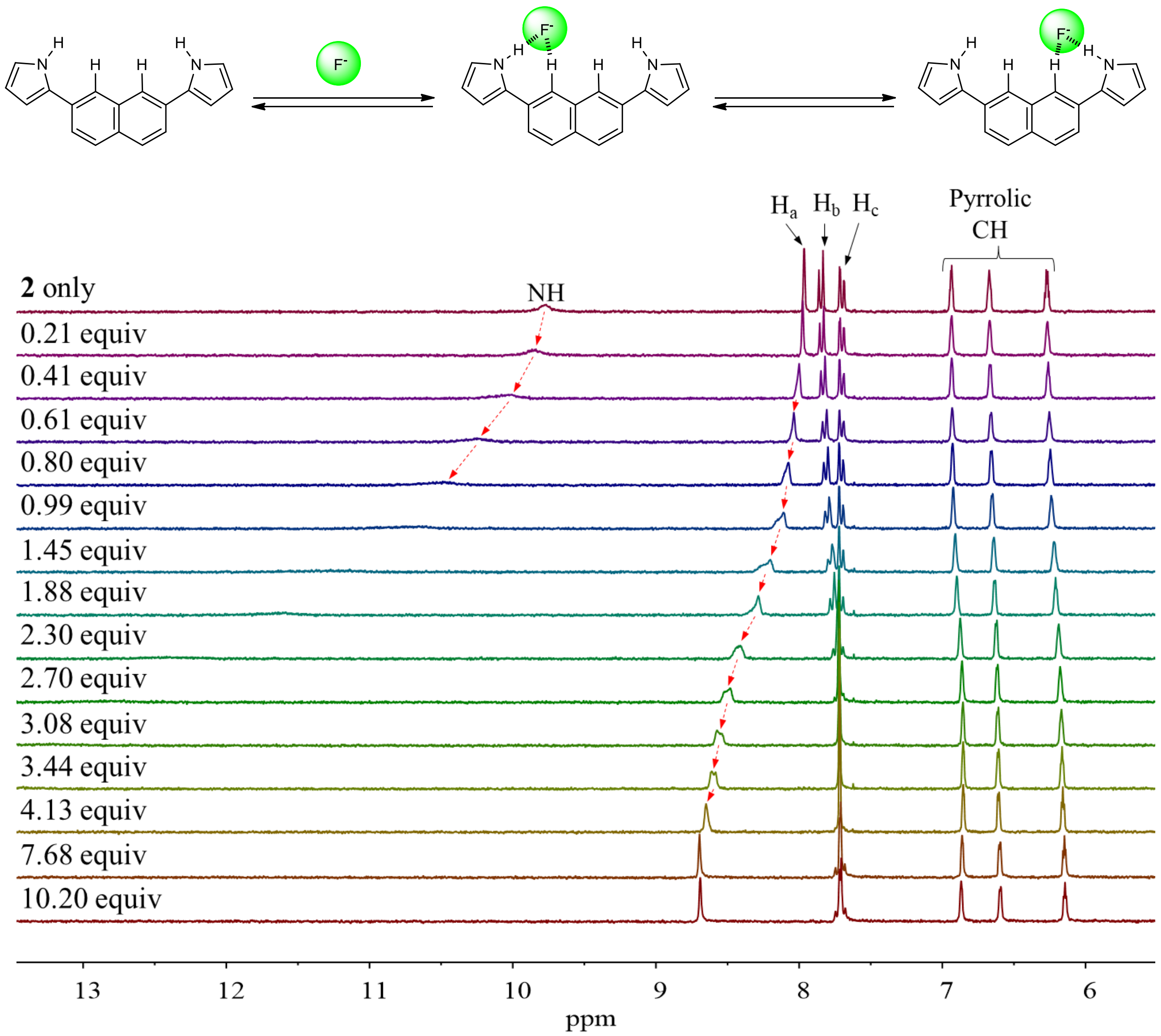

Figure S8. Top: Proposed interaction modes between receptor **2** and the fluoride anion. Bottom: Partial ^{1}H NMR spectra recorded during the titration of receptor **2** (3 mM) with tetrabutylammonium fluoride (TBAF) in CD_3CN.

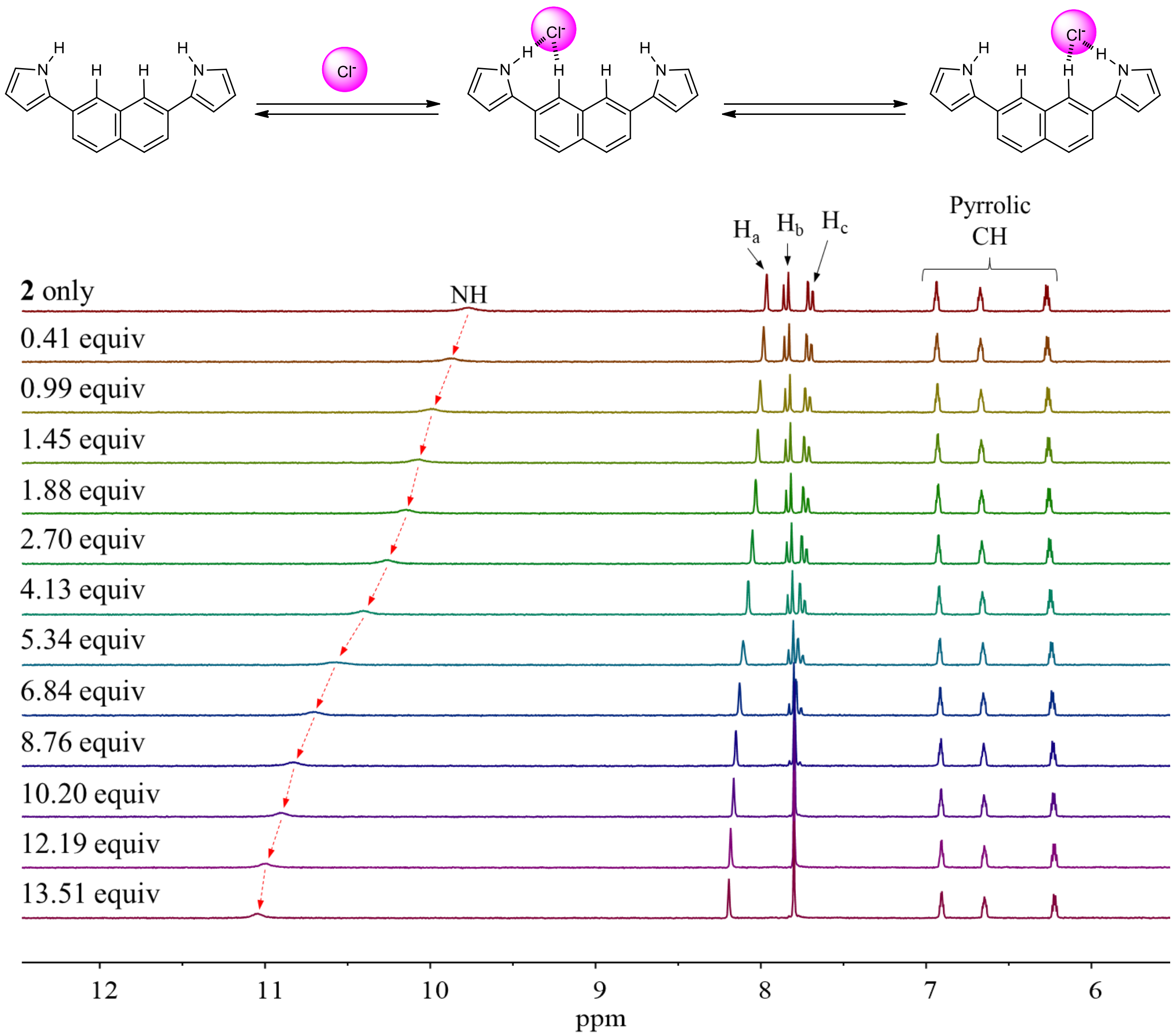

Figure S9. Top: Proposed interaction modes between receptor **2** and the chloride anion. Bottom: Partial ^{1}H NMR spectra recorded during the titration of receptor **2** (3 mM) with tetrabutylammonium chloride (TBACl) in CD_3CN.

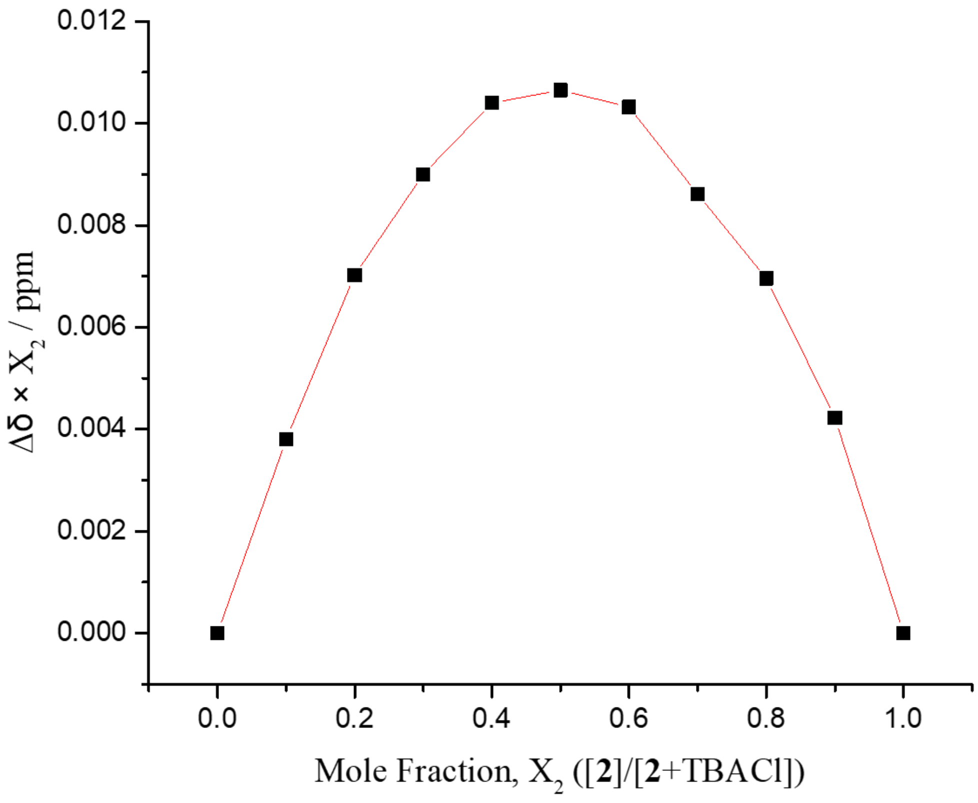

Figure S10. Job's plot for the interaction of receptor **2** with TBACl in CD_3CN.

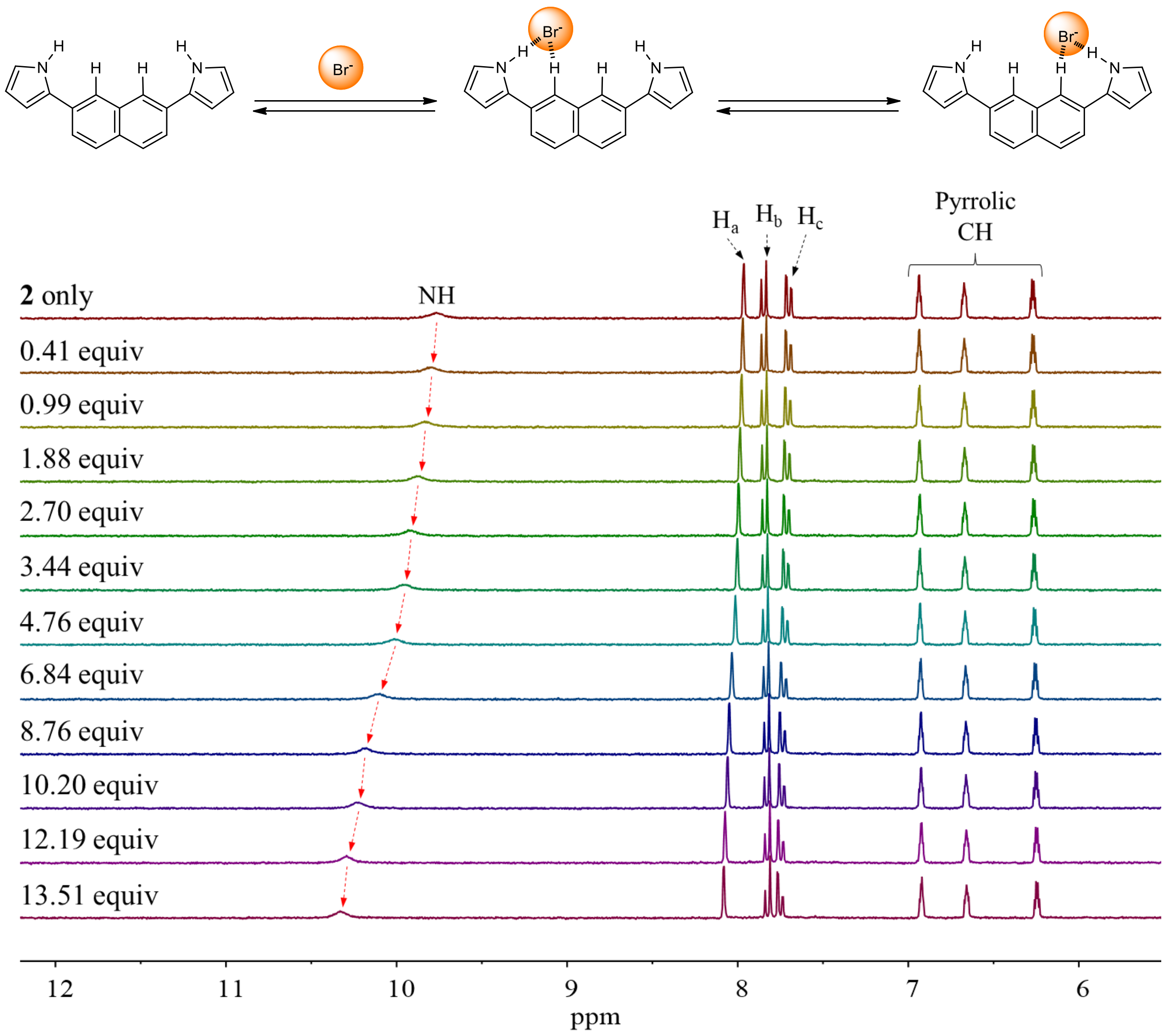

Figure S11. Top: Proposed interaction modes between receptor **2** and the bromide anion. Bottom: Partial ^{1}H NMR spectra recorded during the titration of receptor **2** (3 mM) with tetrabutylammonium bromide (TBABr) in CD_3CN.

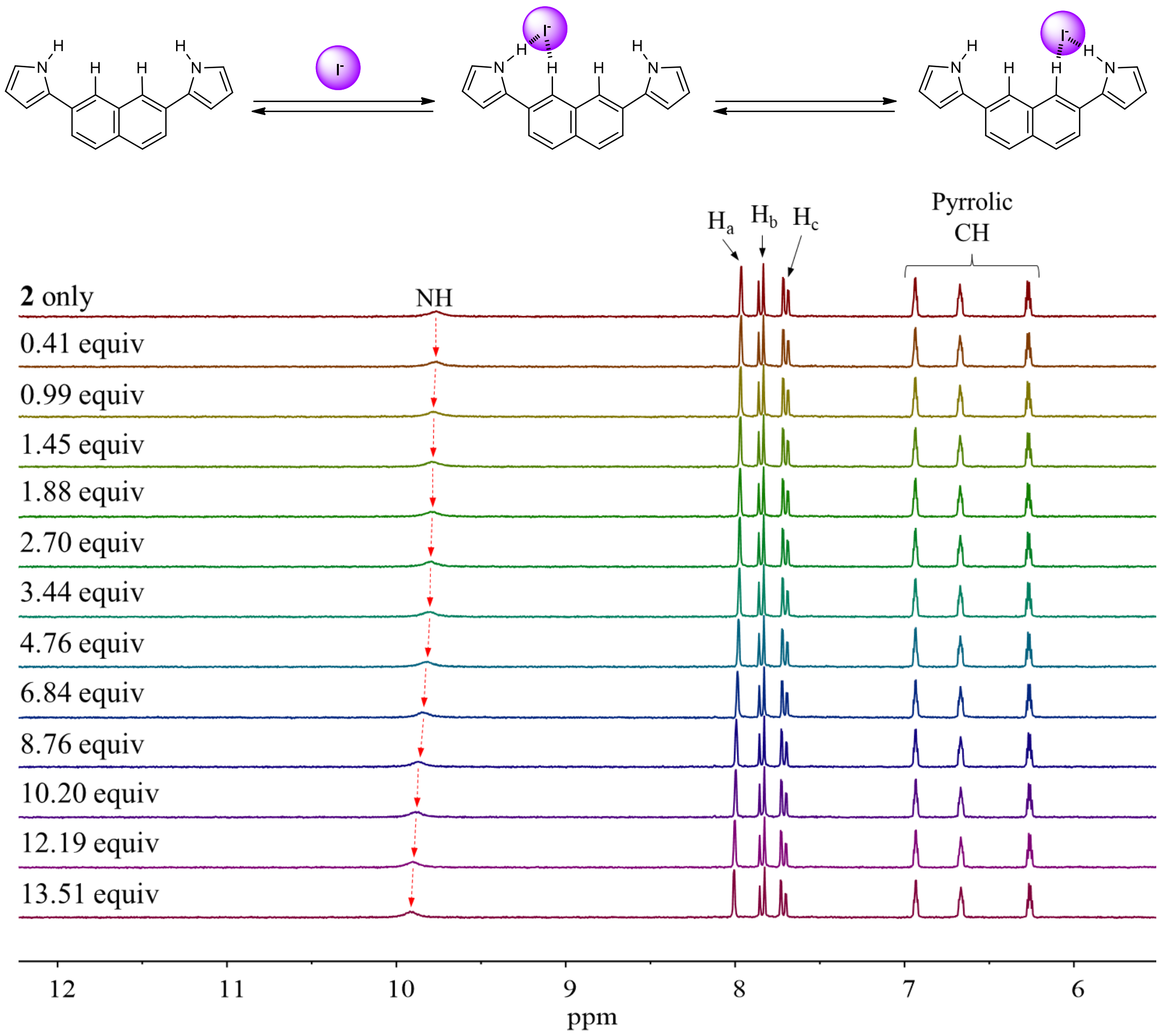

Figure S12. Top: Proposed interaction modes between receptor **2** and the iodide anion. Bottom: Partial ^{1}H NMR spectra recorded during the titration of receptor **2** (3 mM) with tetrabutylammonium iodide (TBAI) in CD_3CN.

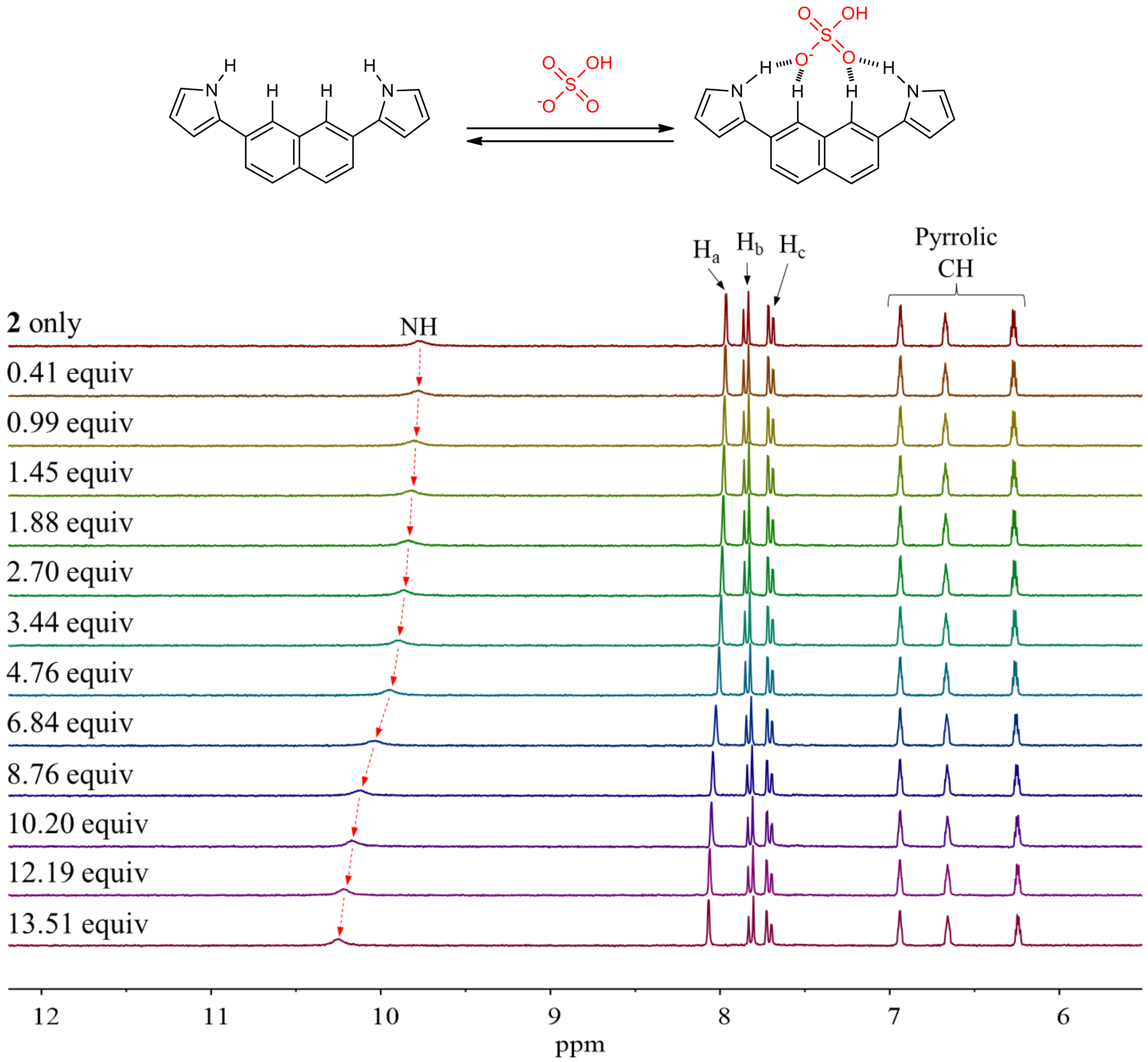

Figure S13. Top: Proposed interaction modes between receptor **2** and the hydrogen sulfate anion. Bottom: Partial ^{1}H NMR spectra recorded during the titration of receptor **2** (3 mM) with tetrabutylammonium hydrogen sulfate (TBAHSO4) in CD_3CN.

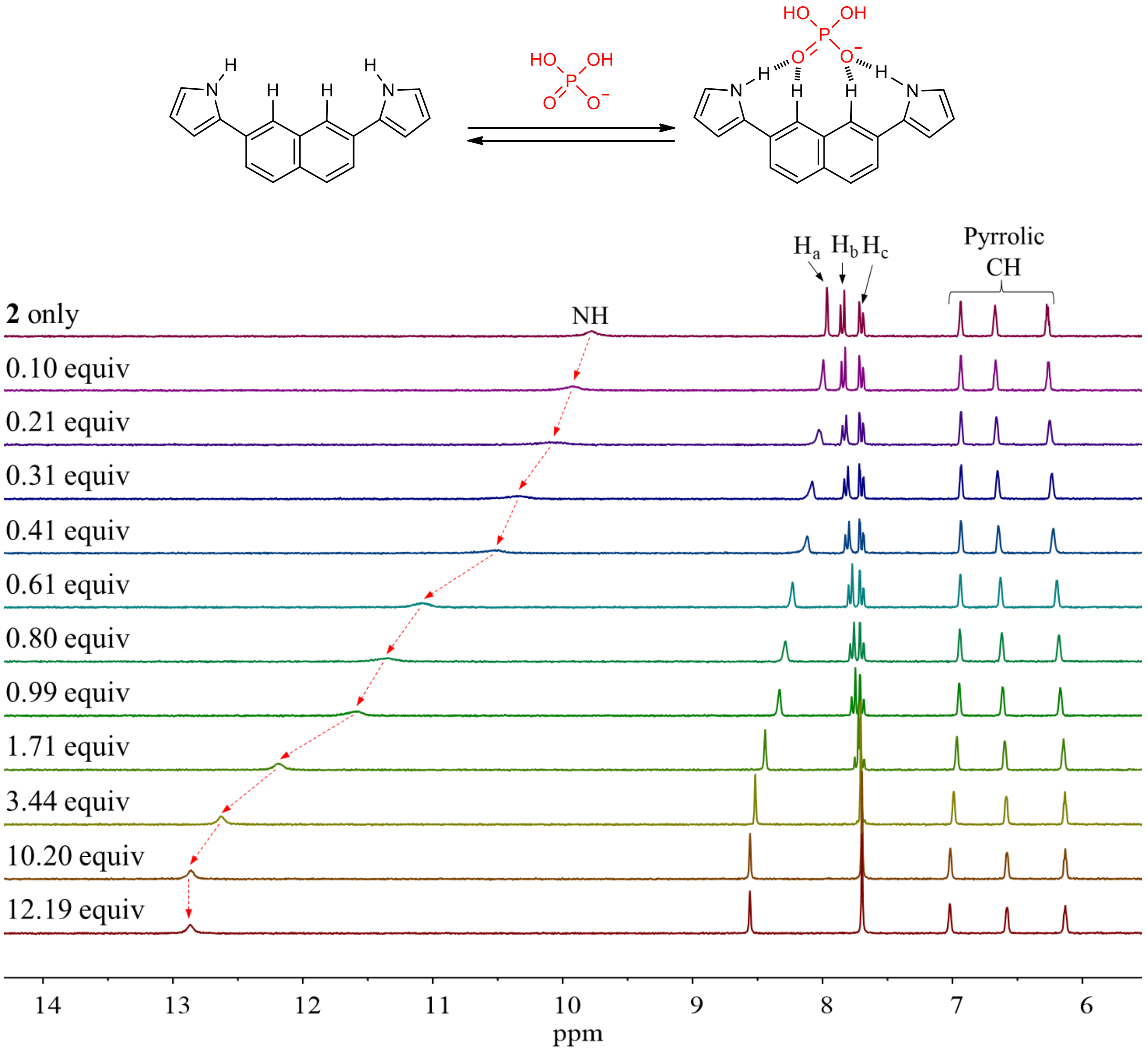

Figure S14. Top: Proposed interaction modes between receptor **2** and the dihydrogen phosphate anion. Bottom: Partial ^{1}H NMR spectra recorded during the titration of receptor **2** (3 mM) with tetrabutylammonium dihydrogen phosphate ($TBAH_2PO_4$) in CD_3CN.

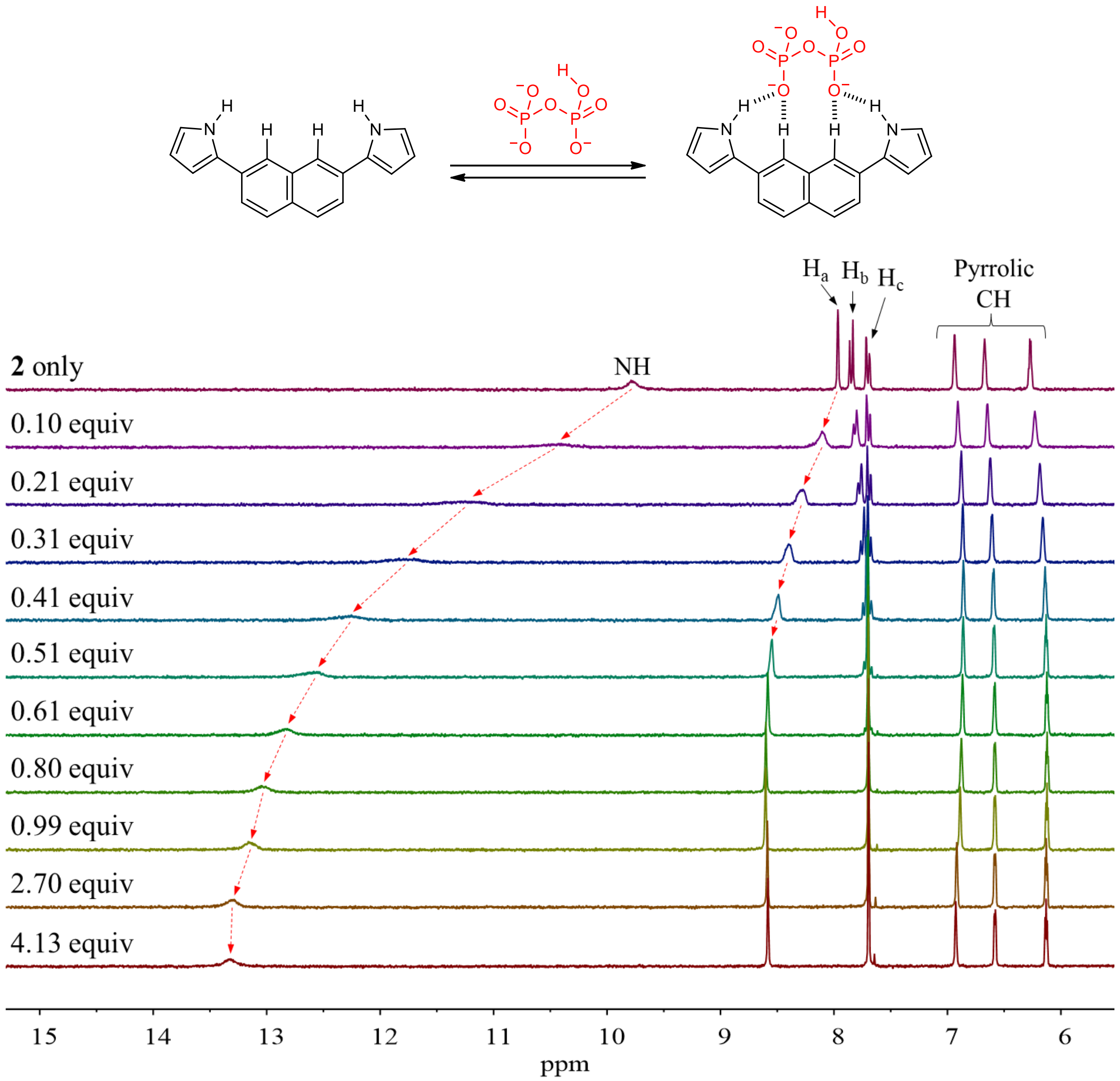

Figure S15. Top: Proposed interaction modes between receptor **2** and the hydrogen pyrophosphate anion. Bottom: Partial ^{1}H NMR spectra recorded during the titration of receptor **2** (3 mM) with *tris*-tetrabutylammonium hydrogen pyrophosphate ($(TBA)_3HP_2O_7$) in CD_3CN.

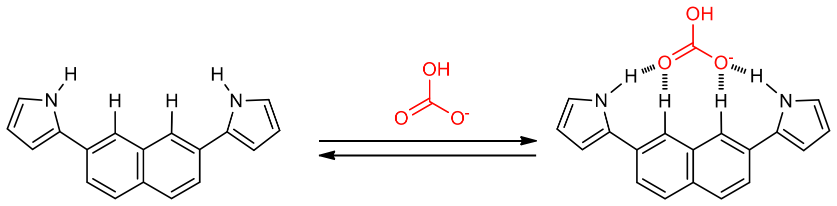

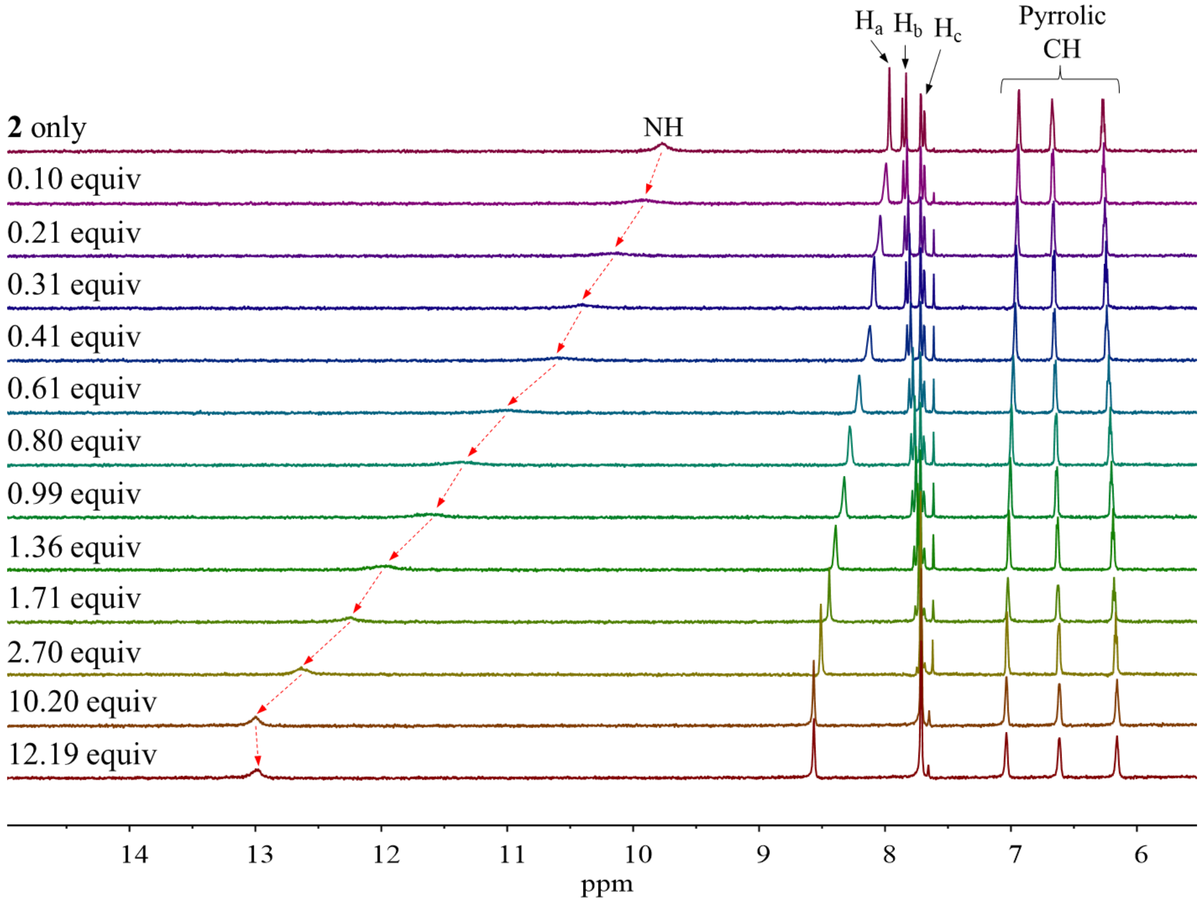

Figure S16. Top: Proposed interaction modes between receptor **2** and the bicarbonate anion. Bottom: Partial ^{1}H NMR spectra recorded during the titration of receptor **2** (3 mM) with tetraetylammonium bicarbonate (TEAHCO3) in CD_3CN.

2. Fluorescence spectral data

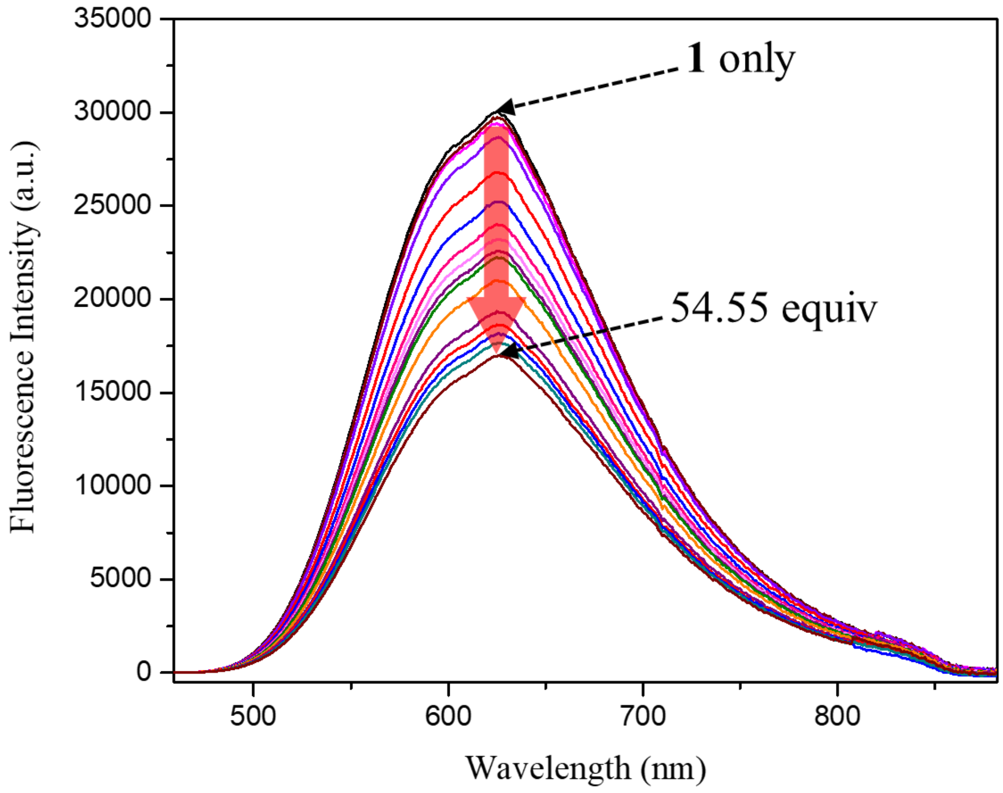

Figure S17. Fluorescence spectra of receptor **1** (100 µM) recorded during titrations with TBACl in CH_3CN. The excitation wavelength (λ_{ex}) was 448nm.

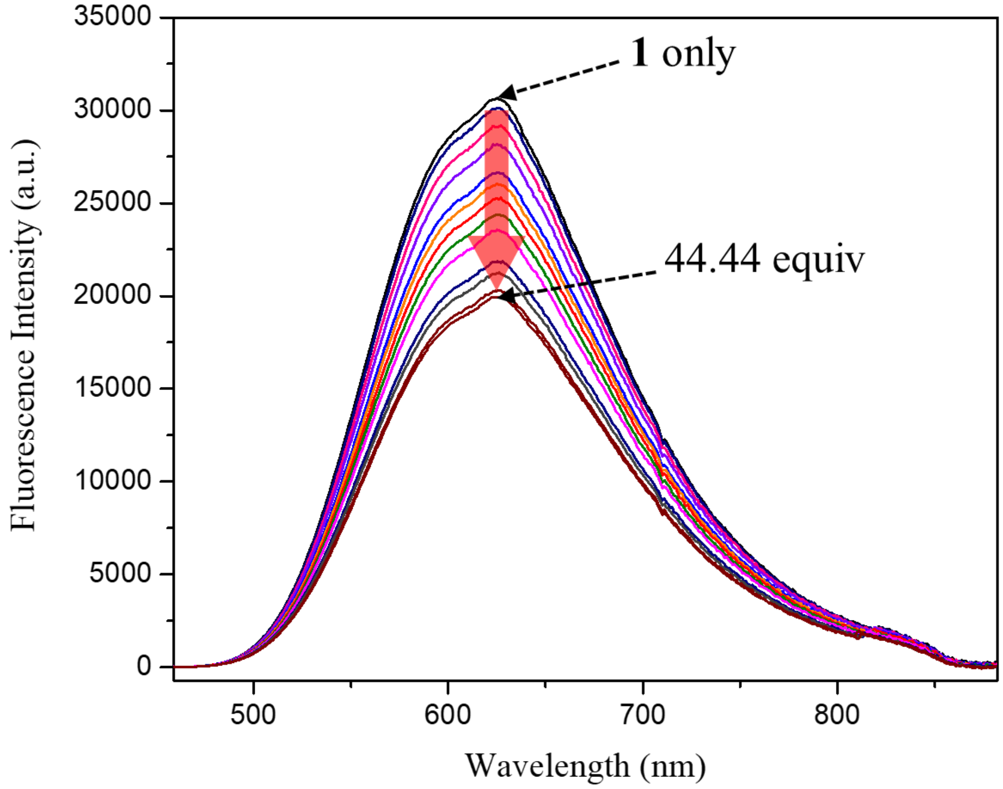

Figure S18. Fluorescence spectra of receptor **1** (100 μM) recorded during titrations with TBABr in CH_3CN. The excitation wavelength (λ_{ex}) was 448nm.

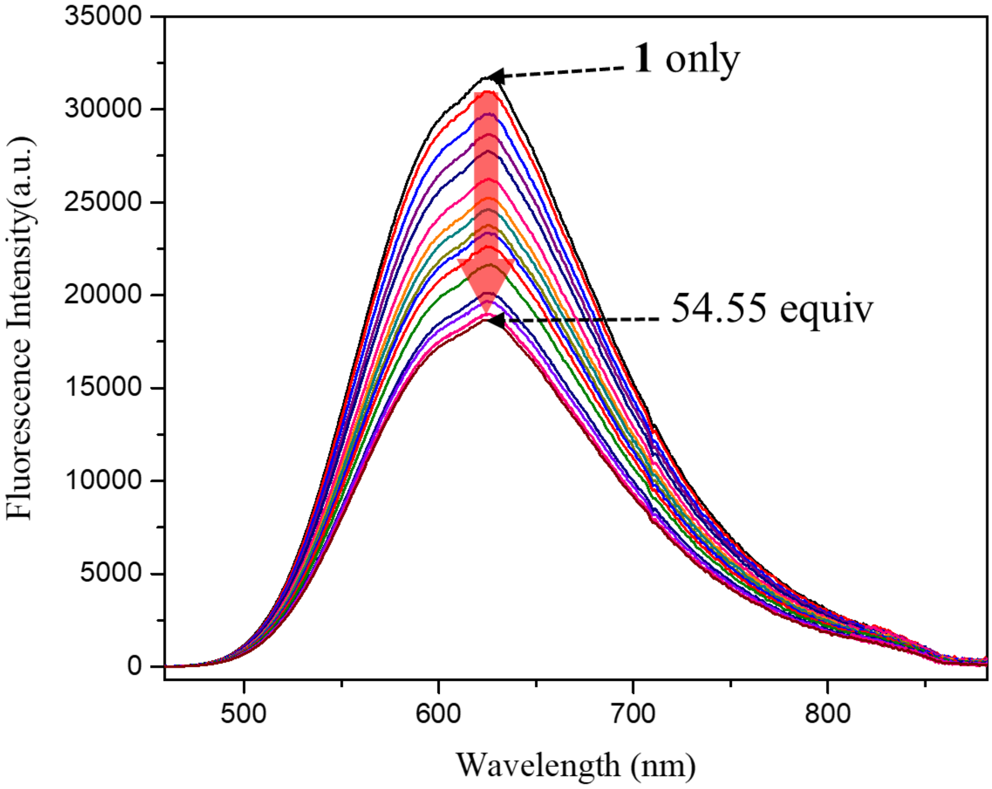

Figure S19. Fluorescence spectra of receptor **1** (100 μM) recorded during titrations with TBAI in CH_3CN. The excitation wavelength (λ_{ex}) was 448nm.

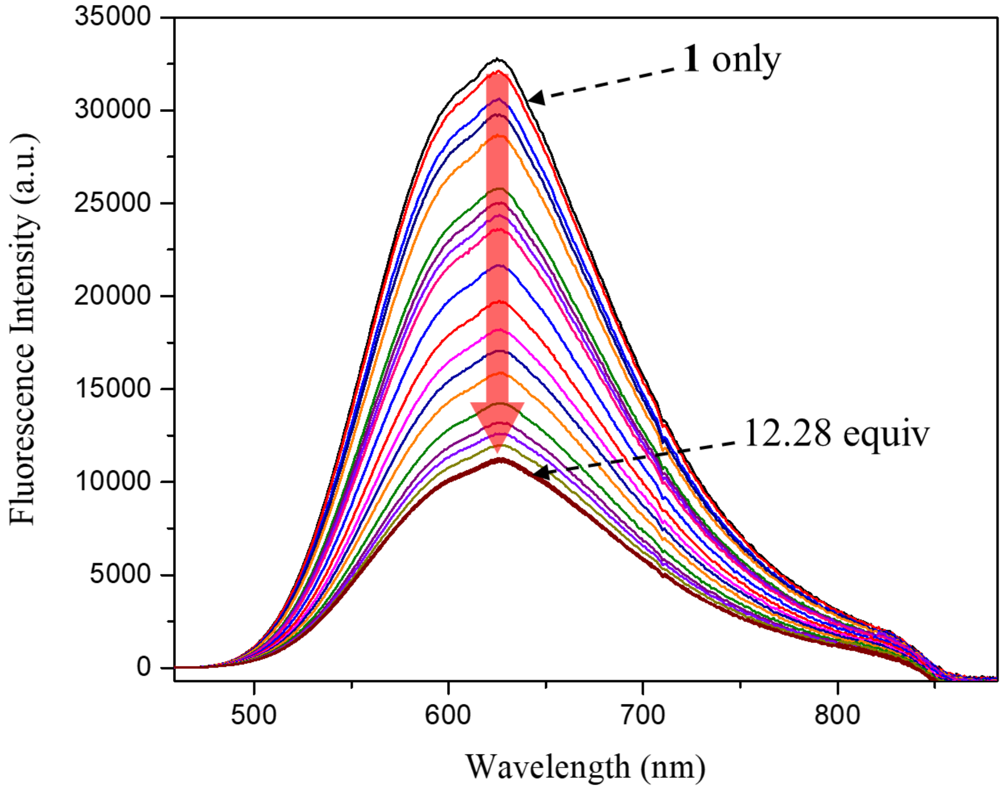

Figure S20. Fluorescence spectra of receptor **1** (100 μM) recorded during titrations with $TBAHSO_4$ in CH_3CN. The excitation wavelength (λ_{ex}) was 448nm.

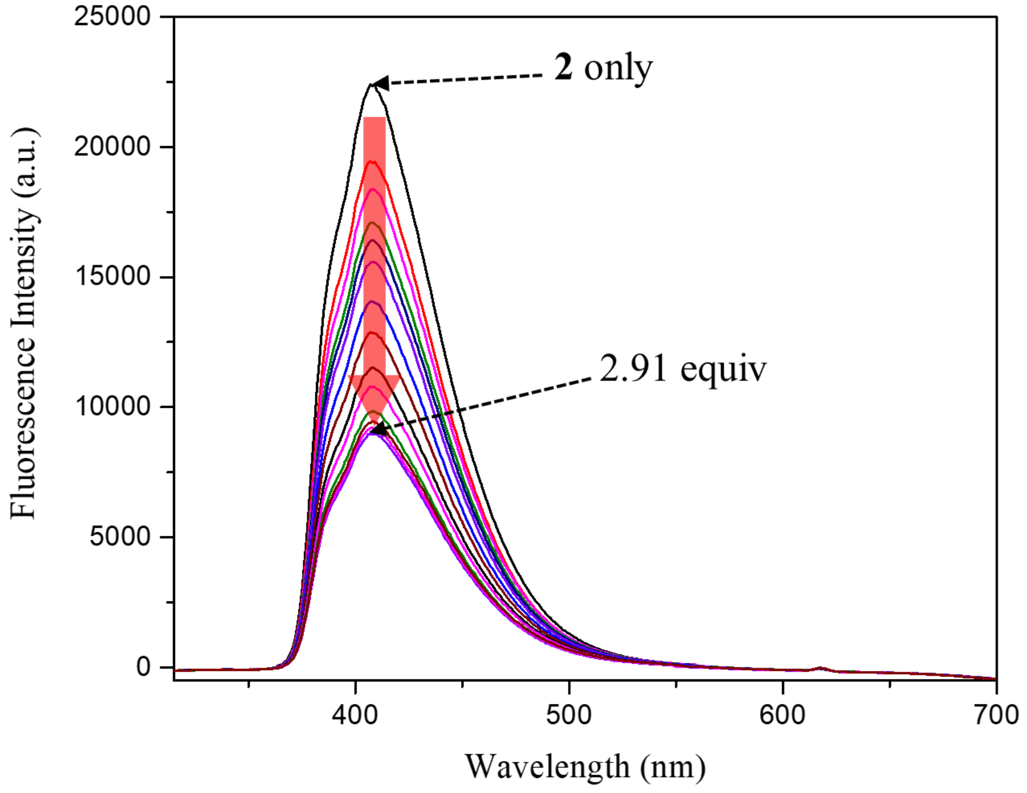

Figure S21. Fluorescence spectra of receptor **2** (15 μM) recorded during titrations with TBAF in CH_3CN. The excitation wavelength (λ_{ex}) was 307nm.

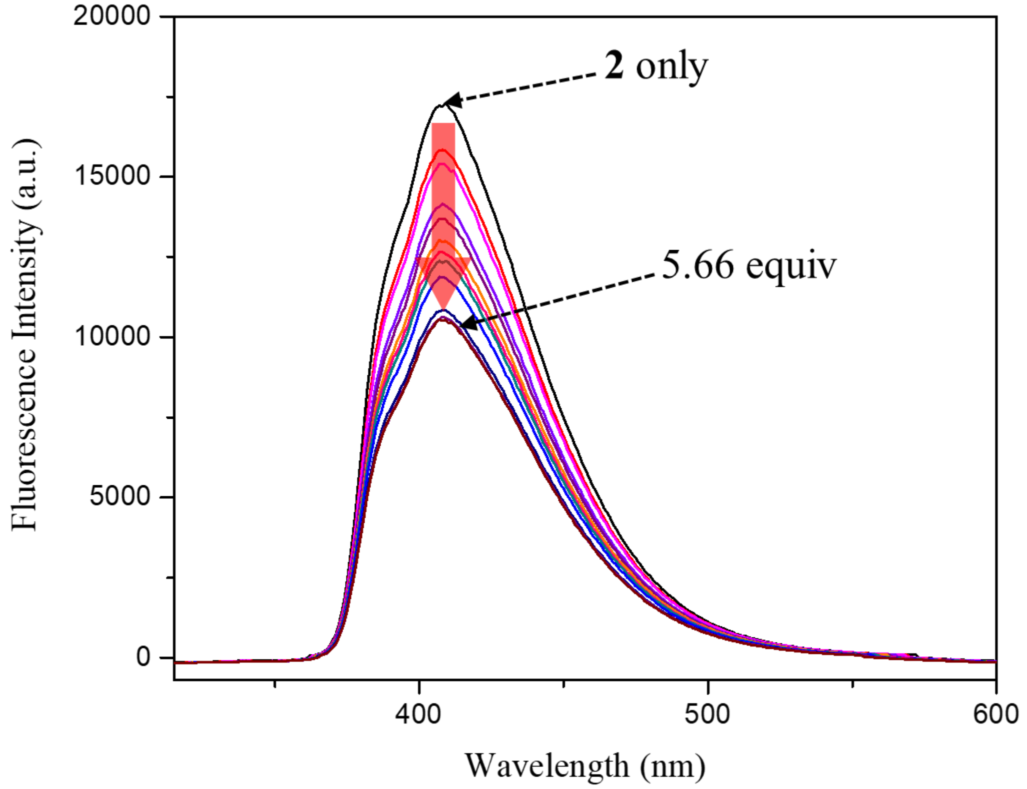

Figure S22. Fluorescence spectra of receptor **2** (15 μM) recorded during titrations with TBACl in CH_3CN. The excitation wavelength (λ_{ex}) was 307nm.

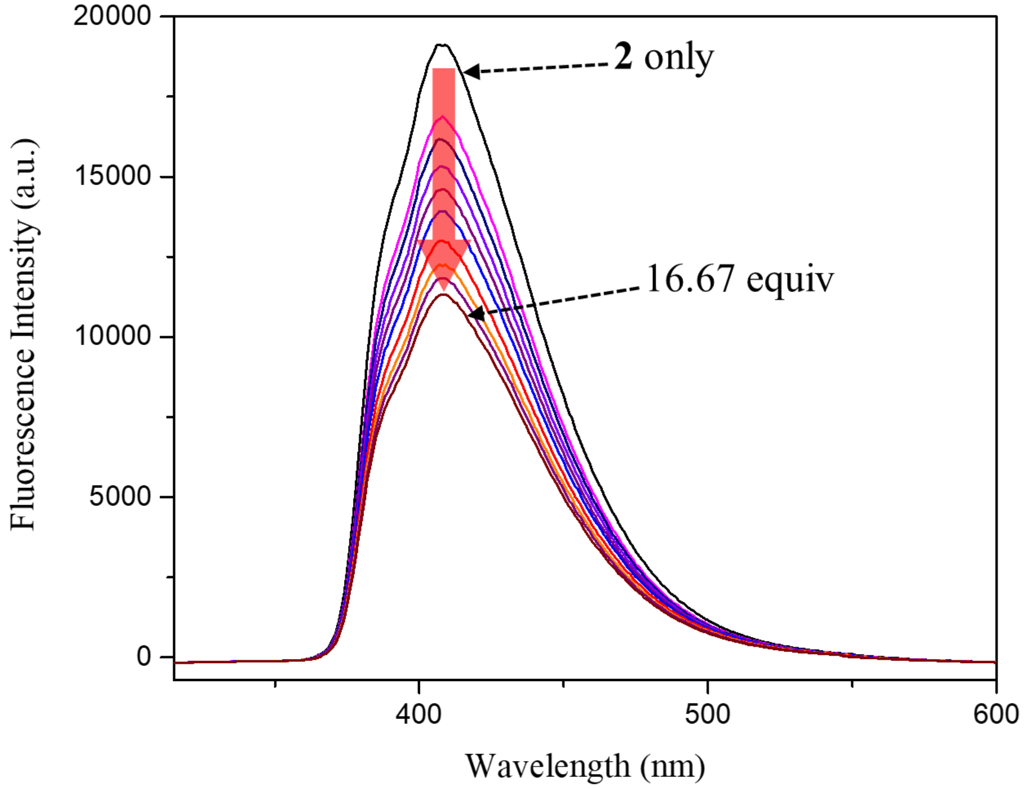

Figure S23. Fluorescence spectra of receptor **2** (15 μM) recorded during titrations with TBABr in CH_3CN. The excitation wavelength (λ_{ex}) was 307nm.

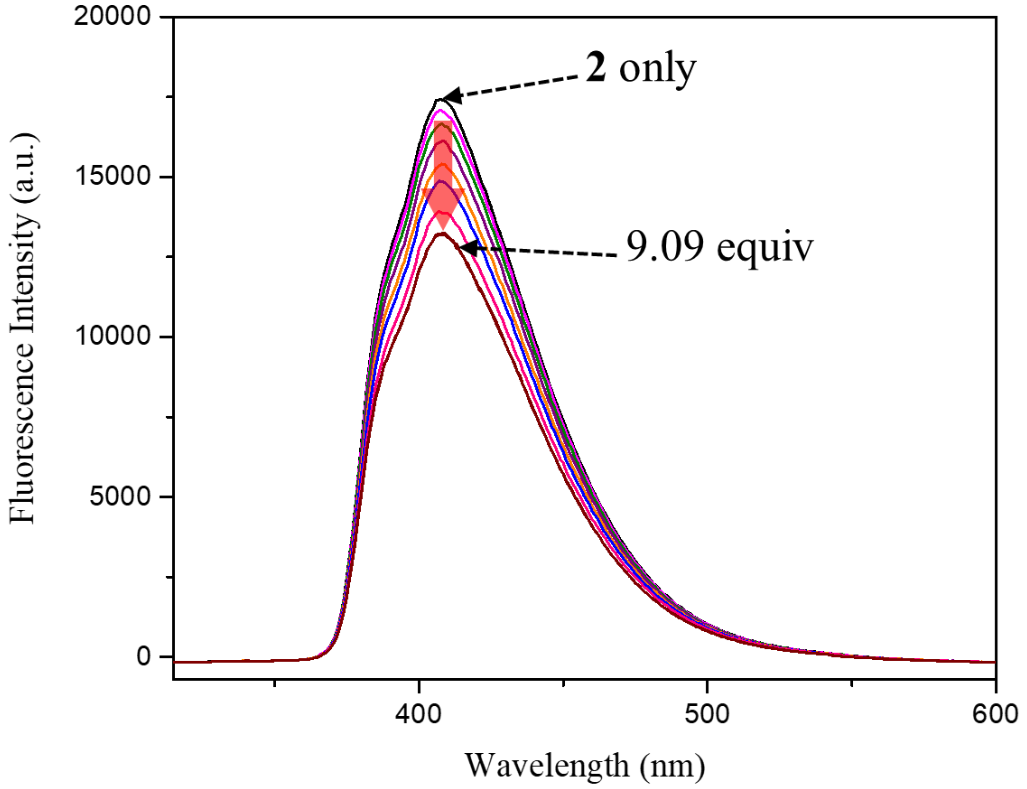

Figure S24. Fluorescence spectra of receptor **2** (15 µM) recorded during titrations with TBAI in CH_3CN. The excitation wavelength (λ_{ex}) was 307nm.

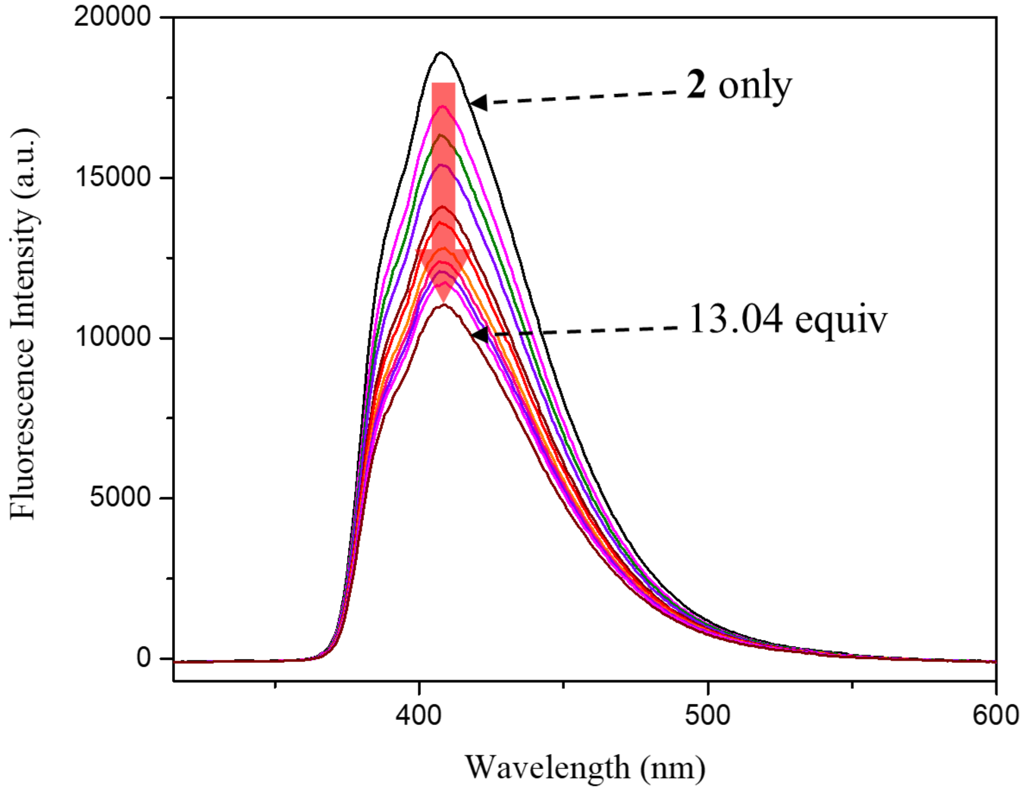

Figure S25. Fluorescence spectra of receptor **2** (15 µM) recorded during titrations with $TBAHSO_4$ in CH_3CN. The excitation wavelength (λ_{ex}) was 307nm.

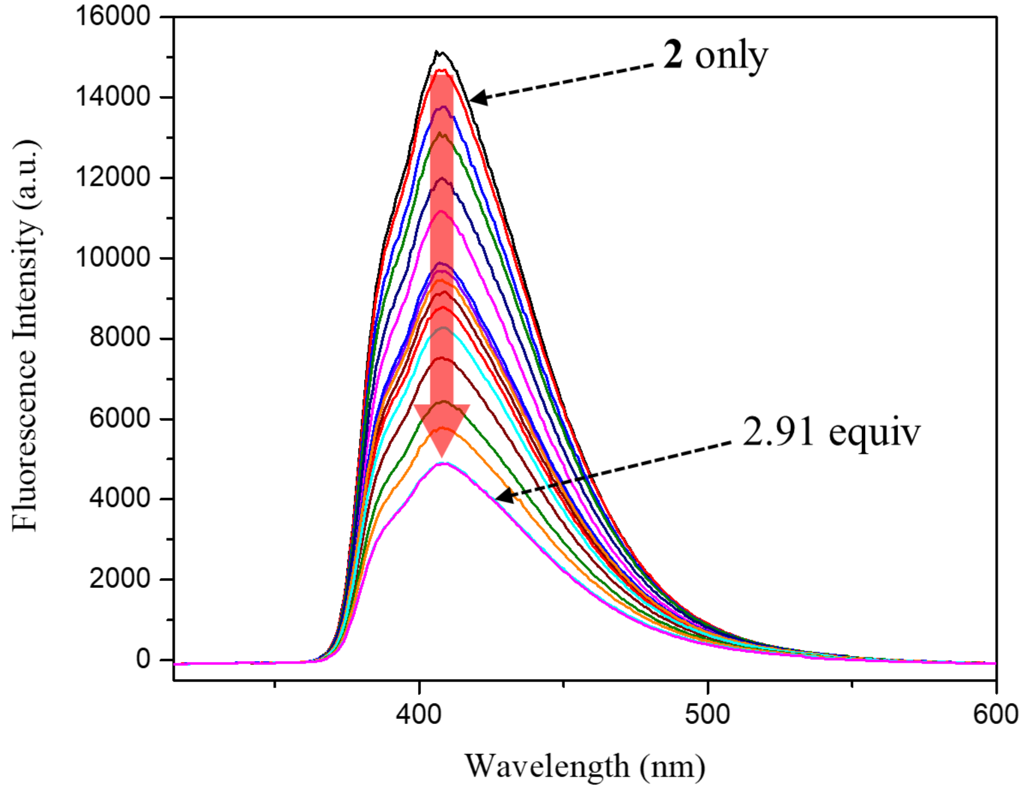

Figure S26. Fluorescence spectra of receptor **2** (15 µM) recorded during titrations with $TBAH_2PO_4$ in CH_3CN. The excitation wavelength (λ_{ex}) was 307nm.

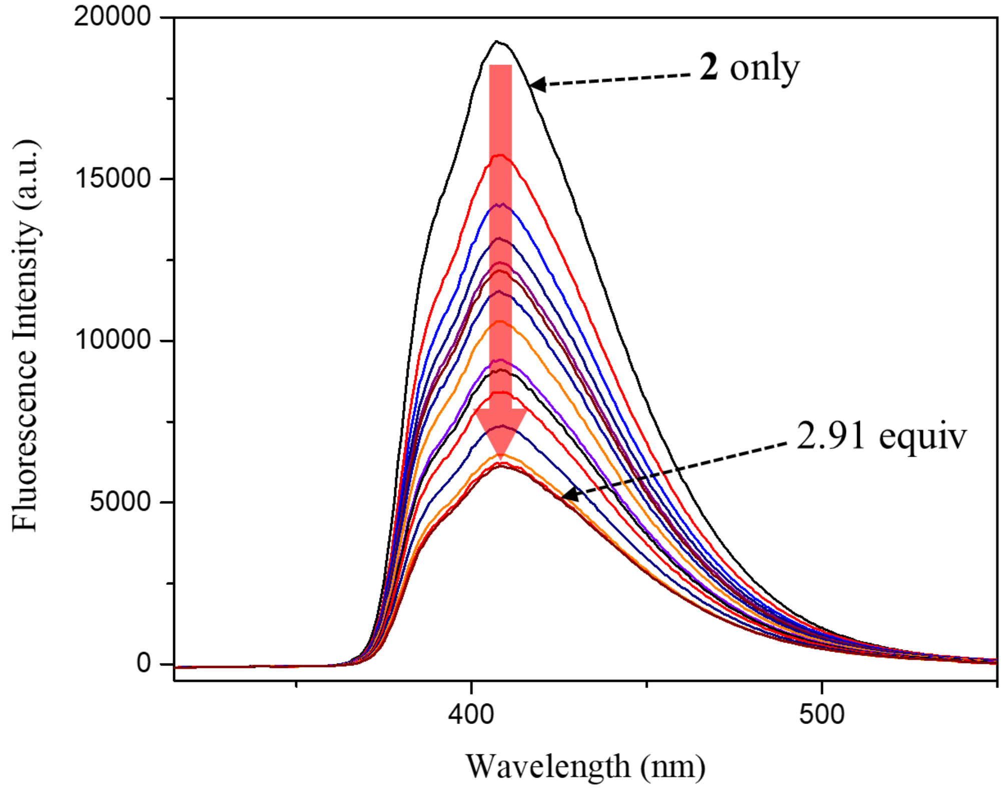

Figure S27. Fluorescence spectra of receptor **2** (15 μM) recorded during titrations with $(TBA)_3HP_2O_7$ in CH_3CN. The excitation wavelength (λ_{ex}) was 307nm.

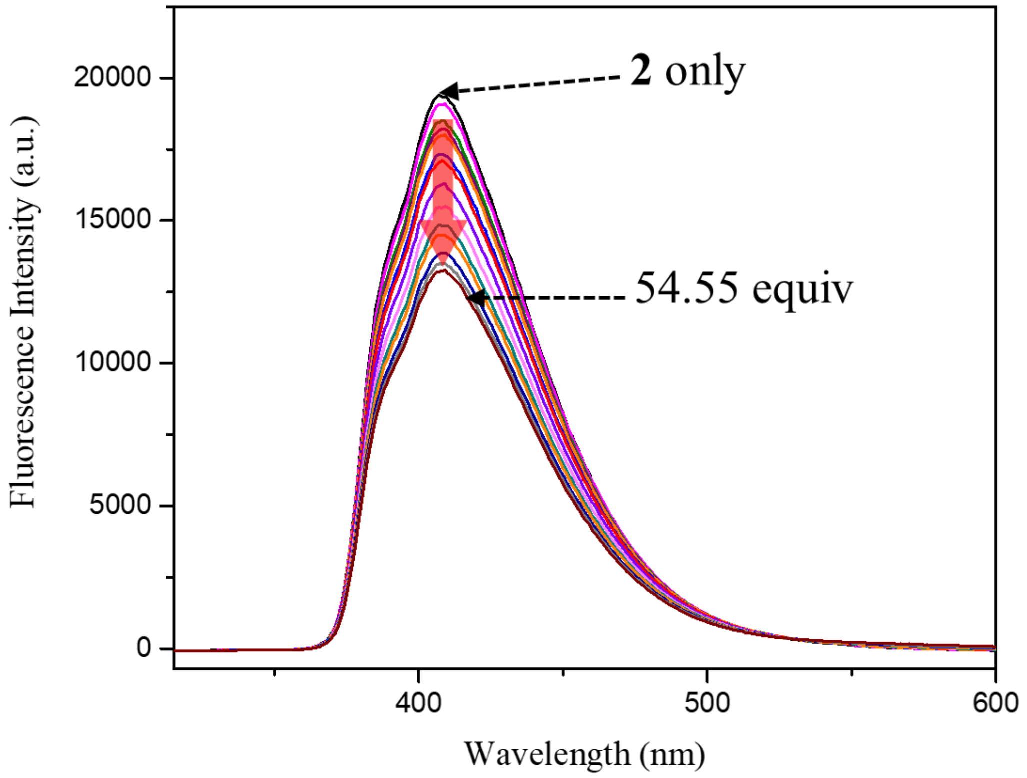

Figure S28. Fluorescence spectra of receptor **2** (15 μM) recorded during titrations with $TEAHCO_3$ in CH_3CN. The excitation wavelength (λ_{ex}) was = 307nm.

3. NMR spectra and HRMS data

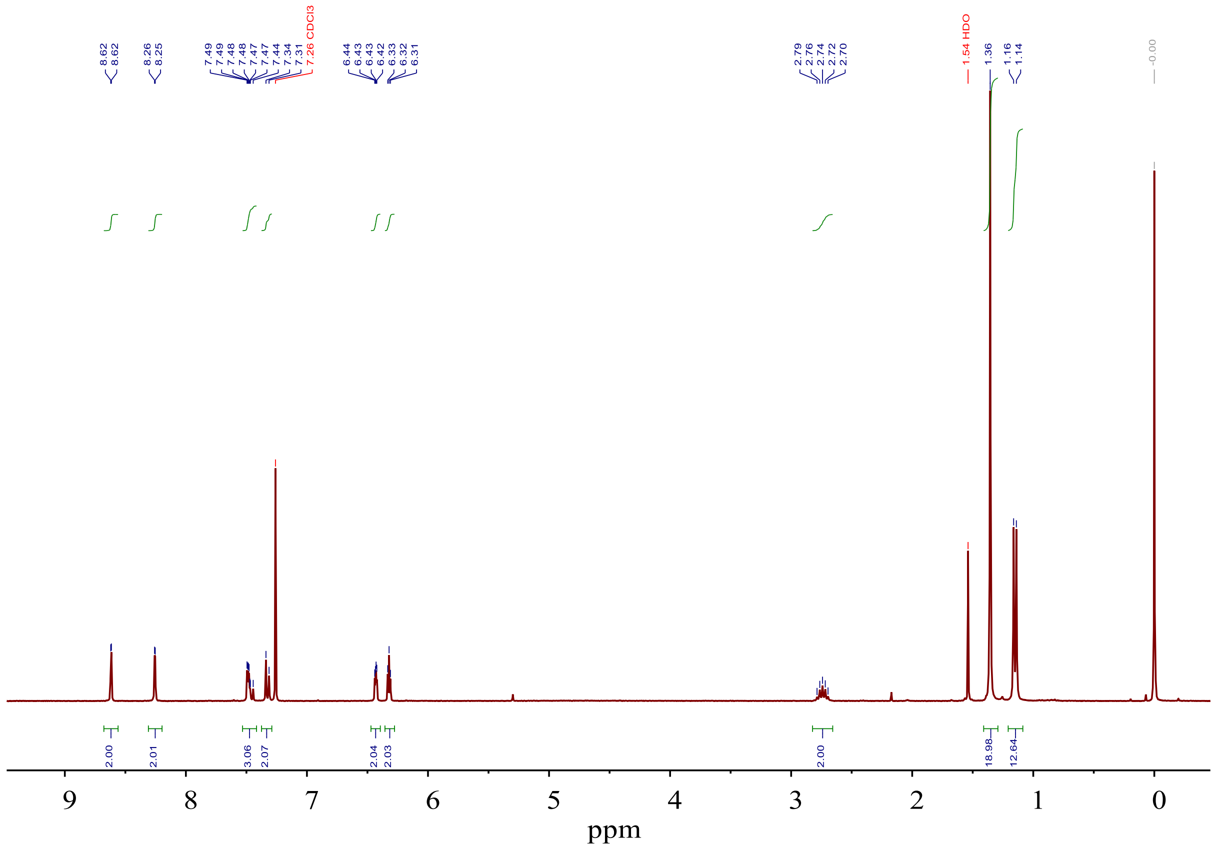

Figure S29. ^{1}H NMR spectrum of **6** recorded in $CDCl_3$.

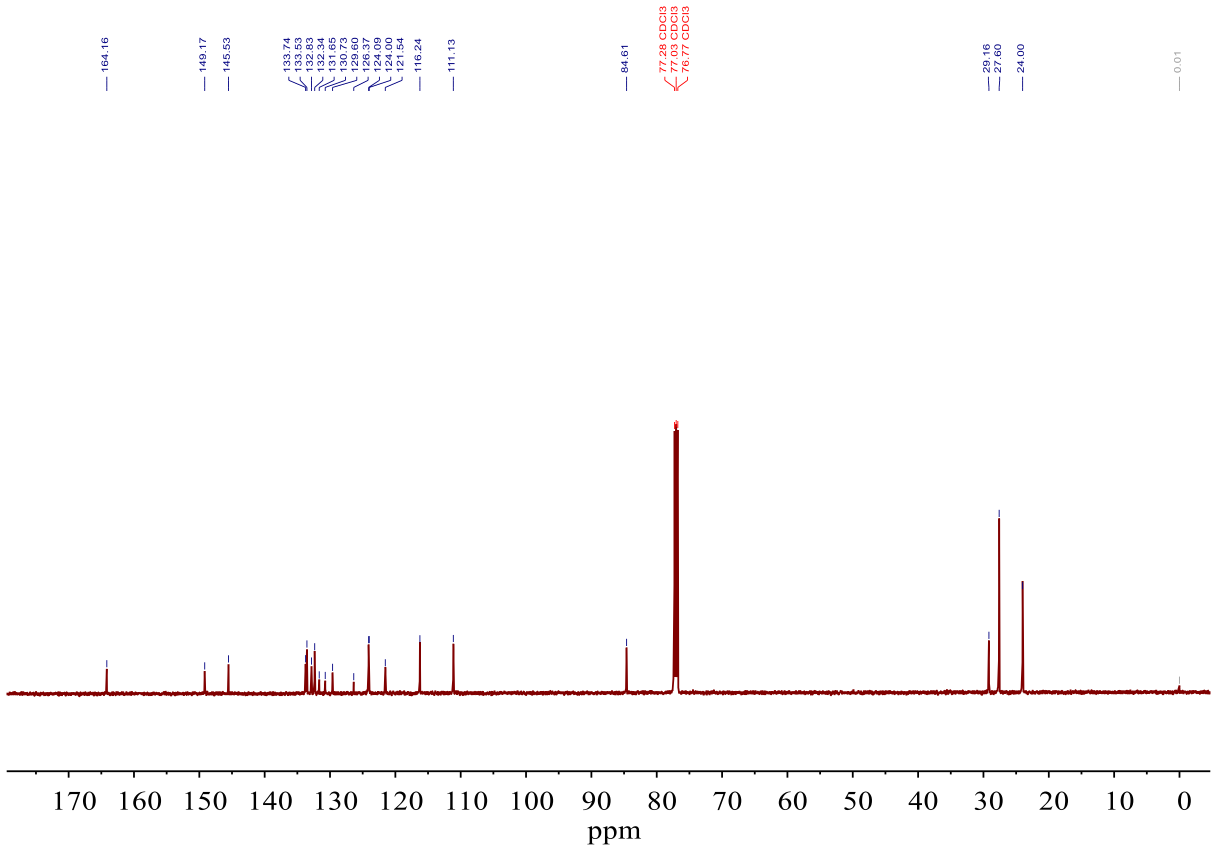

Figure S30. ^{13}C NMR spectrum of **6** recorded in $CDCl_3$.

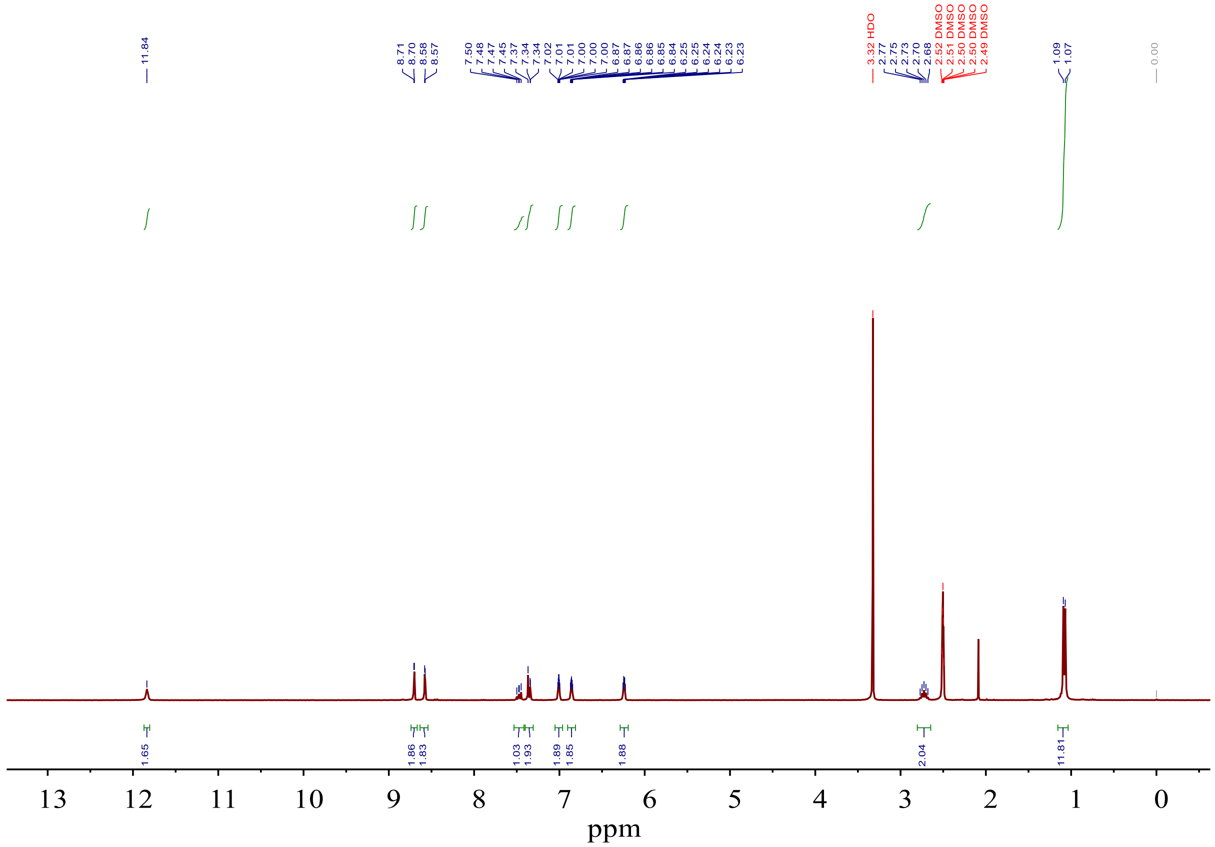

Figure S31. ^{1}H NMR spectrum of **1** recorded in DMSO-d_6.

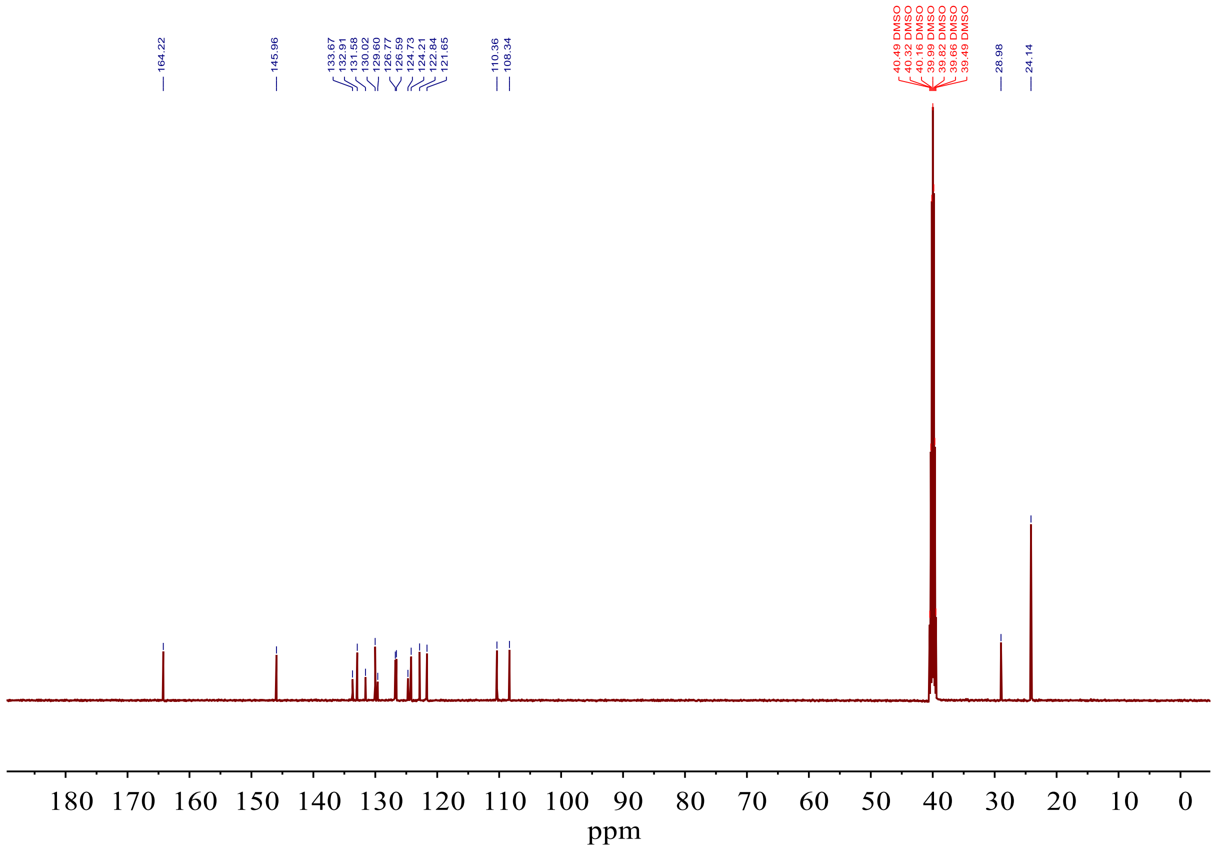

Figure 32. ^{13}C NMR spectrum of **1** recorded in DMSO-d_6.

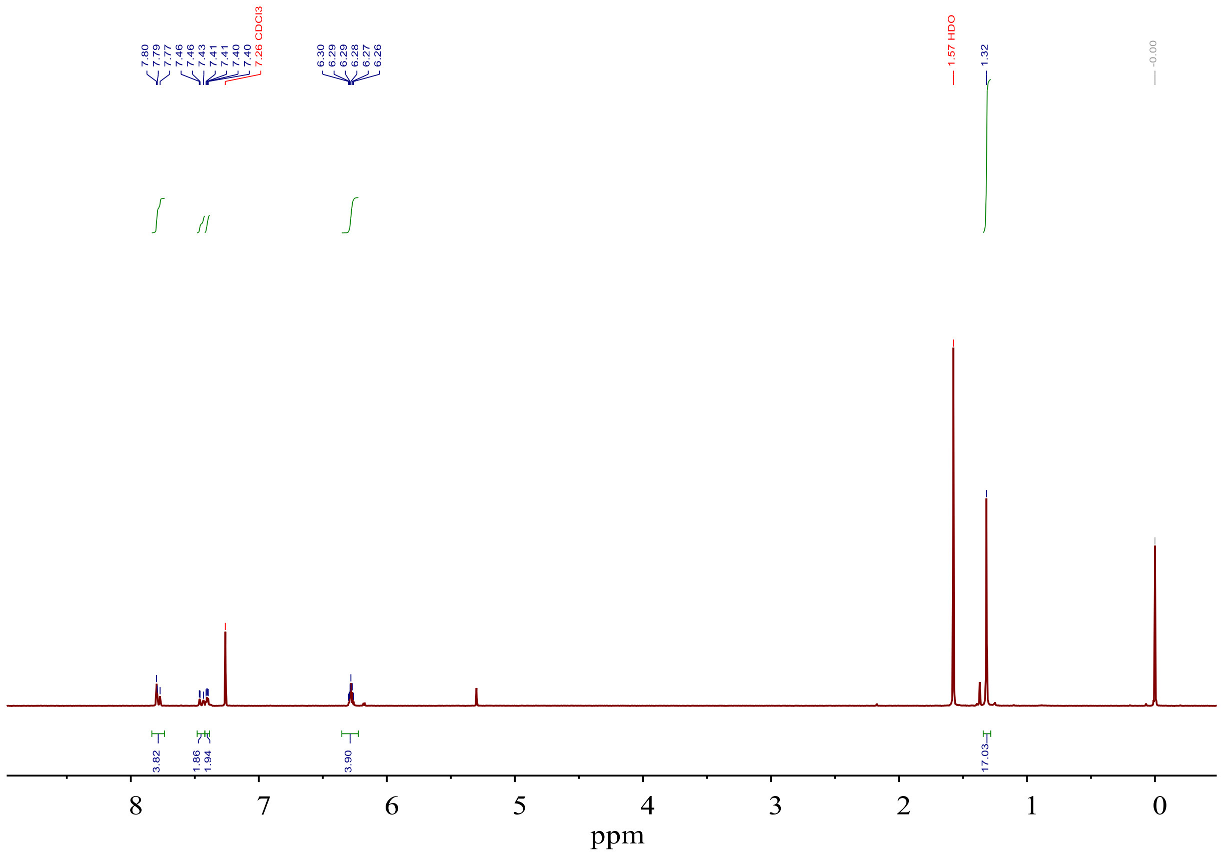

Figure 33. ^{1}H NMR spectrum of **7** recorded in $CDCl_3$.

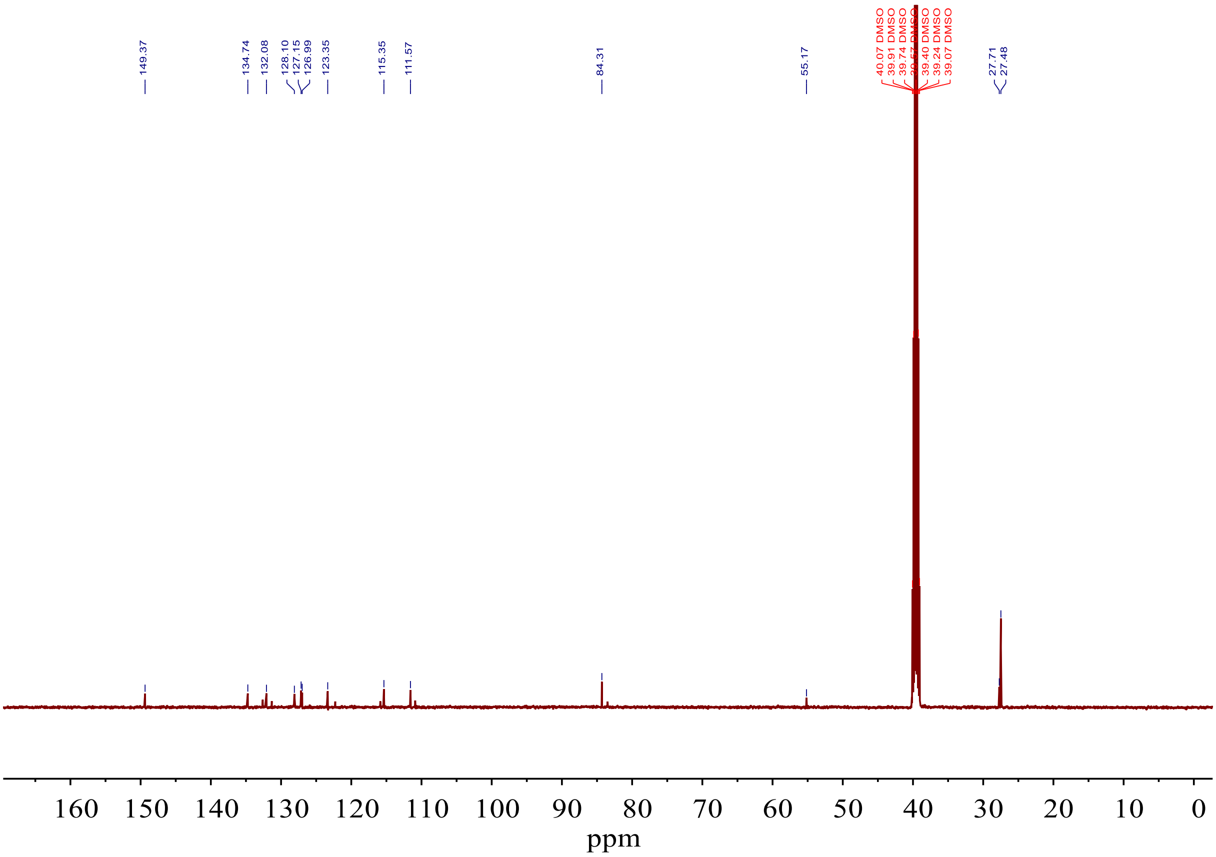

Figure 34. ^{13}C NMR spectrum of **7** recorded in DMSO-d_6.

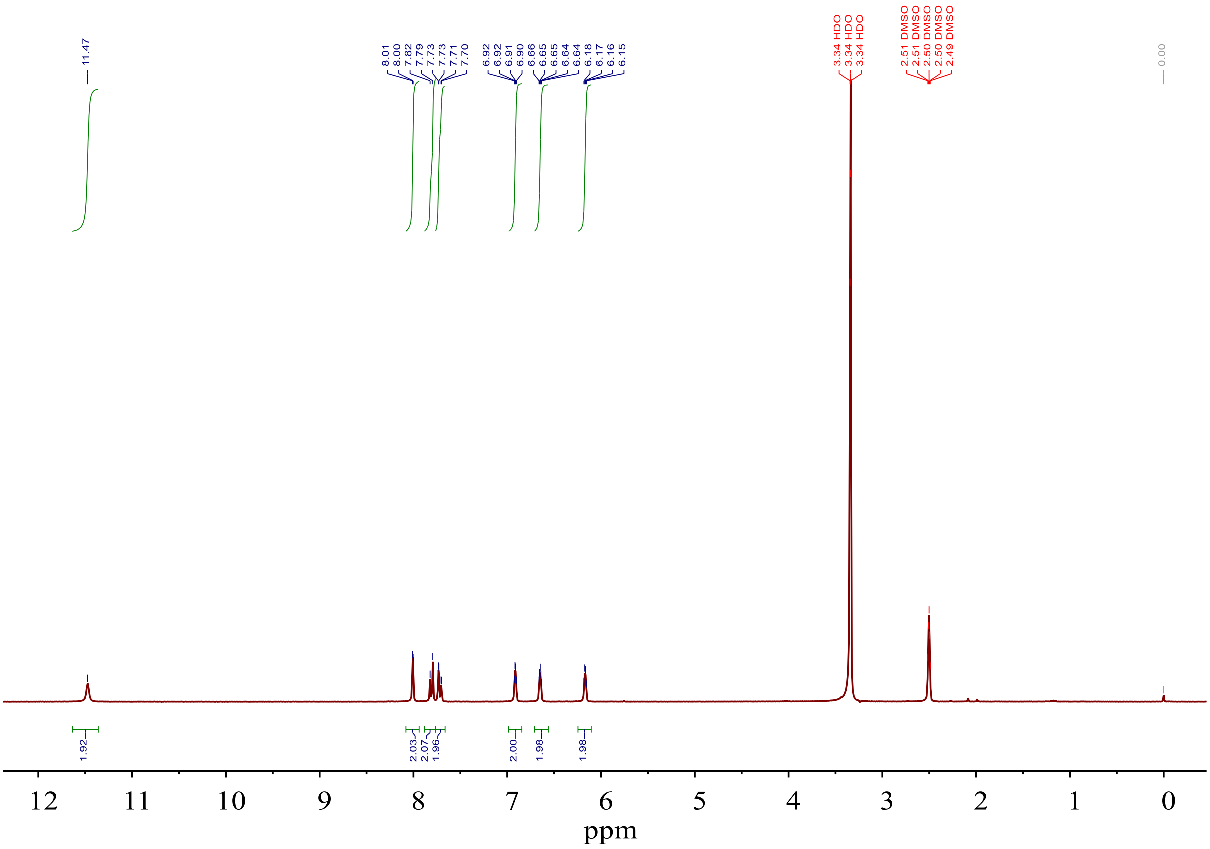

Figure S35. ^{1}H NMR spectrum of **2** recorded in DMSO-d6.

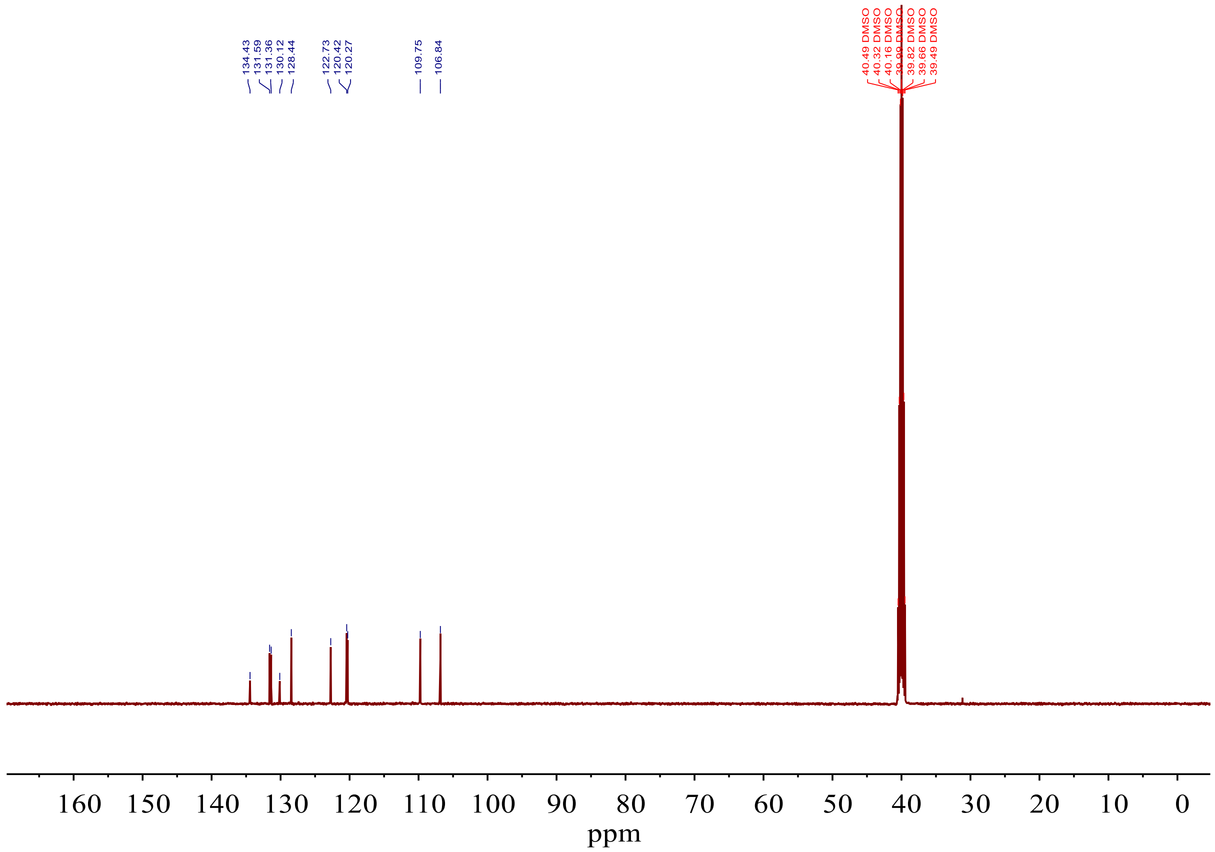

Figure S36. ^{13}C NMR spectrum of **2** recorded in DMSO-d$_6$.

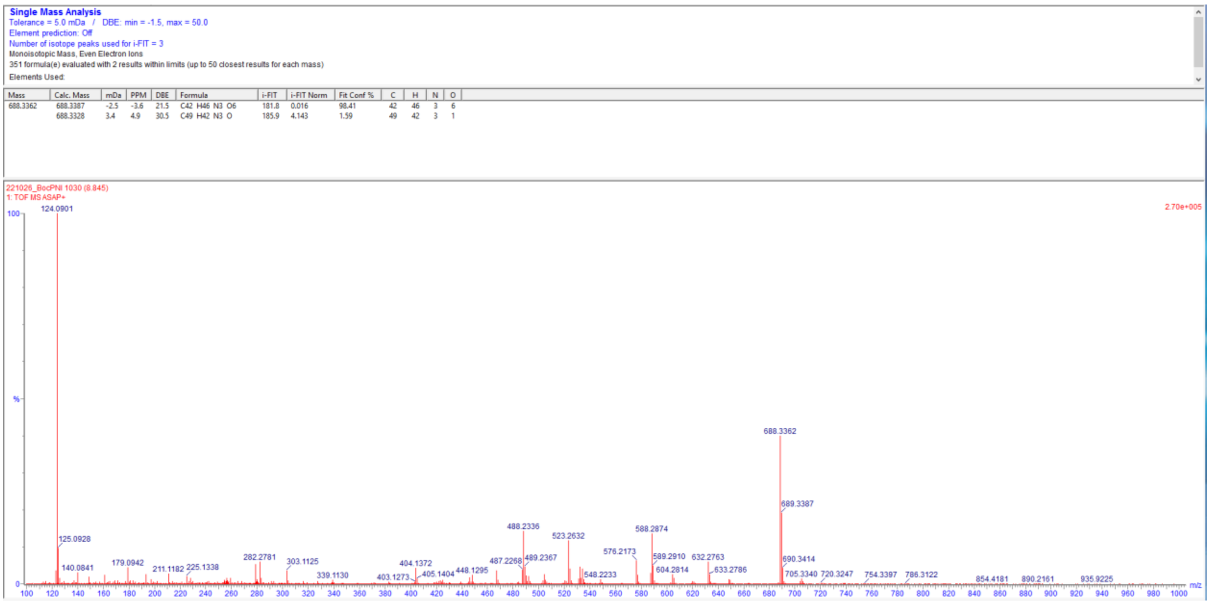

Figure S37. Partial Q-TOF HR MASS spectrum of **6**.

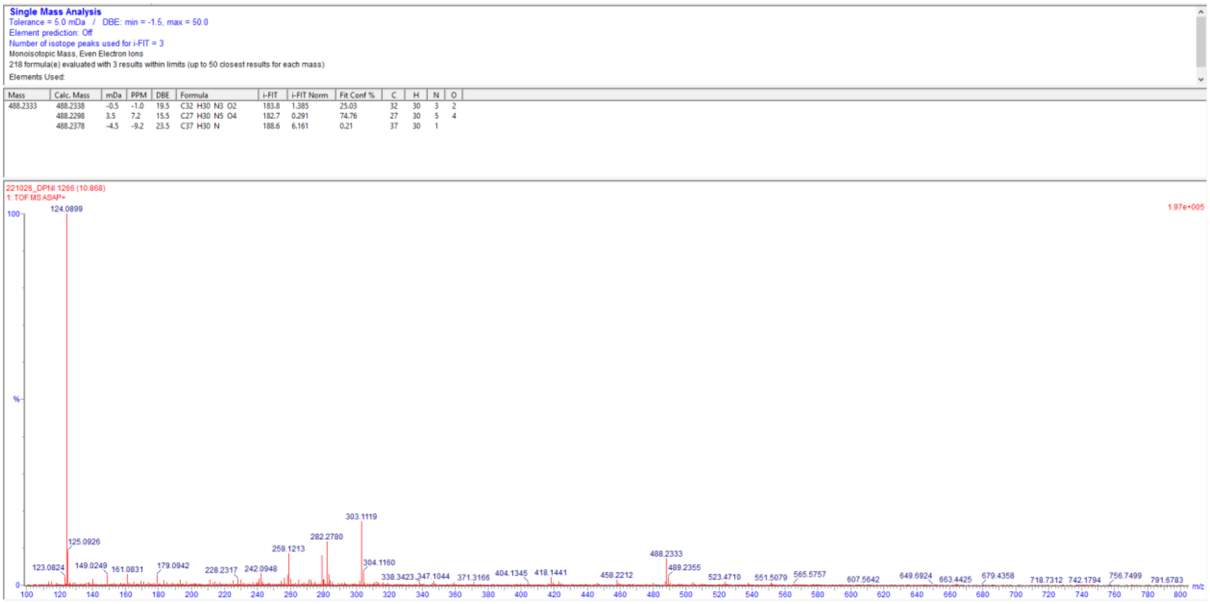

Figure S38. Partial Q-TOF HR MASS spectrum of **1**.

Single Mass Analysis
Tolerance = 5.0 mDa / DBE: min = -1.5, max = 50.0
Element prediction: Off
Number of isotope peaks used for i-FIT = 3
Monoisotopic Mass, Odd and Even Electron Ions
204 formula(e) evaluated with 4 results within limits (up to 50 closest results for each mass)
Elements Used:

Mass	Calc. Mass	mDa	PPM	DBE	Formula	i-FIT	i-FIT Norm	Fit Conf %	C	H	N	O
458.2200	458.2206	-0.6	-1.3	15.0	C28 H30 N2 O4	125.5	0.854	42.57	28	30	2	4
	458.2192	0.8	1.7	15.5	C26 H28 N5 O3	126.7	2.058	12.78	26	28	5	3
	458.2232	-3.2	-7.0	19.5	C31 H28 N3 O	125.4	0.818	44.12	31	28	3	1
	458.2246	-4.6	-10.0	19.0	C33 H30 O2	129.8	5.233	0.53	33	30		2

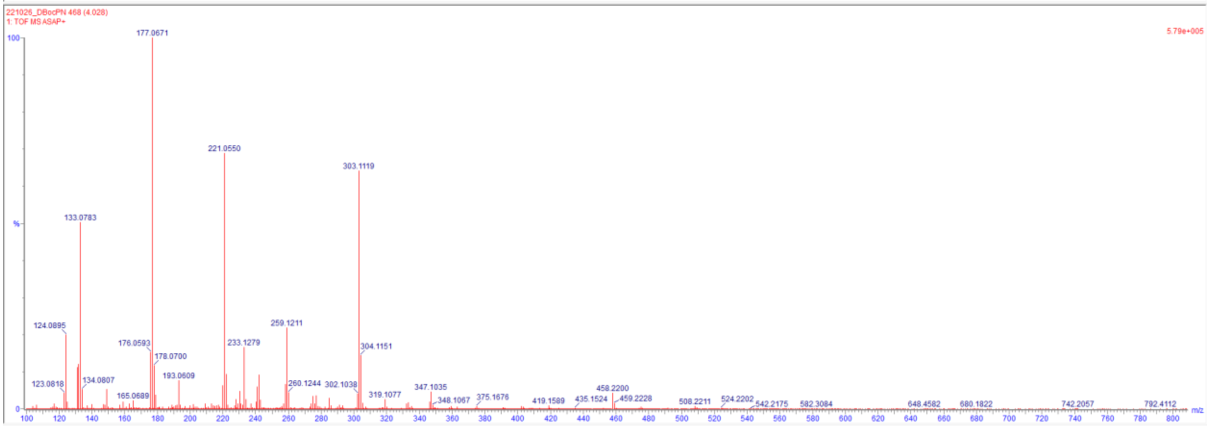

Figure S39. Partial Q-TOF HR MASS spectrum of **7**.

Tolerance = 5.0 mDa / DBE: min = -1.5, max = 50.0
Element prediction: Off
Number of isotope peaks used for i-FIT = 3
Monoisotopic Mass, Even Electron Ions
61 formula(e) evaluated with 2 results within limits (up to 50 closest results for each mass)
Elements Used:

Mass	RA	Calc. Mass	mDa	PPM	DBE	Formula	i-FIT	i-FIT Norm	Fit Conf %	C	H	N
259.1212	100.00	259.1235	-2.3	-8.9	12.5	C18 H15 N2	1211.6	0.000	100.00	18	15	2
		259.1168	4.4	17.0	9.5	C9 H11 N10	1221.6	9.976	0.00	9	11	10

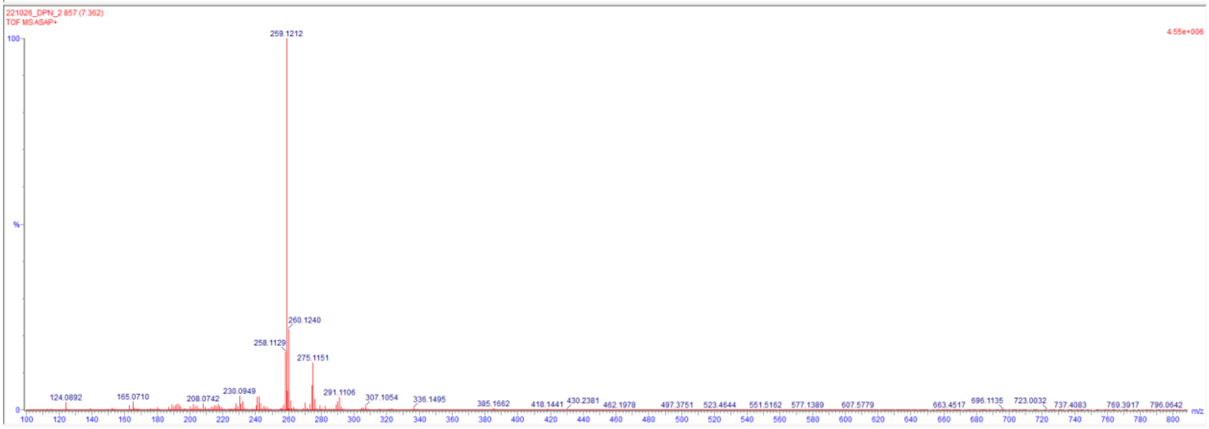

Figure S40. Partial Q-TOF HR MASS spectrum of **2**.

4. References

1. Xue, J.Y.; Nakanishi, W.; Tanimoto, D.; Hara, D.; Nakamura, Y.; Isobe, H. Convergent synthesis of hexameric naphthylene macrocycles with dicarboxylic imide appendages. *Tetrahedron Lett.* **2013**, *54, 4963−4965.*
2. Frigoli, M.; Moustrou, C.; Samat, A.; Guglielmetti, R. Synthesis of New thiophene-substituted 3,3-diphenyl-3H-naphtho[2,1-b]pyrans by cross-coupling reactions, precursors of photomodulated materials. *Eur. J. Org. Chem*. **2003**, *15*, 2799−2812.
3. Salman, H.; Abraham, Y.; Tal, S.; Meltzman.; Kapon, M.; Tessler, N.; Speiser, S.; Eichen, Y. 1,3-Di(2-pyrrolyl)azulene: An efficient luminescent probe for fluoride. *Eur. J. Org. Chem.* **2005**, *2005*, 2207–2212
4. Weidauer, M.; Irran, E.; Enthaler, S. Synthesis and structural characterization of a trispyrrole iron(II) complex $K(dme)_4[tpa^{Mes}Fe]$ and application in nitrous oxide dependent coupling reactions. *Inorg. Chem. Commun.* **2015**, *54*, 1–4.
5. Conformational searches performed with PCModel, Version 9.3; Serena Software: Bloomington, IN, 2012.
6. Halgren, T.A. Merck molecular force field. I. Basis, form, scope, parameterization, and performance of MMFF94. *J. Comp. Chem.* **1996**, *17*, 490-519.

5. Molecular mechanics computation data

```
HEADER    H2PO4- anion
COMPND    MMFF94 geometry optimized
AUTHOR    GENERATED BY PCMODEL V 9.30
HETATM    1  P   UNK   1        -0.010   0.008   0.214  1.00  0.00
HETATM    2  O   UNK   1         0.020  -1.328   0.883  1.00  0.00
HETATM    3  O   UNK   1        -0.051   1.328   0.912  1.00  0.00
HETATM    4  O   UNK   1        -1.198   0.042  -0.850  1.00  0.00
HETATM    5  O   UNK   1         1.196  -0.003  -0.830  1.00  0.00
HETATM    6  H   UNK   1        -1.389   1.000  -0.912  1.00  0.00
HETATM    7  H   UNK   1         1.389  -0.960  -0.910  1.00  0.00
CONECT    1    2    3    3    4    5
CONECT    2    1
CONECT    3    1    1
CONECT    4    1    6
CONECT    5    1    7
CONECT    6    4
CONECT    7    5
MASTER        0    0    0    0    0    0    0    0    7    0    7    0
END


HEADER    HP2O73- anion
COMPND    MMFF94 geometry optimized
AUTHOR    GENERATED BY PCMODEL V 9.30
HETATM    1  O   UNK   1        -1.753  -0.778   0.053  1.00  0.00
HETATM    2  P   UNK   1        -1.410   0.796  -0.061  1.00  0.00
HETATM    3  O   UNK   1         0.159   0.723  -0.416  1.00  0.00
HETATM    4  P   UNK   1         1.298  -0.258   0.187  1.00  0.00
HETATM    5  O   UNK   1         2.058   0.537   1.234  1.00  0.00
HETATM    6  O   UNK   1        -2.167   1.314  -1.277  1.00  0.00
HETATM    7  O   UNK   1        -1.708   1.442   1.277  1.00  0.00
HETATM    8  O   UNK   1         0.510  -1.441   0.744  1.00  0.00
HETATM    9  O   UNK   1         2.166  -0.635  -1.006  1.00  0.00
HETATM   10  H   UNK   1        -0.910  -1.166   0.473  1.00  0.00
CONECT    1    2   10
CONECT    2    1    3    6    6    7
CONECT    3    2    4
CONECT    4    3    5    8    8    9
CONECT    5    4
CONECT    6    2    2
CONECT    7    2
CONECT    8    4    4
CONECT    9    4
CONECT   10    1
MASTER        0    0    0    0    0    0    0    0   10    0   10    0
END


HEADER    Naphthalene host
COMPND    MMFF94 geometry optimized
AUTHOR    GENERATED BY PCMODEL V 9.30
HETATM    1  C   UNK   1         4.622  -0.424  -0.673  1.00  0.00
HETATM    2  C   UNK   1        -0.013   1.584   0.915  1.00  0.00
HETATM    3  C   UNK   1        -0.133   0.201   0.662  1.00  0.00
HETATM    4  C   UNK   1         1.024  -0.550   0.384  1.00  0.00
HETATM    5  C   UNK   1         2.291   0.049   0.349  1.00  0.00
HETATM    6  C   UNK   1         2.392   1.422   0.590  1.00  0.00
HETATM    7  C   UNK   1         1.254   2.182   0.873  1.00  0.00
HETATM    8  N   UNK   1         3.689  -2.012   0.555  1.00  0.00
HETATM    9  C   UNK   1         3.490  -0.739   0.062  1.00  0.00
```

```
HETATM   10  C   UNK   1        4.898  -2.500   0.136  1.00  0.00
HETATM   11  C   UNK   1       -1.407  -0.396   0.694  1.00  0.00
HETATM   12  C   UNK   1       -2.558   0.354   0.976  1.00  0.00
HETATM   13  C   UNK   1       -2.421   1.721   1.236  1.00  0.00
HETATM   14  C   UNK   1       -1.164   2.331   1.203  1.00  0.00
HETATM   15  C   UNK   1        5.501  -1.533  -0.633  1.00  0.00
HETATM   16  C   UNK   1       -3.880  -0.273   1.004  1.00  0.00
HETATM   17  C   UNK   1       -4.965  -0.052   1.837  1.00  0.00
HETATM   18  C   UNK   1       -6.014  -0.914   1.437  1.00  0.00
HETATM   19  N   UNK   1       -4.272  -1.239   0.102  1.00  0.00
HETATM   20  C   UNK   1       -5.558  -1.635   0.360  1.00  0.00
HETATM   21  H   UNK   1        3.044  -2.501   1.160  1.00  0.00
HETATM   22  H   UNK   1       -3.699  -1.585  -0.656  1.00  0.00
HETATM   23  H   UNK   1        4.792   0.509  -1.196  1.00  0.00
HETATM   24  H   UNK   1        0.927  -1.613   0.172  1.00  0.00
HETATM   25  H   UNK   1        3.363   1.914   0.573  1.00  0.00
HETATM   26  H   UNK   1        1.368   3.247   1.064  1.00  0.00
HETATM   27  H   UNK   1        5.226  -3.490   0.425  1.00  0.00
HETATM   28  H   UNK   1       -1.495  -1.466   0.517  1.00  0.00
HETATM   29  H   UNK   1       -3.296   2.329   1.454  1.00  0.00
HETATM   30  H   UNK   1       -1.094   3.398   1.402  1.00  0.00
HETATM   31  H   UNK   1        6.469  -1.613  -1.110  1.00  0.00
HETATM   32  H   UNK   1       -4.993   0.652   2.659  1.00  0.00
HETATM   33  H   UNK   1       -6.996  -0.994   1.884  1.00  0.00
HETATM   34  H   UNK   1       -6.037  -2.391  -0.248  1.00  0.00
CONECT    1    9    9   15   23
CONECT    2    3    3    7   14
CONECT    3    2    2    4   11
CONECT    4    3    5    5   24
CONECT    5    4    4    6    9
CONECT    6    5    7    7   25
CONECT    7    2    6    6   26
CONECT    8    9   10   21
CONECT    9    1    1    5    8
CONECT   10    8   15   15   27
CONECT   11    3   12   12   28
CONECT   12   11   11   13   16
CONECT   13   12   14   14   29
CONECT   14    2   13   13   30
CONECT   15    1   10   10   31
CONECT   16   12   17   17   19
CONECT   17   16   16   18   32
CONECT   18   17   20   20   33
CONECT   19   16   20   22
CONECT   20   18   18   19   34
CONECT   21    8
CONECT   22   19
CONECT   23    1
CONECT   24    4
CONECT   25    6
CONECT   26    7
CONECT   27   10
CONECT   28   11
CONECT   29   13
CONECT   30   14
CONECT   31   15
CONECT   32   17
CONECT   33   18
CONECT   34   20
MASTER        0    0    0    0    0    0    0    0   34    0   34    0
END
```

```
HEADER    Naphthalene host + flouride guest
COMPND    MMFF94 geometry optimized
AUTHOR    GENERATED BY PCMODEL V 9.30
HETATM    1  C   UNK   1         4.629   0.563  -1.689  1.00  0.00
HETATM    2  C   UNK   1        -0.497   1.646  -0.765  1.00  0.00
HETATM    3  C   UNK   1        -0.243   0.296  -0.459  1.00  0.00
HETATM    4  C   UNK   1         1.065  -0.205  -0.566  1.00  0.00
HETATM    5  C   UNK   1         2.140   0.607  -0.951  1.00  0.00
HETATM    6  C   UNK   1         1.871   1.947  -1.256  1.00  0.00
HETATM    7  C   UNK   1         0.571   2.462  -1.168  1.00  0.00
HETATM    8  N   UNK   1         3.914  -1.088  -0.390  1.00  0.00
HETATM    9  C   UNK   1         3.509   0.075  -1.029  1.00  0.00
HETATM   10  C   UNK   1         5.248  -1.317  -0.644  1.00  0.00
HETATM   11  C   UNK   1        -1.297  -0.532  -0.039  1.00  0.00
HETATM   12  C   UNK   1        -2.604  -0.045   0.096  1.00  0.00
HETATM   13  C   UNK   1        -2.846   1.297  -0.223  1.00  0.00
HETATM   14  C   UNK   1        -1.807   2.134  -0.649  1.00  0.00
HETATM   15  C   UNK   1         5.716  -0.309  -1.448  1.00  0.00
HETATM   16  C   UNK   1        -3.691  -0.910   0.570  1.00  0.00
HETATM   17  C   UNK   1        -5.061  -0.838   0.373  1.00  0.00
HETATM   18  C   UNK   1        -5.670  -1.915   1.061  1.00  0.00
HETATM   19  N   UNK   1        -3.475  -2.012   1.374  1.00  0.00
HETATM   20  C   UNK   1        -4.665  -2.624   1.673  1.00  0.00
HETATM   21  H   UNK   1         4.659   1.459  -2.294  1.00  0.00
HETATM   22  H   UNK   1         1.239  -1.254  -0.333  1.00  0.00
HETATM   23  H   UNK   1         2.676   2.614  -1.550  1.00  0.00
HETATM   24  H   UNK   1         0.404   3.509  -1.406  1.00  0.00
HETATM   25  H   UNK   1         3.327  -1.703   0.193  1.00  0.00
HETATM   26  H   UNK   1         5.721  -2.191  -0.217  1.00  0.00
HETATM   27  H   UNK   1        -1.069  -1.574   0.183  1.00  0.00
HETATM   28  H   UNK   1        -3.844   1.714  -0.125  1.00  0.00
HETATM   29  H   UNK   1        -2.028   3.173  -0.878  1.00  0.00
HETATM   30  H   UNK   1         6.726  -0.215  -1.820  1.00  0.00
HETATM   31  H   UNK   1        -5.572  -0.090  -0.219  1.00  0.00
HETATM   32  H   UNK   1        -6.726  -2.147   1.104  1.00  0.00
HETATM   33  H   UNK   1        -2.566  -2.315   1.704  1.00  0.00
HETATM   34  H   UNK   1        -4.685  -3.509   2.294  1.00  0.00
HETATM   35  F   UNK   1         2.452  -3.017   1.196  1.00  0.00
CONECT    1    9    9   15   21
CONECT    2    3    3    7   14
CONECT    3    2    2    4   11
CONECT    4    3    5    5   22
CONECT    5    4    4    6    9
CONECT    6    5    7    7   23
CONECT    7    2    6    6   24
CONECT    8    9   10   25
CONECT    9    1    1    5    8
CONECT   10    8   15   15   26
CONECT   11    3   12   12   27
CONECT   12   11   11   13   16
CONECT   13   12   14   14   28
CONECT   14    2   13   13   29
CONECT   15    1   10   10   30
CONECT   16   12   17   17   19
CONECT   17   16   16   18   31
CONECT   18   17   20   20   32
CONECT   19   16   20   33
CONECT   20   18   18   19   34
CONECT   21    1
CONECT   22    4
CONECT   23    6
CONECT   24    7
CONECT   25    8
CONECT   26   10
```

```
CONECT   27   11
CONECT   28   13
CONECT   29   14
CONECT   30   15
CONECT   31   17
CONECT   32   18
CONECT   33   19
CONECT   34   20
CONECT   35
MASTER        0    0    0    0    0    0    0    0   35    0   35    0
END


HEADER    Naphthalene host + chloride guest
COMPND    MMFF94 geometry optimized
AUTHOR    GENERATED BY PCMODEL V 9.30
HETATM    1  C   UNK   1         5.205   0.037  -0.012  1.00  0.00
HETATM    2  C   UNK   1         0.270   1.950  -0.566  1.00  0.00
HETATM    3  C   UNK   1         0.241   0.543  -0.603  1.00  0.00
HETATM    4  C   UNK   1         1.429  -0.179  -0.410  1.00  0.00
HETATM    5  C   UNK   1         2.647   0.458  -0.145  1.00  0.00
HETATM    6  C   UNK   1         2.664   1.857  -0.117  1.00  0.00
HETATM    7  C   UNK   1         1.493   2.597  -0.329  1.00  0.00
HETATM    8  N   UNK   1         3.855  -1.632   0.550  1.00  0.00
HETATM    9  C   UNK   1         3.869  -0.319   0.105  1.00  0.00
HETATM   10  C   UNK   1         5.144  -2.086   0.703  1.00  0.00
HETATM   11  C   UNK   1        -0.976  -0.124  -0.818  1.00  0.00
HETATM   12  C   UNK   1        -2.176   0.580  -0.987  1.00  0.00
HETATM   13  C   UNK   1        -2.133   1.979  -0.965  1.00  0.00
HETATM   14  C   UNK   1        -0.926   2.658  -0.758  1.00  0.00
HETATM   15  C   UNK   1         6.003  -1.072   0.360  1.00  0.00
HETATM   16  C   UNK   1        -3.448  -0.129  -1.168  1.00  0.00
HETATM   17  C   UNK   1        -4.626   0.260  -1.788  1.00  0.00
HETATM   18  C   UNK   1        -5.563  -0.795  -1.673  1.00  0.00
HETATM   19  N   UNK   1        -3.673  -1.401  -0.677  1.00  0.00
HETATM   20  C   UNK   1        -4.947  -1.809  -0.980  1.00  0.00
HETATM   21  H   UNK   1         5.571   0.999  -0.343  1.00  0.00
HETATM   22  H   UNK   1         1.388  -1.266  -0.459  1.00  0.00
HETATM   23  H   UNK   1         3.588   2.389   0.092  1.00  0.00
HETATM   24  H   UNK   1         1.543   3.681  -0.290  1.00  0.00
HETATM   25  H   UNK   1         3.021  -2.194   0.746  1.00  0.00
HETATM   26  H   UNK   1         5.313  -3.099   1.043  1.00  0.00
HETATM   27  H   UNK   1        -0.966  -1.213  -0.845  1.00  0.00
HETATM   28  H   UNK   1        -3.044   2.558  -1.086  1.00  0.00
HETATM   29  H   UNK   1        -0.931   3.744  -0.733  1.00  0.00
HETATM   30  H   UNK   1         7.082  -1.124   0.375  1.00  0.00
HETATM   31  H   UNK   1        -4.790   1.207  -2.285  1.00  0.00
HETATM   32  H   UNK   1        -6.574  -0.812  -2.056  1.00  0.00
HETATM   33  H   UNK   1        -2.997  -1.947  -0.156  1.00  0.00
HETATM   34  H   UNK   1        -5.296  -2.785  -0.673  1.00  0.00
HETATM   35 Cl   UNK   1         1.313  -3.800   1.114  1.00  0.00
CONECT    1    9    9   15   21
CONECT    2    3    3    7   14
CONECT    3    2    2    4   11
CONECT    4    3    5    5   22
CONECT    5    4    4    6    9
CONECT    6    5    7    7   23
CONECT    7    2    6    6   24
CONECT    8    9   10   25
CONECT    9    1    1    5    8
CONECT   10    8   15   15   26
CONECT   11    3   12   12   27
CONECT   12   11   11   13   16
CONECT   13   12   14   14   28
```

```
CONECT   14    2   13   13   29
CONECT   15    1   10   10   30
CONECT   16   12   17   17   19
CONECT   17   16   16   18   31
CONECT   18   17   20   20   32
CONECT   19   16   20   33
CONECT   20   18   18   19   34
CONECT   21    1
CONECT   22    4
CONECT   23    6
CONECT   24    7
CONECT   25    8
CONECT   26   10
CONECT   27   11
CONECT   28   13
CONECT   29   14
CONECT   30   15
CONECT   31   17
CONECT   32   18
CONECT   33   19
CONECT   34   20
CONECT   35
MASTER         0    0    0    0    0    0    0    0   35    0   35    0
END


HEADER    Naphthalene host + dihydrogen phosphate guest
COMPND    MMFF94 geometry optimized
AUTHOR    GENERATED BY PCMODEL V 9.30
HETATM    1  C   UNK   1        -5.085  -0.674  -0.628  1.00  0.00
HETATM    2  C   UNK   1        -0.961   2.733  -0.592  1.00  0.00
HETATM    3  C   UNK   1        -0.535   1.413  -0.819  1.00  0.00
HETATM    4  C   UNK   1        -1.462   0.370  -0.849  1.00  0.00
HETATM    5  C   UNK   1        -2.814   0.587  -0.576  1.00  0.00
HETATM    6  C   UNK   1        -3.249   1.903  -0.389  1.00  0.00
HETATM    7  C   UNK   1        -2.336   2.969  -0.410  1.00  0.00
HETATM    8  N   UNK   1        -3.274  -1.795   0.012  1.00  0.00
HETATM    9  C   UNK   1        -3.719  -0.557  -0.430  1.00  0.00
HETATM   10  C   UNK   1        -4.333  -2.670   0.076  1.00  0.00
HETATM   11  C   UNK   1         0.825   1.126  -0.950  1.00  0.00
HETATM   12  C   UNK   1         1.798   2.115  -0.792  1.00  0.00
HETATM   13  C   UNK   1         1.377   3.436  -0.610  1.00  0.00
HETATM   14  C   UNK   1         0.011   3.746  -0.522  1.00  0.00
HETATM   15  C   UNK   1        -5.467  -2.002  -0.318  1.00  0.00
HETATM   16  C   UNK   1         3.215   1.742  -0.758  1.00  0.00
HETATM   17  C   UNK   1         4.357   2.452  -1.091  1.00  0.00
HETATM   18  C   UNK   1         5.481   1.626  -0.850  1.00  0.00
HETATM   19  N   UNK   1         3.638   0.502  -0.300  1.00  0.00
HETATM   20  C   UNK   1         5.009   0.430  -0.363  1.00  0.00
HETATM   21  H   UNK   1        -2.304  -2.021   0.267  1.00  0.00
HETATM   22  H   UNK   1         3.021  -0.245   0.043  1.00  0.00
HETATM   23  P   UNK   1         0.711  -1.904   1.369  1.00  0.00
HETATM   24  O   UNK   1         1.947  -1.569   0.590  1.00  0.00
HETATM   25  O   UNK   1        -0.617  -2.257   0.769  1.00  0.00
HETATM   26  O   UNK   1         0.419  -0.719   2.393  1.00  0.00
HETATM   27  O   UNK   1         1.116  -3.012   2.441  1.00  0.00
HETATM   28  H   UNK   1        -0.558  -0.669   2.424  1.00  0.00
HETATM   29  H   UNK   1         2.075  -2.876   2.568  1.00  0.00
HETATM   30  H   UNK   1        -5.738   0.116  -0.972  1.00  0.00
HETATM   31  H   UNK   1        -1.102  -0.642  -1.031  1.00  0.00
HETATM   32  H   UNK   1        -4.295   2.109  -0.182  1.00  0.00
HETATM   33  H   UNK   1        -2.699   3.977  -0.233  1.00  0.00
HETATM   34  H   UNK   1        -4.166  -3.687   0.400  1.00  0.00
```

```
HETATM   35  H   UNK   1         1.127   0.092  -1.116  1.00  0.00
HETATM   36  H   UNK   1         2.107   4.231  -0.488  1.00  0.00
HETATM   37  H   UNK   1        -0.285   4.777  -0.349  1.00  0.00
HETATM   38  H   UNK   1        -6.461  -2.423  -0.372  1.00  0.00
HETATM   39  H   UNK   1         4.375   3.461  -1.479  1.00  0.00
HETATM   40  H   UNK   1         6.520   1.876  -1.012  1.00  0.00
HETATM   41  H   UNK   1         5.511  -0.473  -0.048  1.00  0.00
CONECT    1    9    9   15   30
CONECT    2    3    3    7   14
CONECT    3    2    2    4   11
CONECT    4    3    5    5   31
CONECT    5    4    4    6    9
CONECT    6    5    7    7   32
CONECT    7    2    6    6   33
CONECT    8    9   10   21
CONECT    9    1    1    5    8
CONECT   10    8   15   15   34
CONECT   11    3   12   12   35
CONECT   12   11   11   13   16
CONECT   13   12   14   14   36
CONECT   14    2   13   13   37
CONECT   15    1   10   10   38
CONECT   16   12   17   17   19
CONECT   17   16   16   18   39
CONECT   18   17   20   20   40
CONECT   19   16   20   22
CONECT   20   18   18   19   41
CONECT   21    8
CONECT   22   19
CONECT   23   24   25   25   26   27
CONECT   24   23
CONECT   25   23   23
CONECT   26   23   28
CONECT   27   23   29
CONECT   28   26
CONECT   29   27
CONECT   30    1
CONECT   31    4
CONECT   32    6
CONECT   33    7
CONECT   34   10
CONECT   35   11
CONECT   36   13
CONECT   37   14
CONECT   38   15
CONECT   39   17
CONECT   40   18
CONECT   41   20
MASTER        0    0    0    0    0    0    0    0   41    0   41    0
END
```

```
HEADER    Naphthalene host + monohydrogen pyrophosphate guest
COMPND    MMFF94 geometry optimized
AUTHOR    GENERATED BY PCMODEL V 9.30
HETATM    1  C   UNK   1         5.036   1.509   0.062  1.00  0.00
HETATM    2  C   UNK   1         0.005   2.785  -1.123  1.00  0.00
HETATM    3  C   UNK   1         0.032   1.515  -0.517  1.00  0.00
HETATM    4  C   UNK   1         1.264   0.937  -0.167  1.00  0.00
HETATM    5  C   UNK   1         2.480   1.585  -0.417  1.00  0.00
HETATM    6  C   UNK   1         2.437   2.853  -1.013  1.00  0.00
HETATM    7  C   UNK   1         1.218   3.449  -1.360  1.00  0.00
```

```
HETATM    8  N   UNK   1         3.906  -0.400   0.168  1.00  0.00
HETATM    9  C   UNK   1         3.768   0.959  -0.077  1.00  0.00
HETATM   10  C   UNK   1         5.224  -0.687   0.450  1.00  0.00
HETATM   11  C   UNK   1        -1.172   0.832  -0.281  1.00  0.00
HETATM   12  C   UNK   1        -2.412   1.368  -0.653  1.00  0.00
HETATM   13  C   UNK   1        -2.423   2.637  -1.250  1.00  0.00
HETATM   14  C   UNK   1        -1.234   3.340  -1.480  1.00  0.00
HETATM   15  C   UNK   1         5.946   0.478   0.393  1.00  0.00
HETATM   16  C   UNK   1        -3.664   0.627  -0.434  1.00  0.00
HETATM   17  C   UNK   1        -4.986   1.058  -0.433  1.00  0.00
HETATM   18  C   UNK   1        -5.826  -0.049  -0.171  1.00  0.00
HETATM   19  N   UNK   1        -3.700  -0.738  -0.179  1.00  0.00
HETATM   20  C   UNK   1        -5.010  -1.141  -0.019  1.00  0.00
HETATM   21  H   UNK   1         3.153  -1.107   0.144  1.00  0.00
HETATM   22  H   UNK   1        -2.887  -1.378  -0.119  1.00  0.00
HETATM   23  P   UNK   1        -1.209  -3.322   1.057  1.00  0.00
HETATM   24  O   UNK   1        -2.139  -4.461   1.436  1.00  0.00
HETATM   25  O   UNK   1        -0.759  -2.486   2.248  1.00  0.00
HETATM   26  O   UNK   1        -1.755  -2.492  -0.093  1.00  0.00
HETATM   27  O   UNK   1         0.147  -4.027   0.529  1.00  0.00
HETATM   28  P   UNK   1         1.672  -3.636   0.860  1.00  0.00
HETATM   29  H   UNK   1         5.277   2.555  -0.052  1.00  0.00
HETATM   30  H   UNK   1         1.247  -0.053   0.297  1.00  0.00
HETATM   31  H   UNK   1         3.358   3.383  -1.232  1.00  0.00
HETATM   32  H   UNK   1         1.225   4.427  -1.830  1.00  0.00
HETATM   33  H   UNK   1         5.491  -1.712   0.671  1.00  0.00
HETATM   34  H   UNK   1        -1.120  -0.152   0.195  1.00  0.00
HETATM   35  H   UNK   1        -3.362   3.082  -1.560  1.00  0.00
HETATM   36  H   UNK   1        -1.283   4.314  -1.954  1.00  0.00
HETATM   37  H   UNK   1         7.006   0.570   0.576  1.00  0.00
HETATM   38  H   UNK   1        -5.309   2.076  -0.589  1.00  0.00
HETATM   39  H   UNK   1        -6.902  -0.055  -0.092  1.00  0.00
HETATM   40  H   UNK   1        -5.197  -2.184   0.200  1.00  0.00
HETATM   41  O   UNK   1         2.545  -4.876   0.749  1.00  0.00
HETATM   42  O   UNK   1         2.148  -2.381   0.155  1.00  0.00
HETATM   43  O   UNK   1         1.552  -3.293   2.431  1.00  0.00
HETATM   44  H   UNK   1         0.655  -2.813   2.495  1.00  0.00
CONECT    1    9    9   15   29
CONECT    2    3    3    7   14
CONECT    3    2    2    4   11
CONECT    4    3    5    5   30
CONECT    5    4    4    6    9
CONECT    6    5    7    7   31
CONECT    7    2    6    6   32
CONECT    8    9   10   21
CONECT    9    1    1    5    8
CONECT   10    8   15   15   33
CONECT   11    3   12   12   34
CONECT   12   11   11   13   16
CONECT   13   12   14   14   35
CONECT   14    2   13   13   36
CONECT   15    1   10   10   37
CONECT   16   12   17   17   19
CONECT   17   16   16   18   38
CONECT   18   17   20   20   39
CONECT   19   16   20   22
CONECT   20   18   18   19   40
CONECT   21    8
CONECT   22   19
CONECT   23   24   25   25   26   27
CONECT   24   23
CONECT   25   23   23
CONECT   26   23
CONECT   27   23   28
```

```
CONECT   28   27   41   42   43
CONECT   29    1
CONECT   30    4
CONECT   31    6
CONECT   32    7
CONECT   33   10
CONECT   34   11
CONECT   35   13
CONECT   36   14
CONECT   37   15
CONECT   38   17
CONECT   39   18
CONECT   40   20
CONECT   41   28
CONECT   42   28
CONECT   43   28   44
CONECT   44   43
MASTER         0    0    0    0    0    0    0    0   44    0   44    0
END
```

```
HEADER    Imido-naphthalene host, global minimum
COMPND    MMFF94 geometry optimized
AUTHOR    GENERATED BY PCMODEL V 9.30
HETATM    1  C   UNK   1       -1.841  -5.429  -3.512  1.00  0.00
HETATM    2  C   UNK   1        0.023  -2.087   0.156  1.00  0.00
HETATM    3  C   UNK   1       -0.039  -3.496   0.158  1.00  0.00
HETATM    4  C   UNK   1       -0.726  -4.158  -0.875  1.00  0.00
HETATM    5  C   UNK   1       -1.356  -3.451  -1.909  1.00  0.00
HETATM    6  C   UNK   1       -1.286  -2.056  -1.898  1.00  0.00
HETATM    7  C   UNK   1       -0.606  -1.382  -0.872  1.00  0.00
HETATM    8  N   UNK   1       -3.156  -3.654  -3.641  1.00  0.00
HETATM    9  C   UNK   1       -2.062  -4.170  -2.974  1.00  0.00
HETATM   10  C   UNK   1       -0.564   0.095  -0.884  1.00  0.00
HETATM   11  C   UNK   1       -3.612  -4.548  -4.573  1.00  0.00
HETATM   12  C   UNK   1        0.588  -4.214   1.192  1.00  0.00
HETATM   13  C   UNK   1        1.278  -3.563   2.225  1.00  0.00
HETATM   14  C   UNK   1        1.330  -2.167   2.211  1.00  0.00
HETATM   15  C   UNK   1        0.712  -1.438   1.184  1.00  0.00
HETATM   16  C   UNK   1       -2.811  -5.663  -4.516  1.00  0.00
HETATM   17  C   UNK   1        1.918  -4.338   3.291  1.00  0.00
HETATM   18  C   UNK   1        0.800   0.037   1.192  1.00  0.00
HETATM   19  C   UNK   1        1.588  -5.572   3.831  1.00  0.00
HETATM   20  C   UNK   1        2.533  -5.888   4.836  1.00  0.00
HETATM   21  N   UNK   1        3.053  -3.919   3.956  1.00  0.00
HETATM   22  C   UNK   1        3.429  -4.848   4.890  1.00  0.00
HETATM   23  N   UNK   1        0.146   0.708   0.153  1.00  0.00
HETATM   24  O   UNK   1        1.436   0.576   2.095  1.00  0.00
HETATM   25  O   UNK   1       -1.150   0.686  -1.788  1.00  0.00
HETATM   26  C   UNK   1        0.209   2.132   0.152  1.00  0.00
HETATM   27  C   UNK   1        1.263   2.785  -0.536  1.00  0.00
HETATM   28  C   UNK   1        1.301   4.190  -0.522  1.00  0.00
HETATM   29  C   UNK   1        0.332   4.929   0.149  1.00  0.00
HETATM   30  C   UNK   1       -0.699   4.280   0.822  1.00  0.00
HETATM   31  C   UNK   1       -0.784   2.876   0.839  1.00  0.00
HETATM   32  C   UNK   1        2.348   2.017  -1.285  1.00  0.00
HETATM   33  C   UNK   1       -1.932   2.208   1.589  1.00  0.00
HETATM   34  C   UNK   1       -1.881   2.520   3.088  1.00  0.00
HETATM   35  C   UNK   1       -3.291   2.590   0.995  1.00  0.00
HETATM   36  C   UNK   1        2.325   2.329  -2.784  1.00  0.00
HETATM   37  C   UNK   1        3.736   2.280  -0.692  1.00  0.00
HETATM   38  H   UNK   1       -3.574  -2.751  -3.457  1.00  0.00
HETATM   39  H   UNK   1        3.549  -3.057   3.771  1.00  0.00
```

```
HETATM   40  H   UNK   1        -1.053  -6.111  -3.220  1.00  0.00
HETATM   41  H   UNK   1        -0.779  -5.246  -0.866  1.00  0.00
HETATM   42  H   UNK   1        -1.742  -1.476  -2.698  1.00  0.00
HETATM   43  H   UNK   1        -4.466  -4.313  -5.193  1.00  0.00
HETATM   44  H   UNK   1         0.546  -5.302   1.185  1.00  0.00
HETATM   45  H   UNK   1         1.836  -1.628   3.009  1.00  0.00
HETATM   46  H   UNK   1        -2.912  -6.549  -5.130  1.00  0.00
HETATM   47  H   UNK   1         0.743  -6.183   3.541  1.00  0.00
HETATM   48  H   UNK   1         2.556  -6.779   5.451  1.00  0.00
HETATM   49  H   UNK   1         4.301  -4.688   5.510  1.00  0.00
HETATM   50  H   UNK   1         2.094   4.727  -1.040  1.00  0.00
HETATM   51  H   UNK   1         0.380   6.015   0.148  1.00  0.00
HETATM   52  H   UNK   1        -1.441   4.884   1.338  1.00  0.00
HETATM   53  H   UNK   1         2.177   0.939  -1.194  1.00  0.00
HETATM   54  H   UNK   1        -1.856   1.119   1.500  1.00  0.00
HETATM   55  H   UNK   1        -2.037   3.584   3.293  1.00  0.00
HETATM   56  H   UNK   1        -0.913   2.235   3.514  1.00  0.00
HETATM   57  H   UNK   1        -2.659   1.963   3.622  1.00  0.00
HETATM   58  H   UNK   1        -3.508   3.657   1.114  1.00  0.00
HETATM   59  H   UNK   1        -4.096   2.034   1.488  1.00  0.00
HETATM   60  H   UNK   1        -3.330   2.357  -0.075  1.00  0.00
HETATM   61  H   UNK   1         2.573   3.375  -2.992  1.00  0.00
HETATM   62  H   UNK   1         1.335   2.129  -3.210  1.00  0.00
HETATM   63  H   UNK   1         3.050   1.705  -3.317  1.00  0.00
HETATM   64  H   UNK   1         4.045   3.324  -0.812  1.00  0.00
HETATM   65  H   UNK   1         4.488   1.654  -1.184  1.00  0.00
HETATM   66  H   UNK   1         3.753   2.046   0.379  1.00  0.00
CONECT    1    9    9   16   40
CONECT    2    3    3    7   15
CONECT    3    2    2    4   12
CONECT    4    3    5    5   41
CONECT    5    4    4    6    9
CONECT    6    5    7    7   42
CONECT    7    2    6    6   10
CONECT    8    9   11   38
CONECT    9    1    1    5    8
CONECT   10    7   23   25   25
CONECT   11    8   16   16   43
CONECT   12    3   13   13   44
CONECT   13   12   12   14   17
CONECT   14   13   15   15   45
CONECT   15    2   14   14   18
CONECT   16    1   11   11   46
CONECT   17   13   19   19   21
CONECT   18   15   23   24   24
CONECT   19   17   17   20   47
CONECT   20   19   22   22   48
CONECT   21   17   22   39
CONECT   22   20   20   21   49
CONECT   23   10   18   26
CONECT   24   18   18
CONECT   25   10   10
CONECT   26   23   27   27   31
CONECT   27   26   26   28   32
CONECT   28   27   29   29   50
CONECT   29   28   28   30   51
CONECT   30   29   31   31   52
CONECT   31   26   30   30   33
CONECT   32   27   36   37   53
CONECT   33   31   34   35   54
CONECT   34   33   55   56   57
CONECT   35   33   58   59   60
CONECT   36   32   61   62   63
CONECT   37   32   64   65   66
```

```
CONECT   38    8
CONECT   39   21
CONECT   40    1
CONECT   41    4
CONECT   42    6
CONECT   43   11
CONECT   44   12
CONECT   45   14
CONECT   46   16
CONECT   47   19
CONECT   48   20
CONECT   49   22
CONECT   50   28
CONECT   51   29
CONECT   52   30
CONECT   53   32
CONECT   54   33
CONECT   55   34
CONECT   56   34
CONECT   57   34
CONECT   58   35
CONECT   59   35
CONECT   60   35
CONECT   61   36
CONECT   62   36
CONECT   63   36
CONECT   64   37
CONECT   65   37
CONECT   66   37
MASTER         0    0    0    0    0    0    0    0   66    0   66    0
END
```

```
HEADER    Imido-naphthalene host, in free naphthalene host conformation
COMPND    MMFF94 geometry optimized
AUTHOR    GENERATED BY PCMODEL V 9.30
HETATM    1  C   UNK   1        5.714  -1.606   0.608  1.00  0.00
HETATM    2  C   UNK   1        0.723  -0.034  -0.199  1.00  0.00
HETATM    3  C   UNK   1        1.454   0.968   0.478  1.00  0.00
HETATM    4  C   UNK   1        2.757   0.690   0.928  1.00  0.00
HETATM    5  C   UNK   1        3.350  -0.562   0.716  1.00  0.00
HETATM    6  C   UNK   1        2.621  -1.541   0.036  1.00  0.00
HETATM    7  C   UNK   1        1.319  -1.279  -0.414  1.00  0.00
HETATM    8  N   UNK   1        5.211  -0.374   2.377  1.00  0.00
HETATM    9  C   UNK   1        4.708  -0.844   1.181  1.00  0.00
HETATM   10  C   UNK   1        0.572  -2.339  -1.124  1.00  0.00
HETATM   11  C   UNK   1        6.500  -0.803   2.551  1.00  0.00
HETATM   12  C   UNK   1        0.866   2.228   0.692  1.00  0.00
HETATM   13  C   UNK   1       -0.432   2.511   0.250  1.00  0.00
HETATM   14  C   UNK   1       -1.148   1.509  -0.409  1.00  0.00
HETATM   15  C   UNK   1       -0.574   0.250  -0.633  1.00  0.00
HETATM   16  C   UNK   1        6.840  -1.573   1.465  1.00  0.00
HETATM   17  C   UNK   1       -1.040   3.822   0.479  1.00  0.00
HETATM   18  C   UNK   1       -1.357  -0.787  -1.339  1.00  0.00
HETATM   19  C   UNK   1       -2.351   4.166   0.770  1.00  0.00
HETATM   20  C   UNK   1       -2.426   5.575   0.888  1.00  0.00
HETATM   21  N   UNK   1       -0.337   5.007   0.401  1.00  0.00
HETATM   22  C   UNK   1       -1.165   6.068   0.654  1.00  0.00
HETATM   23  N   UNK   1       -0.724  -2.018  -1.539  1.00  0.00
HETATM   24  O   UNK   1       -2.497  -0.511  -1.702  1.00  0.00
HETATM   25  O   UNK   1        1.125  -3.421  -1.305  1.00  0.00
HETATM   26  C   UNK   1       -1.461  -3.029  -2.222  1.00  0.00
```

```
HETATM   27  C   UNK   1        -1.379  -3.120  -3.635  1.00  0.00
HETATM   28  C   UNK   1        -2.117  -4.127  -4.284  1.00  0.00
HETATM   29  C   UNK   1        -2.910  -5.015  -3.564  1.00  0.00
HETATM   30  C   UNK   1        -2.984  -4.917  -2.178  1.00  0.00
HETATM   31  C   UNK   1        -2.267  -3.930  -1.479  1.00  0.00
HETATM   32  C   UNK   1        -0.524  -2.169  -4.466  1.00  0.00
HETATM   33  C   UNK   1        -2.379  -3.861   0.040  1.00  0.00
HETATM   34  C   UNK   1        -3.814  -3.565   0.486  1.00  0.00
HETATM   35  C   UNK   1        -1.852  -5.138   0.702  1.00  0.00
HETATM   36  C   UNK   1         0.582  -2.918  -5.217  1.00  0.00
HETATM   37  C   UNK   1        -1.378  -1.341  -5.431  1.00  0.00
HETATM   38  H   UNK   1         4.695   0.185   3.042  1.00  0.00
HETATM   39  H   UNK   1         0.643   5.083   0.166  1.00  0.00
HETATM   40  H   UNK   1         5.648  -2.129  -0.338  1.00  0.00
HETATM   41  H   UNK   1         3.323   1.471   1.431  1.00  0.00
HETATM   42  H   UNK   1         3.061  -2.522  -0.137  1.00  0.00
HETATM   43  H   UNK   1         7.060  -0.531   3.436  1.00  0.00
HETATM   44  H   UNK   1         1.426   2.990   1.231  1.00  0.00
HETATM   45  H   UNK   1        -2.159   1.709  -0.765  1.00  0.00
HETATM   46  H   UNK   1         7.792  -2.062   1.306  1.00  0.00
HETATM   47  H   UNK   1        -3.173   3.472   0.894  1.00  0.00
HETATM   48  H   UNK   1        -3.306   6.163   1.113  1.00  0.00
HETATM   49  H   UNK   1        -0.787   7.082   0.639  1.00  0.00
HETATM   50  H   UNK   1        -2.079  -4.228  -5.366  1.00  0.00
HETATM   51  H   UNK   1        -3.472  -5.786  -4.085  1.00  0.00
HETATM   52  H   UNK   1        -3.612  -5.626  -1.642  1.00  0.00
HETATM   53  H   UNK   1        -0.017  -1.451  -3.813  1.00  0.00
HETATM   54  H   UNK   1        -1.764  -3.041   0.426  1.00  0.00
HETATM   55  H   UNK   1        -4.503  -4.374   0.224  1.00  0.00
HETATM   56  H   UNK   1        -4.185  -2.645   0.021  1.00  0.00
HETATM   57  H   UNK   1        -3.859  -3.432   1.573  1.00  0.00
HETATM   58  H   UNK   1        -2.459  -6.014   0.448  1.00  0.00
HETATM   59  H   UNK   1        -1.859  -5.036   1.792  1.00  0.00
HETATM   60  H   UNK   1        -0.822  -5.343   0.391  1.00  0.00
HETATM   61  H   UNK   1         0.178  -3.606  -5.967  1.00  0.00
HETATM   62  H   UNK   1         1.199  -3.501  -4.524  1.00  0.00
HETATM   63  H   UNK   1         1.239  -2.211  -5.737  1.00  0.00
HETATM   64  H   UNK   1        -1.864  -1.963  -6.189  1.00  0.00
HETATM   65  H   UNK   1        -0.759  -0.604  -5.955  1.00  0.00
HETATM   66  H   UNK   1        -2.161  -0.798  -4.891  1.00  0.00
CONECT    1    9    9   16   40
CONECT    2    3    3    7   15
CONECT    3    2    2    4   12
CONECT    4    3    5    5   41
CONECT    5    4    4    6    9
CONECT    6    5    7    7   42
CONECT    7    2    6    6   10
CONECT    8    9   11   38
CONECT    9    1    1    5    8
CONECT   10    7   23   25   25
CONECT   11    8   16   16   43
CONECT   12    3   13   13   44
CONECT   13   12   12   14   17
CONECT   14   13   15   15   45
CONECT   15    2   14   14   18
CONECT   16    1   11   11   46
CONECT   17   13   19   19   21
CONECT   18   15   23   24   24
CONECT   19   17   17   20   47
CONECT   20   19   22   22   48
CONECT   21   17   22   39
CONECT   22   20   20   21   49
CONECT   23   10   18   26
CONECT   24   18   18
```

```
CONECT   25   10   10
CONECT   26   23   27   27   31
CONECT   27   26   26   28   32
CONECT   28   27   29   29   50
CONECT   29   28   28   30   51
CONECT   30   29   31   31   52
CONECT   31   26   30   30   33
CONECT   32   27   36   37   53
CONECT   33   31   34   35   54
CONECT   34   33   55   56   57
CONECT   35   33   58   59   60
CONECT   36   32   61   62   63
CONECT   37   32   64   65   66
CONECT   38    8
CONECT   39   21
CONECT   40    1
CONECT   41    4
CONECT   42    6
CONECT   43   11
CONECT   44   12
CONECT   45   14
CONECT   46   16
CONECT   47   19
CONECT   48   20
CONECT   49   22
CONECT   50   28
CONECT   51   29
CONECT   52   30
CONECT   53   32
CONECT   54   33
CONECT   55   34
CONECT   56   34
CONECT   57   34
CONECT   58   35
CONECT   59   35
CONECT   60   35
CONECT   61   36
CONECT   62   36
CONECT   63   36
CONECT   64   37
CONECT   65   37
CONECT   66   37
MASTER         0    0    0    0    0    0    0    0   66    0   66    0
END


HEADER    Imido-naphthalene host, in anion binding conformation
COMPND    MMFF94 geometry optimized
AUTHOR    GENERATED BY PCMODEL V 9.30
HETATM    1  C   UNK   1         3.882  -2.374  -0.095  1.00  0.00
HETATM    2  C   UNK   1        -1.111  -0.638   0.195  1.00  0.00
HETATM    3  C   UNK   1        -1.058  -2.023   0.467  1.00  0.00
HETATM    4  C   UNK   1         0.192  -2.656   0.588  1.00  0.00
HETATM    5  C   UNK   1         1.389  -1.941   0.449  1.00  0.00
HETATM    6  C   UNK   1         1.322  -0.573   0.177  1.00  0.00
HETATM    7  C   UNK   1         0.084   0.072   0.052  1.00  0.00
HETATM    8  N   UNK   1         2.936  -3.627   1.465  1.00  0.00
HETATM    9  C   UNK   1         2.688  -2.606   0.571  1.00  0.00
HETATM   10  C   UNK   1         0.049   1.521  -0.236  1.00  0.00
HETATM   11  C   UNK   1         4.236  -4.046   1.361  1.00  0.00
HETATM   12  C   UNK   1        -2.255  -2.747   0.606  1.00  0.00
HETATM   13  C   UNK   1        -3.504  -2.124   0.484  1.00  0.00
HETATM   14  C   UNK   1        -3.543  -0.754   0.212  1.00  0.00
```

```
HETATM   15  C   UNK   1        -2.357  -0.019   0.070  1.00   0.00
HETATM   16  C   UNK   1         4.849  -3.283   0.396  1.00   0.00
HETATM   17  C   UNK   1        -4.747  -2.882   0.624  1.00   0.00
HETATM   18  C   UNK   1        -2.434   1.429  -0.218  1.00   0.00
HETATM   19  C   UNK   1        -5.964  -2.741  -0.025  1.00   0.00
HETATM   20  C   UNK   1        -6.855  -3.719   0.479  1.00   0.00
HETATM   21  N   UNK   1        -4.906  -3.919   1.521  1.00   0.00
HETATM   22  C   UNK   1        -6.173  -4.433   1.434  1.00   0.00
HETATM   23  N   UNK   1        -1.217   2.104  -0.353  1.00   0.00
HETATM   24  O   UNK   1        -3.544   1.946  -0.320  1.00   0.00
HETATM   25  O   UNK   1         1.116   2.119  -0.354  1.00   0.00
HETATM   26  C   UNK   1        -1.271   3.501  -0.632  1.00   0.00
HETATM   27  C   UNK   1        -1.298   4.429   0.440  1.00   0.00
HETATM   28  C   UNK   1        -1.351   5.801   0.136  1.00   0.00
HETATM   29  C   UNK   1        -1.377   6.244  -1.183  1.00   0.00
HETATM   30  C   UNK   1        -1.350   5.327  -2.228  1.00   0.00
HETATM   31  C   UNK   1        -1.297   3.944  -1.980  1.00   0.00
HETATM   32  C   UNK   1        -1.271   3.989   1.900  1.00   0.00
HETATM   33  C   UNK   1        -1.269   2.974  -3.157  1.00   0.00
HETATM   34  C   UNK   1        -2.542   3.080  -4.003  1.00   0.00
HETATM   35  C   UNK   1        -0.020   3.174  -4.021  1.00   0.00
HETATM   36  C   UNK   1        -0.022   4.506   2.621  1.00   0.00
HETATM   37  C   UNK   1        -2.545   4.412   2.639  1.00   0.00
HETATM   38  H   UNK   1         2.263  -3.998   2.121  1.00   0.00
HETATM   39  H   UNK   1        -4.198  -4.238   2.167  1.00   0.00
HETATM   40  H   UNK   1         4.040  -1.630  -0.865  1.00   0.00
HETATM   41  H   UNK   1         0.228  -3.729   0.770  1.00   0.00
HETATM   42  H   UNK   1         2.240   0.004   0.069  1.00   0.00
HETATM   43  H   UNK   1         4.614  -4.844   1.986  1.00   0.00
HETATM   44  H   UNK   1        -2.208  -3.820   0.787  1.00   0.00
HETATM   45  H   UNK   1        -4.502  -0.247   0.117  1.00   0.00
HETATM   46  H   UNK   1         5.882  -3.366   0.081  1.00   0.00
HETATM   47  H   UNK   1        -6.188  -2.011  -0.793  1.00   0.00
HETATM   48  H   UNK   1        -7.882  -3.879   0.178  1.00   0.00
HETATM   49  H   UNK   1        -6.481  -5.257   2.064  1.00   0.00
HETATM   50  H   UNK   1        -1.373   6.542   0.932  1.00   0.00
HETATM   51  H   UNK   1        -1.418   7.309  -1.396  1.00   0.00
HETATM   52  H   UNK   1        -1.372   5.704  -3.248  1.00   0.00
HETATM   53  H   UNK   1        -1.230   2.897   1.963  1.00   0.00
HETATM   54  H   UNK   1        -1.228   1.942  -2.794  1.00   0.00
HETATM   55  H   UNK   1        -2.635   4.057  -4.489  1.00   0.00
HETATM   56  H   UNK   1        -3.435   2.926  -3.386  1.00   0.00
HETATM   57  H   UNK   1        -2.544   2.318  -4.790  1.00   0.00
HETATM   58  H   UNK   1        -0.007   4.155  -4.507  1.00   0.00
HETATM   59  H   UNK   1         0.027   2.414  -4.809  1.00   0.00
HETATM   60  H   UNK   1         0.890   3.087  -3.417  1.00   0.00
HETATM   61  H   UNK   1        -0.009   5.599   2.692  1.00   0.00
HETATM   62  H   UNK   1         0.888   4.193   2.098  1.00   0.00
HETATM   63  H   UNK   1         0.024   4.108   3.641  1.00   0.00
HETATM   64  H   UNK   1        -2.638   5.501   2.710  1.00   0.00
HETATM   65  H   UNK   1        -2.547   4.013   3.659  1.00   0.00
HETATM   66  H   UNK   1        -3.437   4.032   2.128  1.00   0.00
CONECT    1     9    9   16   40
CONECT    2     3    3    7   15
CONECT    3     2    2    4   12
CONECT    4     3    5    5   41
CONECT    5     4    4    6    9
CONECT    6     5    7    7   42
CONECT    7     2    6    6   10
CONECT    8     9   11   38
CONECT    9     1    1    5    8
CONECT   10     7   23   25   25
CONECT   11     8   16   16   43
CONECT   12     3   13   13   44
```

```
CONECT   13   12   12   14   17
CONECT   14   13   15   15   45
CONECT   15    2   14   14   18
CONECT   16    1   11   11   46
CONECT   17   13   19   19   21
CONECT   18   15   23   24   24
CONECT   19   17   17   20   47
CONECT   20   19   22   22   48
CONECT   21   17   22   39
CONECT   22   20   20   21   49
CONECT   23   10   18   26
CONECT   24   18   18
CONECT   25   10   10
CONECT   26   23   27   27   31
CONECT   27   26   26   28   32
CONECT   28   27   29   29   50
CONECT   29   28   28   30   51
CONECT   30   29   31   31   52
CONECT   31   26   30   30   33
CONECT   32   27   36   37   53
CONECT   33   31   34   35   54
CONECT   34   33   55   56   57
CONECT   35   33   58   59   60
CONECT   36   32   61   62   63
CONECT   37   32   64   65   66
CONECT   38    8
CONECT   39   21
CONECT   40    1
CONECT   41    4
CONECT   42    6
CONECT   43   11
CONECT   44   12
CONECT   45   14
CONECT   46   16
CONECT   47   19
CONECT   48   20
CONECT   49   22
CONECT   50   28
CONECT   51   29
CONECT   52   30
CONECT   53   32
CONECT   54   33
CONECT   55   34
CONECT   56   34
CONECT   57   34
CONECT   58   35
CONECT   59   35
CONECT   60   35
CONECT   61   36
CONECT   62   36
CONECT   63   36
CONECT   64   37
CONECT   65   37
CONECT   66   37
MASTER         0    0    0    0    0    0    0    0   66    0   66    0
END


HEADER    Imido-naphthalene host + fluoride guest
COMPND    MMFF94 geometry optimized
AUTHOR    GENERATED BY PCMODEL V 9.30
HETATM    1  C   UNK   1        -5.035  -2.972   1.507  1.00  0.00
HETATM    2  C   UNK   1        -0.042  -1.211   0.982  1.00  0.00
```

HETATM	3	C	UNK	1	-0.038	-2.574	1.331	1.00	0.00
HETATM	4	C	UNK	1	-1.250	-3.275	1.436	1.00	0.00
HETATM	5	C	UNK	1	-2.479	-2.651	1.190	1.00	0.00
HETATM	6	C	UNK	1	-2.475	-1.292	0.859	1.00	0.00
HETATM	7	C	UNK	1	-1.269	-0.581	0.757	1.00	0.00
HETATM	8	N	UNK	1	-3.814	-4.766	1.070	1.00	0.00
HETATM	9	C	UNK	1	-3.738	-3.400	1.267	1.00	0.00
HETATM	10	C	UNK	1	-1.295	0.850	0.394	1.00	0.00
HETATM	11	C	UNK	1	-5.111	-5.193	1.190	1.00	0.00
HETATM	12	C	UNK	1	1.181	-3.225	1.558	1.00	0.00
HETATM	13	C	UNK	1	2.410	-2.571	1.414	1.00	0.00
HETATM	14	C	UNK	1	2.394	-1.214	1.075	1.00	0.00
HETATM	15	C	UNK	1	1.180	-0.542	0.866	1.00	0.00
HETATM	16	C	UNK	1	-5.894	-4.097	1.463	1.00	0.00
HETATM	17	C	UNK	1	3.675	-3.292	1.603	1.00	0.00
HETATM	18	C	UNK	1	1.193	0.891	0.507	1.00	0.00
HETATM	19	C	UNK	1	4.949	-2.819	1.891	1.00	0.00
HETATM	20	C	UNK	1	5.834	-3.921	1.961	1.00	0.00
HETATM	21	N	UNK	1	3.788	-4.670	1.490	1.00	0.00
HETATM	22	C	UNK	1	5.093	-5.048	1.709	1.00	0.00
HETATM	23	N	UNK	1	-0.054	1.490	0.288	1.00	0.00
HETATM	24	O	UNK	1	2.273	1.473	0.428	1.00	0.00
HETATM	25	O	UNK	1	-2.384	1.392	0.213	1.00	0.00
HETATM	26	C	UNK	1	-0.062	2.871	-0.066	1.00	0.00
HETATM	27	C	UNK	1	-0.127	3.855	0.954	1.00	0.00
HETATM	28	C	UNK	1	-0.134	5.210	0.577	1.00	0.00
HETATM	29	C	UNK	1	-0.078	5.584	-0.763	1.00	0.00
HETATM	30	C	UNK	1	-0.013	4.611	-1.756	1.00	0.00
HETATM	31	C	UNK	1	-0.003	3.243	-1.434	1.00	0.00
HETATM	32	C	UNK	1	-0.188	3.490	2.434	1.00	0.00
HETATM	33	C	UNK	1	0.071	2.210	-2.554	1.00	0.00
HETATM	34	C	UNK	1	-1.148	2.294	-3.478	1.00	0.00
HETATM	35	C	UNK	1	1.372	2.338	-3.352	1.00	0.00
HETATM	36	C	UNK	1	1.031	4.021	3.194	1.00	0.00
HETATM	37	C	UNK	1	-1.490	3.975	3.079	1.00	0.00
HETATM	38	H	UNK	1	-3.025	-5.365	0.853	1.00	0.00
HETATM	39	H	UNK	1	3.025	-5.328	1.271	1.00	0.00
HETATM	40	F	UNK	1	1.710	-6.628	0.980	1.00	0.00
HETATM	41	H	UNK	1	-5.335	-1.951	1.704	1.00	0.00
HETATM	42	H	UNK	1	-1.210	-4.329	1.710	1.00	0.00
HETATM	43	H	UNK	1	-3.414	-0.780	0.657	1.00	0.00
HETATM	44	H	UNK	1	-5.358	-6.239	1.068	1.00	0.00
HETATM	45	H	UNK	1	1.163	-4.277	1.842	1.00	0.00
HETATM	46	H	UNK	1	3.332	-0.677	0.952	1.00	0.00
HETATM	47	H	UNK	1	-6.965	-4.104	1.614	1.00	0.00
HETATM	48	H	UNK	1	5.214	-1.782	2.044	1.00	0.00
HETATM	49	H	UNK	1	6.894	-3.894	2.170	1.00	0.00
HETATM	50	H	UNK	1	5.354	-6.096	1.660	1.00	0.00
HETATM	51	H	UNK	1	-0.183	5.991	1.331	1.00	0.00
HETATM	52	H	UNK	1	-0.083	6.636	-1.033	1.00	0.00
HETATM	53	H	UNK	1	0.032	4.932	-2.794	1.00	0.00
HETATM	54	H	UNK	1	-0.174	2.402	2.554	1.00	0.00
HETATM	55	H	UNK	1	0.067	1.198	-2.136	1.00	0.00
HETATM	56	H	UNK	1	-1.191	3.245	-4.019	1.00	0.00
HETATM	57	H	UNK	1	-2.078	2.192	-2.908	1.00	0.00
HETATM	58	H	UNK	1	-1.122	1.491	-4.223	1.00	0.00
HETATM	59	H	UNK	1	1.436	3.292	-3.887	1.00	0.00
HETATM	60	H	UNK	1	1.448	1.537	-4.094	1.00	0.00
HETATM	61	H	UNK	1	2.244	2.266	-2.692	1.00	0.00
HETATM	62	H	UNK	1	1.064	5.115	3.210	1.00	0.00
HETATM	63	H	UNK	1	1.961	3.664	2.738	1.00	0.00
HETATM	64	H	UNK	1	1.014	3.676	4.234	1.00	0.00
HETATM	65	H	UNK	1	-1.563	5.068	3.090	1.00	0.00
HETATM	66	H	UNK	1	-1.556	3.630	4.117	1.00	0.00

```
HETATM   67  H   UNK   1        -2.362   3.587   2.541  1.00  0.00
CONECT    1    9    9   16   41
CONECT    2    3    3    7   15
CONECT    3    2    2    4   12
CONECT    4    3    5    5   42
CONECT    5    4    4    6    9
CONECT    6    5    7    7   43
CONECT    7    2    6    6   10
CONECT    8    9   11   38
CONECT    9    1    1    5    8
CONECT   10    7   23   25   25
CONECT   11    8   16   16   44
CONECT   12    3   13   13   45
CONECT   13   12   12   14   17
CONECT   14   13   15   15   46
CONECT   15    2   14   14   18
CONECT   16    1   11   11   47
CONECT   17   13   19   19   21
CONECT   18   15   23   24   24
CONECT   19   17   17   20   48
CONECT   20   19   22   22   49
CONECT   21   17   22   39
CONECT   22   20   20   21   50
CONECT   23   10   18   26
CONECT   24   18   18
CONECT   25   10   10
CONECT   26   23   27   27   31
CONECT   27   26   26   28   32
CONECT   28   27   29   29   51
CONECT   29   28   28   30   52
CONECT   30   29   31   31   53
CONECT   31   26   30   30   33
CONECT   32   27   36   37   54
CONECT   33   31   34   35   55
CONECT   34   33   56   57   58
CONECT   35   33   59   60   61
CONECT   36   32   62   63   64
CONECT   37   32   65   66   67
CONECT   38    8
CONECT   39   21
CONECT   40
CONECT   41    1
CONECT   42    4
CONECT   43    6
CONECT   44   11
CONECT   45   12
CONECT   46   14
CONECT   47   16
CONECT   48   19
CONECT   49   20
CONECT   50   22
CONECT   51   28
CONECT   52   29
CONECT   53   30
CONECT   54   32
CONECT   55   33
CONECT   56   34
CONECT   57   34
CONECT   58   34
CONECT   59   35
CONECT   60   35
CONECT   61   35
CONECT   62   36
CONECT   63   36
```

```
CONECT   64   36
CONECT   65   37
CONECT   66   37
CONECT   67   37
MASTER        0    0    0    0    0    0    0    0   67    0   67    0
END
```

```
HEADER    Imido-naphthalene host + chloride guest
COMPND    MMFF94 geometry optimized
AUTHOR    GENERATED BY PCMODEL V 9.30
HETATM    1  C   UNK   1        -5.002  -3.187   2.005  1.00  0.00
HETATM    2  C   UNK   1        -0.034  -1.479   1.155  1.00  0.00
HETATM    3  C   UNK   1        -0.022  -2.821   1.575  1.00  0.00
HETATM    4  C   UNK   1        -1.231  -3.506   1.771  1.00  0.00
HETATM    5  C   UNK   1        -2.466  -2.886   1.548  1.00  0.00
HETATM    6  C   UNK   1        -2.470  -1.545   1.152  1.00  0.00
HETATM    7  C   UNK   1        -1.265  -0.850   0.959  1.00  0.00
HETATM    8  N   UNK   1        -3.810  -4.997   1.547  1.00  0.00
HETATM    9  C   UNK   1        -3.721  -3.627   1.709  1.00  0.00
HETATM   10  C   UNK   1        -1.299   0.562   0.526  1.00  0.00
HETATM   11  C   UNK   1        -5.101  -5.414   1.746  1.00  0.00
HETATM   12  C   UNK   1         1.201  -3.473   1.774  1.00  0.00
HETATM   13  C   UNK   1         2.424  -2.845   1.517  1.00  0.00
HETATM   14  C   UNK   1         2.405  -1.508   1.112  1.00  0.00
HETATM   15  C   UNK   1         1.187  -0.831   0.940  1.00  0.00
HETATM   16  C   UNK   1        -5.865  -4.309   2.033  1.00  0.00
HETATM   17  C   UNK   1         3.685  -3.582   1.652  1.00  0.00
HETATM   18  C   UNK   1         1.192   0.581   0.508  1.00  0.00
HETATM   19  C   UNK   1         4.975  -3.133   1.894  1.00  0.00
HETATM   20  C   UNK   1         5.843  -4.250   1.917  1.00  0.00
HETATM   21  N   UNK   1         3.771  -4.960   1.515  1.00  0.00
HETATM   22  C   UNK   1         5.074  -5.363   1.680  1.00  0.00
HETATM   23  N   UNK   1        -0.060   1.182   0.322  1.00  0.00
HETATM   24  O   UNK   1         2.271   1.146   0.344  1.00  0.00
HETATM   25  O   UNK   1        -2.391   1.106   0.377  1.00  0.00
HETATM   26  C   UNK   1        -0.074   2.543  -0.102  1.00  0.00
HETATM   27  C   UNK   1        -0.078   3.579   0.867  1.00  0.00
HETATM   28  C   UNK   1        -0.093   4.912   0.421  1.00  0.00
HETATM   29  C   UNK   1        -0.104   5.216  -0.937  1.00  0.00
HETATM   30  C   UNK   1        -0.100   4.193  -1.881  1.00  0.00
HETATM   31  C   UNK   1        -0.085   2.842  -1.488  1.00  0.00
HETATM   32  C   UNK   1        -0.065   3.291   2.365  1.00  0.00
HETATM   33  C   UNK   1        -0.080   1.753  -2.556  1.00  0.00
HETATM   34  C   UNK   1        -1.346   1.803  -3.417  1.00  0.00
HETATM   35  C   UNK   1         1.178   1.825  -3.427  1.00  0.00
HETATM   36  C   UNK   1         1.197   3.847   3.033  1.00  0.00
HETATM   37  C   UNK   1        -1.327   3.824   3.051  1.00  0.00
HETATM   38  H   UNK   1        -3.036  -5.604   1.302  1.00  0.00
HETATM   39  H   UNK   1         2.990  -5.592   1.312  1.00  0.00
HETATM   40 Cl   UNK   1         1.234  -7.194   0.999  1.00  0.00
HETATM   41  H   UNK   1        -5.288  -2.160   2.192  1.00  0.00
HETATM   42  H   UNK   1        -1.188  -4.548   2.092  1.00  0.00
HETATM   43  H   UNK   1        -3.415  -1.036   0.968  1.00  0.00
HETATM   44  H   UNK   1        -5.356  -6.462   1.661  1.00  0.00
HETATM   45  H   UNK   1         1.191  -4.504   2.123  1.00  0.00
HETATM   46  H   UNK   1         3.340  -0.991   0.905  1.00  0.00
HETATM   47  H   UNK   1        -6.927  -4.308   2.239  1.00  0.00
HETATM   48  H   UNK   1         5.262  -2.101   2.048  1.00  0.00
HETATM   49  H   UNK   1         6.911  -4.244   2.085  1.00  0.00
HETATM   50  H   UNK   1         5.320  -6.414   1.608  1.00  0.00
HETATM   51  H   UNK   1        -0.096   5.732   1.135  1.00  0.00
HETATM   52  H   UNK   1        -0.115   6.253  -1.262  1.00  0.00
```

```
HETATM   53  H   UNK   1        -0.108   4.459  -2.935  1.00  0.00
HETATM   54  H   UNK   1        -0.054   2.211   2.541  1.00  0.00
HETATM   55  H   UNK   1        -0.070   0.764  -2.086  1.00  0.00
HETATM   56  H   UNK   1        -1.409   2.725  -4.004  1.00  0.00
HETATM   57  H   UNK   1        -2.245   1.740  -2.794  1.00  0.00
HETATM   58  H   UNK   1        -1.366   0.962  -4.119  1.00  0.00
HETATM   59  H   UNK   1         1.221   2.749  -4.014  1.00  0.00
HETATM   60  H   UNK   1         1.207   0.985  -4.130  1.00  0.00
HETATM   61  H   UNK   1         2.083   1.778  -2.811  1.00  0.00
HETATM   62  H   UNK   1         1.240   4.940   2.990  1.00  0.00
HETATM   63  H   UNK   1         2.099   3.457   2.549  1.00  0.00
HETATM   64  H   UNK   1         1.232   3.556   4.089  1.00  0.00
HETATM   65  H   UNK   1        -1.390   4.916   3.008  1.00  0.00
HETATM   66  H   UNK   1        -1.341   3.533   4.107  1.00  0.00
HETATM   67  H   UNK   1        -2.229   3.417   2.579  1.00  0.00
CONECT    1    9    9   16   41
CONECT    2    3    3    7   15
CONECT    3    2    2    4   12
CONECT    4    3    5    5   42
CONECT    5    4    4    6    9
CONECT    6    5    7    7   43
CONECT    7    2    6    6   10
CONECT    8    9   11   38
CONECT    9    1    1    5    8
CONECT   10    7   23   25   25
CONECT   11    8   16   16   44
CONECT   12    3   13   13   45
CONECT   13   12   12   14   17
CONECT   14   13   15   15   46
CONECT   15    2   14   14   18
CONECT   16    1   11   11   47
CONECT   17   13   19   19   21
CONECT   18   15   23   24   24
CONECT   19   17   17   20   48
CONECT   20   19   22   22   49
CONECT   21   17   22   39
CONECT   22   20   20   21   50
CONECT   23   10   18   26
CONECT   24   18   18
CONECT   25   10   10
CONECT   26   23   27   27   31
CONECT   27   26   26   28   32
CONECT   28   27   29   29   51
CONECT   29   28   28   30   52
CONECT   30   29   31   31   53
CONECT   31   26   30   30   33
CONECT   32   27   36   37   54
CONECT   33   31   34   35   55
CONECT   34   33   56   57   58
CONECT   35   33   59   60   61
CONECT   36   32   62   63   64
CONECT   37   32   65   66   67
CONECT   38    8
CONECT   39   21
CONECT   40
CONECT   41    1
CONECT   42    4
CONECT   43    6
CONECT   44   11
CONECT   45   12
CONECT   46   14
CONECT   47   16
CONECT   48   19
CONECT   49   20
```

```
CONECT   50   22
CONECT   51   28
CONECT   52   29
CONECT   53   30
CONECT   54   32
CONECT   55   33
CONECT   56   34
CONECT   57   34
CONECT   58   34
CONECT   59   35
CONECT   60   35
CONECT   61   35
CONECT   62   36
CONECT   63   36
CONECT   64   36
CONECT   65   37
CONECT   66   37
CONECT   67   37
MASTER        0    0    0    0    0    0    0    0   67    0   67    0
END


HEADER    Imido-naphthalene host + dihydrogen phosphate guest
COMPND    MMFF94 geometry optimized
AUTHOR    GENERATED BY PCMODEL V 9.30
HETATM    1  C   UNK   1        -4.728  -3.440  -1.292  1.00  0.00
HETATM    2  C   UNK   1         0.231  -1.526  -0.743  1.00  0.00
HETATM    3  C   UNK   1         0.241  -2.912  -0.963  1.00  0.00
HETATM    4  C   UNK   1        -0.960  -3.607  -1.112  1.00  0.00
HETATM    5  C   UNK   1        -2.195  -2.970  -0.973  1.00  0.00
HETATM    6  C   UNK   1        -2.208  -1.583  -0.795  1.00  0.00
HETATM    7  C   UNK   1        -1.002  -0.868  -0.691  1.00  0.00
HETATM    8  N   UNK   1        -3.447  -5.076  -0.502  1.00  0.00
HETATM    9  C   UNK   1        -3.425  -3.764  -0.953  1.00  0.00
HETATM   10  C   UNK   1        -1.029   0.592  -0.475  1.00  0.00
HETATM   11  C   UNK   1        -4.728  -5.568  -0.575  1.00  0.00
HETATM   12  C   UNK   1         1.447  -3.613  -0.972  1.00  0.00
HETATM   13  C   UNK   1         2.661  -2.980  -0.696  1.00  0.00
HETATM   14  C   UNK   1         2.661  -1.593  -0.521  1.00  0.00
HETATM   15  C   UNK   1         1.454  -0.873  -0.554  1.00  0.00
HETATM   16  C   UNK   1        -5.543  -4.575  -1.062  1.00  0.00
HETATM   17  C   UNK   1         3.876  -3.780  -0.536  1.00  0.00
HETATM   18  C   UNK   1         1.463   0.587  -0.341  1.00  0.00
HETATM   19  C   UNK   1         5.210  -3.467  -0.737  1.00  0.00
HETATM   20  C   UNK   1         5.987  -4.605  -0.410  1.00  0.00
HETATM   21  N   UNK   1         3.840  -5.086  -0.067  1.00  0.00
HETATM   22  C   UNK   1         5.118  -5.587  -0.001  1.00  0.00
HETATM   23  N   UNK   1         0.213   1.222  -0.312  1.00  0.00
HETATM   24  O   UNK   1         2.544   1.159  -0.210  1.00  0.00
HETATM   25  O   UNK   1        -2.116   1.166  -0.458  1.00  0.00
HETATM   26  C   UNK   1         0.204   2.631  -0.097  1.00  0.00
HETATM   27  C   UNK   1         0.135   3.136   1.227  1.00  0.00
HETATM   28  C   UNK   1         0.127   4.530   1.413  1.00  0.00
HETATM   29  C   UNK   1         0.185   5.400   0.328  1.00  0.00
HETATM   30  C   UNK   1         0.252   4.896  -0.968  1.00  0.00
HETATM   31  C   UNK   1         0.263   3.510  -1.209  1.00  0.00
HETATM   32  C   UNK   1         0.071   2.218   2.445  1.00  0.00
HETATM   33  C   UNK   1         0.338   3.000  -2.644  1.00  0.00
HETATM   34  C   UNK   1        -0.883   3.437  -3.460  1.00  0.00
HETATM   35  C   UNK   1         1.637   3.436  -3.329  1.00  0.00
HETATM   36  C   UNK   1         1.289   2.407   3.355  1.00  0.00
HETATM   37  C   UNK   1        -1.232   2.412   3.226  1.00  0.00
HETATM   38  H   UNK   1        -5.058  -2.483  -1.674  1.00  0.00
```

```
HETATM   39  H   UNK   1        -0.923  -4.683  -1.282  1.00  0.00
HETATM   40  H   UNK   1        -3.155  -1.058  -0.692  1.00  0.00
HETATM   41  H   UNK   1        -2.632  -5.597  -0.151  1.00  0.00
HETATM   42  H   UNK   1        -4.929  -6.584  -0.265  1.00  0.00
HETATM   43  H   UNK   1         1.425  -4.690  -1.139  1.00  0.00
HETATM   44  H   UNK   1         3.593  -1.073  -0.313  1.00  0.00
HETATM   45  H   UNK   1        -6.608  -4.657  -1.230  1.00  0.00
HETATM   46  H   UNK   1         5.586  -2.518  -1.093  1.00  0.00
HETATM   47  H   UNK   1         7.063  -4.696  -0.463  1.00  0.00
HETATM   48  H   UNK   1         2.988  -5.596   0.199  1.00  0.00
HETATM   49  H   UNK   1         5.278  -6.601   0.340  1.00  0.00
HETATM   50  H   UNK   1         0.076   4.951   2.414  1.00  0.00
HETATM   51  H   UNK   1         0.178   6.474   0.493  1.00  0.00
HETATM   52  H   UNK   1         0.296   5.599  -1.796  1.00  0.00
HETATM   53  H   UNK   1         0.085   1.170   2.128  1.00  0.00
HETATM   54  H   UNK   1         0.338   1.906  -2.656  1.00  0.00
HETATM   55  H   UNK   1        -0.856   2.991  -4.460  1.00  0.00
HETATM   56  H   UNK   1        -0.929   4.524  -3.585  1.00  0.00
HETATM   57  H   UNK   1        -1.812   3.116  -2.976  1.00  0.00
HETATM   58  H   UNK   1         1.714   2.989  -4.326  1.00  0.00
HETATM   59  H   UNK   1         2.510   3.114  -2.750  1.00  0.00
HETATM   60  H   UNK   1         1.697   4.523  -3.448  1.00  0.00
HETATM   61  H   UNK   1         1.270   1.681   4.176  1.00  0.00
HETATM   62  H   UNK   1         1.321   3.407   3.799  1.00  0.00
HETATM   63  H   UNK   1         2.220   2.257   2.798  1.00  0.00
HETATM   64  H   UNK   1        -1.300   1.686   4.044  1.00  0.00
HETATM   65  H   UNK   1        -2.102   2.266   2.576  1.00  0.00
HETATM   66  H   UNK   1        -1.306   3.412   3.665  1.00  0.00
HETATM   67  P   UNK   1         0.123  -6.575   1.211  1.00  0.00
HETATM   68  O   UNK   1         1.479  -6.451   0.585  1.00  0.00
HETATM   69  O   UNK   1        -1.178  -6.354   0.501  1.00  0.00
HETATM   70  O   UNK   1         0.073  -5.667   2.519  1.00  0.00
HETATM   71  O   UNK   1         0.094  -7.990   1.944  1.00  0.00
HETATM   72  H   UNK   1        -0.881  -5.517   2.670  1.00  0.00
HETATM   73  H   UNK   1         1.039  -8.206   2.071  1.00  0.00
CONECT    1    9    9   16   38
CONECT    2    3    3    7   15
CONECT    3    2    2    4   12
CONECT    4    3    5    5   39
CONECT    5    4    4    6    9
CONECT    6    5    7    7   40
CONECT    7    2    6    6   10
CONECT    8    9   11   41
CONECT    9    1    1    5    8
CONECT   10    7   23   25   25
CONECT   11    8   16   16   42
CONECT   12    3   13   13   43
CONECT   13   12   12   14   17
CONECT   14   13   15   15   44
CONECT   15    2   14   14   18
CONECT   16    1   11   11   45
CONECT   17   13   19   19   21
CONECT   18   15   23   24   24
CONECT   19   17   17   20   46
CONECT   20   19   22   22   47
CONECT   21   17   22   48
CONECT   22   20   20   21   49
CONECT   23   10   18   26
CONECT   24   18   18
CONECT   25   10   10
CONECT   26   23   27   27   31
CONECT   27   26   26   28   32
CONECT   28   27   29   29   50
CONECT   29   28   28   30   51
```

```
CONECT   30   29   31   31   52
CONECT   31   26   30   30   33
CONECT   32   27   36   37   53
CONECT   33   31   34   35   54
CONECT   34   33   55   56   57
CONECT   35   33   58   59   60
CONECT   36   32   61   62   63
CONECT   37   32   64   65   66
CONECT   38    1
CONECT   39    4
CONECT   40    6
CONECT   41    8
CONECT   42   11
CONECT   43   12
CONECT   44   14
CONECT   45   16
CONECT   46   19
CONECT   47   20
CONECT   48   21
CONECT   49   22
CONECT   50   28
CONECT   51   29
CONECT   52   30
CONECT   53   32
CONECT   54   33
CONECT   55   34
CONECT   56   34
CONECT   57   34
CONECT   58   35
CONECT   59   35
CONECT   60   35
CONECT   61   36
CONECT   62   36
CONECT   63   36
CONECT   64   37
CONECT   65   37
CONECT   66   37
CONECT   67   68   69   69   70   71
CONECT   68   67
CONECT   69   67   67
CONECT   70   67   72
CONECT   71   67   73
CONECT   72   70
CONECT   73   71
MASTER         0    0    0    0    0    0    0    0   73    0   73    0
END


HEADER    Imido-naphthalene host + monohydrogen pyrophosphate guest
COMPND    MMFF94 geometry optimized
AUTHOR    GENERATED BY PCMODEL V 9.30
HETATM    1  C   UNK   1         4.772  -1.889   1.151  1.00  0.00
HETATM    2  C   UNK   1        -0.236  -0.309   0.303  1.00  0.00
HETATM    3  C   UNK   1        -0.207  -1.704   0.480  1.00  0.00
HETATM    4  C   UNK   1         1.025  -2.361   0.639  1.00  0.00
HETATM    5  C   UNK   1         2.239  -1.663   0.616  1.00  0.00
HETATM    6  C   UNK   1         2.197  -0.274   0.452  1.00  0.00
HETATM    7  C   UNK   1         0.973   0.392   0.299  1.00  0.00
HETATM    8  N   UNK   1         3.692  -3.700   0.452  1.00  0.00
HETATM    9  C   UNK   1         3.527  -2.356   0.750  1.00  0.00
HETATM   10  C   UNK   1         0.963   1.857   0.121  1.00  0.00
HETATM   11  C   UNK   1         5.004  -4.063   0.667  1.00  0.00
HETATM   12  C   UNK   1        -1.412  -2.425   0.484  1.00  0.00
HETATM   13  C   UNK   1        -2.651  -1.803   0.289  1.00  0.00
```

```
HETATM   14  C    UNK    1         -2.666  -0.414   0.119  1.00   0.00
HETATM   15  C    UNK    1         -1.473   0.321   0.131  1.00   0.00
HETATM   16  C    UNK    1          5.695  -2.960   1.101  1.00   0.00
HETATM   17  C    UNK    1         -3.901  -2.575   0.254  1.00   0.00
HETATM   18  C    UNK    1         -1.522   1.785  -0.054  1.00   0.00
HETATM   19  C    UNK    1         -5.222  -2.174   0.413  1.00   0.00
HETATM   20  C    UNK    1         -6.059  -3.308   0.289  1.00   0.00
HETATM   21  N    UNK    1         -3.934  -3.945   0.027  1.00   0.00
HETATM   22  C    UNK    1         -5.241  -4.383   0.053  1.00   0.00
HETATM   23  N    UNK    1         -0.291   2.455  -0.058  1.00   0.00
HETATM   24  O    UNK    1         -2.612   2.342  -0.185  1.00   0.00
HETATM   25  O    UNK    1          2.027   2.475   0.143  1.00   0.00
HETATM   26  C    UNK    1         -0.319   3.868  -0.243  1.00   0.00
HETATM   27  C    UNK    1         -0.241   4.402  -1.556  1.00   0.00
HETATM   28  C    UNK    1         -0.270   5.799  -1.713  1.00   0.00
HETATM   29  C    UNK    1         -0.373   6.645  -0.613  1.00   0.00
HETATM   30  C    UNK    1         -0.449   6.112   0.671  1.00   0.00
HETATM   31  C    UNK    1         -0.424   4.723   0.884  1.00   0.00
HETATM   32  C    UNK    1         -0.129   3.508  -2.788  1.00   0.00
HETATM   33  C    UNK    1         -0.510   4.178   2.307  1.00   0.00
HETATM   34  C    UNK    1          0.682   4.634   3.155  1.00   0.00
HETATM   35  C    UNK    1         -1.834   4.560   2.975  1.00   0.00
HETATM   36  C    UNK    1         -1.334   3.680  -3.718  1.00   0.00
HETATM   37  C    UNK    1          1.183   3.751  -3.540  1.00   0.00
HETATM   38  H    UNK    1          4.988  -0.877   1.462  1.00   0.00
HETATM   39  H    UNK    1          1.013  -3.449   0.767  1.00   0.00
HETATM   40  H    UNK    1          3.123   0.293   0.422  1.00   0.00
HETATM   41  H    UNK    1          2.960  -4.341   0.109  1.00   0.00
HETATM   42  H    UNK    1          5.290  -5.091   0.489  1.00   0.00
HETATM   43  H    UNK    1         -1.361  -3.508   0.633  1.00   0.00
HETATM   44  H    UNK    1         -3.610   0.098  -0.041  1.00   0.00
HETATM   45  H    UNK    1          6.743  -2.936   1.359  1.00   0.00
HETATM   46  H    UNK    1         -5.547  -1.164   0.613  1.00   0.00
HETATM   47  H    UNK    1         -7.135  -3.343   0.372  1.00   0.00
HETATM   48  H    UNK    1         -3.119  -4.559  -0.149  1.00   0.00
HETATM   49  H    UNK    1         -5.427  -5.439  -0.093  1.00   0.00
HETATM   50  H    UNK    1         -0.213   6.239  -2.706  1.00   0.00
HETATM   51  H    UNK    1         -0.393   7.721  -0.756  1.00   0.00
HETATM   52  H    UNK    1         -0.528   6.795   1.514  1.00   0.00
HETATM   53  H    UNK    1         -0.120   2.454  -2.491  1.00   0.00
HETATM   54  H    UNK    1         -0.477   3.084   2.296  1.00   0.00
HETATM   55  H    UNK    1          0.650   4.165   4.145  1.00   0.00
HETATM   56  H    UNK    1          0.693   5.719   3.303  1.00   0.00
HETATM   57  H    UNK    1          1.629   4.351   2.682  1.00   0.00
HETATM   58  H    UNK    1         -1.929   5.642   3.116  1.00   0.00
HETATM   59  H    UNK    1         -1.916   4.091   3.962  1.00   0.00
HETATM   60  H    UNK    1         -2.686   4.225   2.373  1.00   0.00
HETATM   61  H    UNK    1         -1.280   2.971  -4.551  1.00   0.00
HETATM   62  H    UNK    1         -1.385   4.688  -4.144  1.00   0.00
HETATM   63  H    UNK    1         -2.271   3.495  -3.181  1.00   0.00
HETATM   64  H    UNK    1          2.044   3.617  -2.876  1.00   0.00
HETATM   65  H    UNK    1          1.237   4.762  -3.959  1.00   0.00
HETATM   66  H    UNK    1          1.286   3.044  -4.370  1.00   0.00
HETATM   67  P    UNK    1          1.531  -6.823  -0.025  1.00   0.00
HETATM   68  O    UNK    1          2.393  -8.008  -0.423  1.00   0.00
HETATM   69  O    UNK    1          1.959  -5.449  -0.506  1.00   0.00
HETATM   70  O    UNK    1          1.519  -6.775   1.587  1.00   0.00
HETATM   71  O    UNK    1         -0.014  -7.158  -0.317  1.00   0.00
HETATM   72  H    UNK    1          0.637  -6.309   1.796  1.00   0.00
HETATM   73  P    UNK    1         -1.320  -6.552   0.415  1.00   0.00
HETATM   74  O    UNK    1         -0.776  -5.906   1.684  1.00   0.00
HETATM   75  O    UNK    1         -1.945  -5.552  -0.545  1.00   0.00
HETATM   76  O    UNK    1         -2.229  -7.737   0.683  1.00   0.00
CONECT    1    9    9   16   38
```

```
CONECT    2    3    3    7   15
CONECT    3    2    2    4   12
CONECT    4    3    5    5   39
CONECT    5    4    4    6    9
CONECT    6    5    7    7   40
CONECT    7    2    6    6   10
CONECT    8    9   11   41
CONECT    9    1    1    5    8
CONECT   10    7   23   25   25
CONECT   11    8   16   16   42
CONECT   12    3   13   13   43
CONECT   13   12   12   14   17
CONECT   14   13   15   15   44
CONECT   15    2   14   14   18
CONECT   16    1   11   11   45
CONECT   17   13   19   19   21
CONECT   18   15   23   24   24
CONECT   19   17   17   20   46
CONECT   20   19   22   22   47
CONECT   21   17   22   48
CONECT   22   20   20   21   49
CONECT   23   10   18   26
CONECT   24   18   18
CONECT   25   10   10
CONECT   26   23   27   27   31
CONECT   27   26   26   28   32
CONECT   28   27   29   29   50
CONECT   29   28   28   30   51
CONECT   30   29   31   31   52
CONECT   31   26   30   30   33
CONECT   32   27   36   37   53
CONECT   33   31   34   35   54
CONECT   34   33   55   56   57
CONECT   35   33   58   59   60
CONECT   36   32   61   62   63
CONECT   37   32   64   65   66
CONECT   38    1
CONECT   39    4
CONECT   40    6
CONECT   41    8
CONECT   42   11
CONECT   43   12
CONECT   44   14
CONECT   45   16
CONECT   46   19
CONECT   47   20
CONECT   48   21
CONECT   49   22
CONECT   50   28
CONECT   51   29
CONECT   52   30
CONECT   53   32
CONECT   54   33
CONECT   55   34
CONECT   56   34
CONECT   57   34
CONECT   58   35
CONECT   59   35
CONECT   60   35
CONECT   61   36
CONECT   62   36
CONECT   63   36
CONECT   64   37
CONECT   65   37
```

```
CONECT   66   37
CONECT   67   68   69   69   70   71
CONECT   68   67
CONECT   69   67   67
CONECT   70   67   72
CONECT   71   67   73
CONECT   72   70
CONECT   73   71   74   75   76   76
CONECT   74   73
CONECT   75   73
CONECT   76   73   73
MASTER         0    0    0    0    0    0    0    0   76    0   76    0
    END
```
